# Design and Modulation of Selectivity toward Vanadium(V) and Uranium(VI) Ions: Coordination Properties and Affinity of Hydroxylamino-Triazine Siderophores

**DOI:** 10.1021/acs.inorgchem.3c02678

**Published:** 2023-11-29

**Authors:** Angelos Amoiridis, Michael Papanikolaou, Manolis Vlasiou, Nuno A. G. Bandeira, Haralampos N. Miras, Themistoklis Kabanos, Anastasios Keramidas

**Affiliations:** † Department of Chemistry, University of Cyprus, Nicosia 2109, Cyprus; ‡ School of Veterinary Medicine, University of Nicosia, Nicosia 2414, Cyprus; § Biosystems and Integrative Sciences Institute (BioISI) - Departamento de Química e Bioquímica, Faculdade de Ciências Universidade de Lisboa, 8.5.53 - C8 Campo Grande, Lisboa 1749-016, Portugal; ∥ School of Chemistry, The University of Glasgow, Glasgow G12 8QQ, U.K.; ⊥ Department of Chemistry, Section of Inorganic and Analytical Chemistry, University of Ioannina, Ioannina 45110, Greece

## Abstract

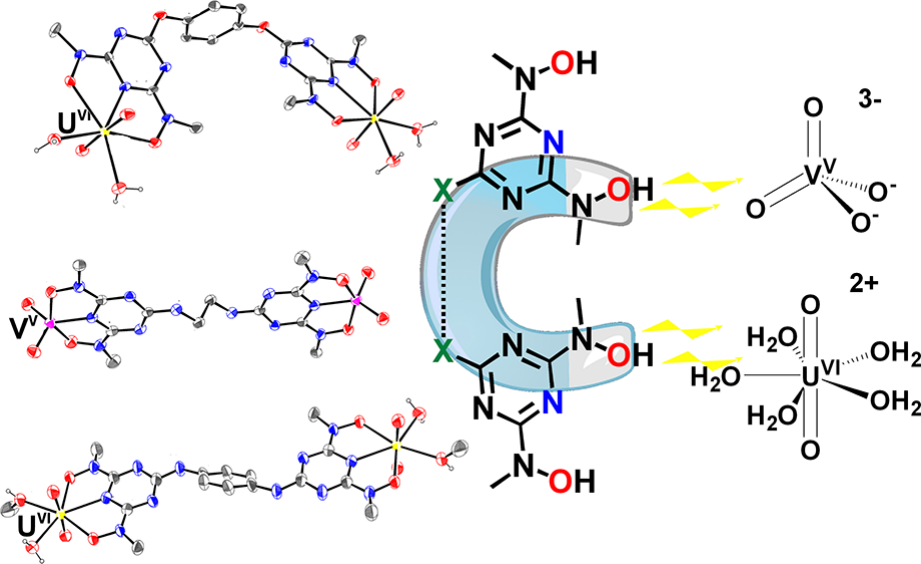

Based on the strong binding and high selectivity properties of 2,6-bis[hydroxy(methyl)amino]-4-morpholino-1,3,5-triazine (H_2_bihyat) for [U^VI^O_2_]^2+^, novel binucleating ligands (BLs) *N*,*N*′,*N*″,*N‴*-((1,4-phenylenebis(oxy))bis(1,3,5-triazine-6,2,4-triyl))tetrakis(*N*-methylhydroxylamine) (H_4_qtn), *N*^1^,*N*^4^-bis(4,6-bis(hydroxy(methyl)amino)-1,3,5-triazin-2-yl)benzene-1,4-diamine (H_4_pdl), and *N*^1^,*N*^2^-bis(4,6-bis(hydroxy(methyl)amino)-1,3,5-triazin-2-yl)ethane-1,2-diamine (H_4_enl) were synthesized. Binuclear complexes formed by coordination of hard metal ions with H_4_qtn are thermodynamically more stable than their mononuclear analogues with H_2_bihyat due to the increase in entropy accompanying the formation of more chelate rings. Reaction of either H_4_qtn or H_4_pdl or H_4_enl with [U^VI^O_2_]^2+^ and [V^V^O_2_]^+^ resulted in the isolation of the binuclear complexes [(U^VI^O_2_)_2_(μ-qtn)(H_2_O)_4_] (**1**), [(V^V^O_2_)_2_(μ-qtn)][PPh_4_]_2_[PPh4] (**2**), [(U^VI^O_2_)_2_(μ-pdl)(H_2_O)_2_(MeOH)_2_] (**3**), [(V^V^O_2_)_2_(μ-pdl)][PPh_4_]_2_ (**4**), [(U^VI^O_2_)_2_(μ-enl)(H_2_O)_4_] (**5**), and [(V^V^O_2_)_2_(μ-enl)][PPh_4_]_2_ (**6**). The binuclear complexes **1–6** were characterized by single-crystal X-ray diffraction analysis in solid state and by NMR and ESI-MS in solution. The comparison of the coordination ability of the BLs with either pyridine-2,6-dicarboxylic acid (H_2_dipic) or H_2_bihyat or CO_3_^2–^ toward [U^VI^O_2_]^2+^ and [V^V^O_2_]^+^ was investigated by NMR and UV–vis spectroscopies and DFT theoretical calculations, revealing a superior performance of BLs. The selectivity of the BLs for [U^VI^O_2_]^2+^ over [V^V^O_2_]^+^ is decreased compared to that of H_2_bihyat but increases considerably at pH > 9 values. Formation of the mixed-metal binuclear species [U^VI^O_2_(μ-O)V^V^O_2_] influences the selectivity and dynamics of the reaction of H_4_qtn for [U^VI^O_2_]^2+^ and [V^V^O_2_]^+^ in aqueous solution. The results of this study provide crucial information for the ligand design and the development of stronger and more selective systems.

## Abstract 2

The binuclear complexes [(U^VI^O_2_)_2_(BL)(H_2_O)_4_] and [(V^V^O_2_)_2_(BL)]^2−^ with the novel hydroxylamino-triazine binucleating ligands H_4_qtn, H_4_pdl, and H_4_enl (BLs) were synthesized and structurally and physicochemically characterized.

## Introduction

**Scheme 1:**
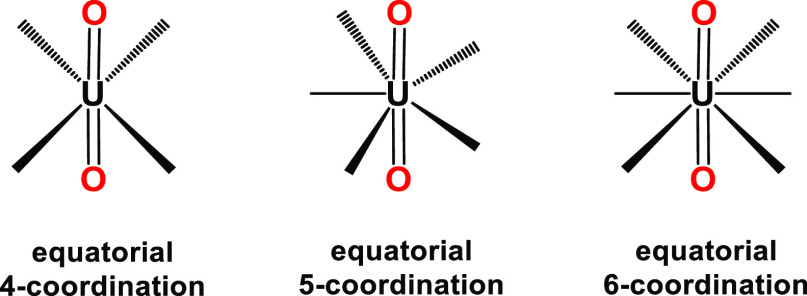
Coordination Modes of the [U^VI^O_2_]^2+^ Structural Unit

**Scheme 2:**
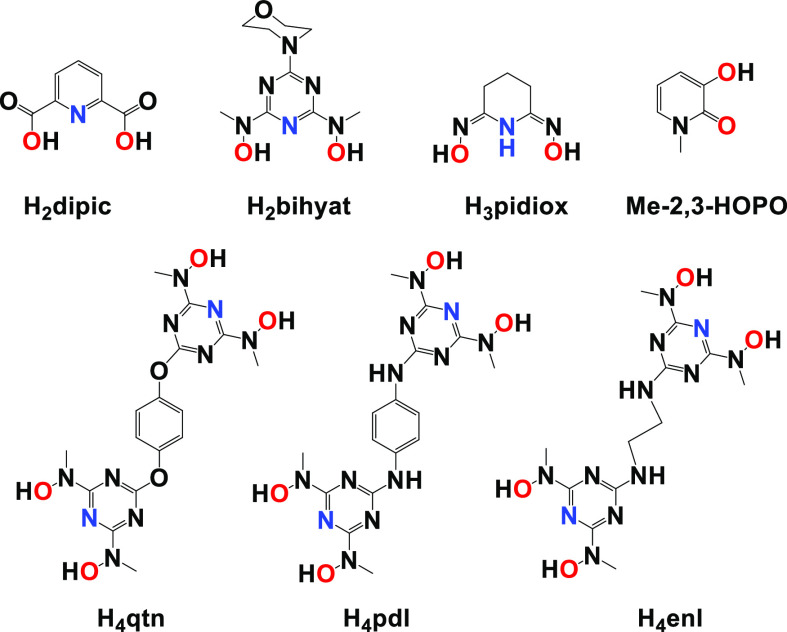
Molecular Drawings of the Ligands: H_2_dipic, H_2_bihyat, H_3_pidiox, Me-2,3-HOPO, H_4_qtn, H_4_pdl, and H_4_enl

In recent years, the synthesis of new siderophores has become the focus of intense scientific research for the development of strong and selective chelators for hard metal ions and their application in removal of toxic metals from the environment and humans and from the radioactive wastes produced by nuclear industries and metal mining from seawater.^1−26^ Amidoxime-containing polymers are considered the most promising candidates for the extraction of uranium from the sea.^27−31^ However, amidoximes lack desirable selectivity for binding uranium in the presence of other hard metal ions, in particular, vanadium(V) and iron(III).^32−39^ In order to improve the ligands’ selectivity for binding [U^VI^O_2_]^2+^, the chelating group has to satisfy the soft–hard acid–base properties and the geometric preferences of the metal ion. The equatorial plane of [U^VI^O_2_]^2+^ is the only one available for coordination, meaning that planar, penta-, or hexadentate hard-donor ligands fulfill the ligation requirements for selective binding of [U^VI^O_2_]^2+^ (Schemes 1 and 2).^26^

A group of nontoxic siderophores based on an *N*,*N*′-disubstituted bis(hydroxyamino)-1,3,5-triazine (BHT) motif form hydrolytically stable complexes with hard metal ions, such as Fe^III^, Ti^IV^, V^V^, U^VI^, and Mo^VI^.^3,10−14,40−42^ The high thermodynamic stability of the hard metal ion complexes with BHT siderophores has been attributed to the hard hydroxylamine oxygen and the negative formal charge of the triazine nitrogen donor atoms. The tridentate planar BHT ligands fit perfectly in the equatorial plane of [U^VI^O_2_]^2+^, thus satisfying the geometric requirement of [U^VI^O_2_]^2+^ for its selective binding. Recently, the thermodynamic stability and selectivity for [U^VI^O_2_]^2+^ over Fe^III^ and [V^V^O_2_]^+^ with the BHT ligand, 2,6-bis[hydroxy(methyl)amino]-4-morpholino1,3,5-triazine (H_2_bihyat; Scheme 2) have been reported.^13^ The selectivity and thermodynamic stability of H_2_bihyat for [U^VI^O_2_]^2+^ were found to be superior in comparison with other hard-donor ligands, such as pyridine-2,6-dicarboxylic acid (H_2_dipic; Scheme 2) and amidoxime (H_3_pidiox, Scheme 2), dictating BHT ligands as the best candidates for sequestration of [U^VI^O_2_]^2+^ from the sea.

Herein, we report the synthesis of the binucleating BHT-type ligands (BLs), *N*,*N*′,*N*″,*N*‴-((1,4-phenylenebis(oxy))bis(1,3,5-triazine-6,2,4-triyl))tetrakis(*N*-methylhydroxylamine) (H_4_qtn), *N*^1^,*N*^4^-bis(4,6-bis(hydroxy(methyl)amino)-1,3,5-triazin-2-yl)benzene-1,4-diamine (H_4_pdl), and *N*^1^,*N*^2^-bis(4,6-bis(hydroxy(methyl)amino)-1,3,5-triazin-2-yl)ethane-1,2-diamine (H_4_enl) (Scheme 2) and the syntheses and structural and solution characterizations of six new binuclear uranyl and vanadate(V) complexes, [(U^VI^O_2_)_2_(μ-qtn)(H_2_O)_4_] (**1**), [(V^V^O_2_)_2_(μ-qtn)][PPh_4_]_2_ (**2**), [(U^VI^O_2_)_2_(μ-pdl)(H_2_O)_2_(MeOH)_2_] (**3**), [(V^V^O_2_)_2_(μ-pdl)][PPh_4_]_2_ (**4**), [(U^VI^O_2_)_2_(μ-enl)(H_2_O)_4_] (**5**), and [(V^V^O_2_)_2_(μ-enl)][PPh_4_]_2_ (**6**). The BLs have been designed to favor the binding of the metal ions by increasing the entropy of the system through the formation of more chelate rings than H_2_bihyat. By increasing the nucleating sites from one to two, although far less than the multiple binding sites in a polymeric material, we mimic a polymer better, keeping the compounds small and easier to study. Thus, the information that will be obtained from the interaction of BLs with the metal ions will give us a better insight of how to make the polymeric materials used for the selective binding of metal ions more effective. In addition, the bridging moieties have been chosen to be either aliphatic so that the two metal ions are isolated or aromatic so that the two metal ions might interact with each other controlling the thermodynamic stability of the complexes. The BLs are of the strongest binders for [U^VI^O_2_]^2+^ and [V^V^O_2_]^+^, to be reported. The selectivity of BLs for [U^VI^O_2_]^2+^ and [V^V^O_2_]^+^ is pH-dependent, and the equilibrium is shifted toward [U^VI^O_2_]^2+^ at high pHs (>7). However, in aqueous solution, the reaction of [U^VI^O_2_]^2+^ and [V^V^O_2_]^+^ with BLs results in the formation of **1–6** and [(U^VI^O_2_)(V^V^O_2_)(μ-BL)(H_2_O)_2_]^–^ and U^VI^–μ-O**–**V^V^ species which influence the selectivity and kinetics of the reactions.

## Experimental Section

### Synthesis of (1,4-Bis((4,6-dichloro-1,3,5-triazin-2-yl)oxy)benzene) (**qtCl**_**4**_)

To a vigorously stirred solution of cyanuric chloride (7.302 g, 40.00 mmol) in THF (40 mL) at 0 °C, a solution of 1,4-hydroquinone (1.981 g, 18.00 mmol) and *N*,*N*-diisopropylethylamine (DIPEA) (5.169 g, 40.00 mmol) in THF (50 mL) was added dropwise, and a white solid was formed upon its addition. The reaction mixture was stirred overnight at room temperature (22 °C). The solvent was evaporated to dryness, and the white solid was dried under vacuum and triturated with distilled water (40 mL) under magnetic stirring for 2 h. The mixture was filtered, and the dried solid was recrystallized with ethyl acetate. The crystalline white solid was filtered and dried to get 3.362 g of the desired product. Yield: 46% (based on 1,4-hydroquinone). ^1^H NMR (CDCl_3_, 500 MHz, 25 °C) δ (ppm): 7.30 (s, 4H, from hydroquinone ring); ^13^C NMR (CDCl_3_, 500 MHz, 25 °C) δ (ppm): 173.27, 170.90, 149.06, 122.69. Anal. calcd for C_12_H_6_N_8_Cl_4_ (*M*_r_ = 404.95): C, 35.50; H, 0.99; N, 20.70; anal. found: C, 35.41; H, 1.08; N, 20.69.

### Synthesis of (*N*^1^,*N*^4^-Bis(4,6-dichloro-1,3,5-triazin-2-yl)benzene-1,4-diamine) (**pdCl**_**4**_)

pdCl_4_ was synthesized using the method reported for qtCl_4_ using 1,4-phenyldiamine instead of hydroquinone. In this case acetone was used as the solvent instead of THF. Yield: 75% (based on 1,4-phenyldiamine). ^1^H NMR (DMSO, 500 MHz, 25 °C) δ (ppm): 11.18 (s, 2H, NH), 7.60 (s, 4H, C_6_H_4_); ^13^C NMR (DMSO, 125 MHz, 25 °C) δ (ppm): 169.69, 168.76, 163.73, 133.70, 122.08. Anal. calcd for C_12_H_6_Cl_4_N_8_ (*M*_r_ = 355.99): C, 35.67; H, 1.50; N, 27.73; anal. found: C, 35.74; H, 1.55; N, 27.32.

### Synthesis of (*N*^1^,*N*^2^-Bis(4,6-dichloro-1,3,5-triazin-2-yl)ethane-1,2-diamine) (**enCl**_**4**_)

enCl_4_ was synthesized using the method reported for pdCl_4_ using 1,2-diethylamine instead of hydroquinone_._ Yield: 99% (based on ethylenediamine). ^1^H NMR (DMSO, 500 MHz, 25 °C) δ (ppm): 9.16 (s, 2H, −NH), 3.47 (s, 4H, −C_2_H_4_); ^13^C NMR (DMSO, 125 MHz, 25 °C) δ (ppm): 169.74, 169.64, 168.90, 39.84. Anal. calcd for C_8_H_6_Cl_4_N_8_ (*M*_r_ = 406.00): C, 26.99; H, 1.50; N, 27.73; anal. found: C, 35.74; H, 1.55; N, 27.32.

### Synthesis of (*N*,*N*′,*N*″,*N‴*-((1,4-Phenylenebis(oxy))bis(1,3,5-triazine-6,2,4-triyl))tetrakis(*N*-methylhydroxylamine)) (**H**_**4**_**qtn**)

To a solution of *N*-methylhydroxylamine hydrochloride (1.563 g, 18.72 mmol) in distilled water (2 mL), a solution of NaOH (0.749 g, 18.72 mmol) in distilled water (2 mL) was added dropwise at 0 °C. The resulting solution was added dropwise to a solution of qtCl_4_ (0.950 g, 2.34 mmol) in THF (30 mL) at 0 °C. Upon addition of qtCl_4_, a white solid was formed. The mixture was refluxed overnight, and then the white solid was filtered off, washed with the minimum amount of THF (5 mL) and distilled water (5 mL), and dried under vacuum to give 0.755 g of H_4_qtn. Yield: 72% (based on qtCl_4_). ^1^H NMR (D_2_O, 500 MHz, 25 °C) δ (ppm): 7.20 (s, 4H, C_6_H_4_), 3.20 (s, 12 H, N–CH_3_); ^13^C NMR (D_2_O, 500 MHz, 25 °C) δ (ppm): 181.48, 168.31, 149.04, 122.27, 37.84. Anal. Calcd for C_16_H_20_N_10_O_6_ (*M*_r_ = 448.40): C, 42.86; H, 4.50; N, 31.24; Anal. Found: C, 42.75; H, 4.71; N, 32.07.

#### Synthesis of (*N*^1^,*N*^4^-Bis(4,6-bis(hydroxy(methyl)amino)-1,3,5-triazin-2-yl)benzene-1,4-diamine) (**H**_**4**_**pdl**)

To a stirred, ice-cold mixture of pdCl_4_ (1.00 g, 2.47 mmol) in 20 mL of 1,6-dioxane, an aqueous solution (2 mL) of hydroxylamine hydrochloride (1.23 g, 14.8 mmol) and sodium hydroxide (0.59 g, 15 mmol) was added dropwise. The mixture was stirred for 24 h at room temperature. Then, 50 mL of distilled water was added, and the pH was neutralized using a 2 M solution of NaOH. The solid was collected by filtration, washed with diethyl ether (10 mL) and distilled water (5 mL), and dried under vacuum to yield 0.94 g of a light-yellow powder of H_4_pdl. Yield: 96% (based on pdCl_4_). ^1^H NMR (D_2_O, pD > 7500 MHz, 25 °C) δ (ppm): 7.46 (s, 4H, C_6_H_4_), 3.33 (s, 12 H N–CH_3_); ^13^C NMR (D_2_O, pD > 7, 125 MHz, 25 °C) δ (ppm): 168.36, 160.48, 134.20, 121.92, 37.50. Anal. calcd for C_16_H_22_N_12_O_4_ (*M*_r_ = 446.43): C, 43.05; H, 4.97; N, 37.65; anal. found: C, 42.95; H, 4.84; N, 37.87.

### Synthesis of (*N*^1^,*N*^2^-Bis(4,6-bis(hydroxy(methyl)amino)-1,3,5-triazin-2-yl)ethane-1,2-diamine)) (**H**_**4**_**enl**)

H_4_enl was synthesized according to the procedure reported for H_4_qtn using enCl_4_ instead of qtCl_4_. ^1^H NMR (D_2_O, pD > 7, 500 MHz, 25 °C) δ (ppm): 3.43 (s, 4H, −C_2_H_4_), 3.15 (s, 12 N–CH_3_); ^13^C NMR (D_2_O, pD > 7, 125 MHz, 25 °C) δ (ppm): 168.30, 162.70, 40.42, 37.17. Anal. calcd for C_12_H_22_N_12_O_4_ (*M*_r_ = 398.39): C, 36.18; H, 5.57; N, 42.19; anal. found: C, 36.38; H, 5.86; N, 42.55.

### Synthesis of [(U^VI^O_2_)_2_(μ-qtn)(H_2_O)_4_]·3H_2_O (**1·3H_2_O**)

To a stirred suspension of H_4_qtn (0.0046 g, 0.010 mmol) in water (2.0 mL) was added a solution of KOH 2 M (20.0 μL, 0.040 mmol). Upon addition of KOH, the solution became clear. To this solution, solid [U^VI^O_2_(NO_3_)_2_(H_2_O)_2_]·4H_2_O (0.0100 g, 0.020 mmol) was added, and the color changed from colorless to dark brown. After 1 day, small brown crystals were formed which were dissolved again by heating the solution at 100 °C. The solution was left undisturbed at room temperature (25 °C), and after 1 day, 0.0061 g of dark brown needles were formed. Yield: 58% (based on H_4_qtn). Anal. calcd for C_16_H_30_N_10_O_17_U_2_ (*M*_r_ = 1110.52): C, 17.30; H, 2.72; N,12.61; anal. found: C, 17.33; H, 2.65; N, 12.49.

### Synthesis of [(U^VI^O_2_)_2_(μ-qtn)(H_2_O)_4_]·2H_2_O·EtOH (**1′**·2H_2_O·EtOH)

Sequential addition of H_4_qtn (0.0046 g, 0.010 mmol), [U^VI^O_2_(NO_3_)_2_(H_2_O)_2_]·4H_2_O (0.0100 g, 0.020 mmol) and triethylamine (6.0 μL, 0.040 mmol) to a stirred ethanol (2.0 mL) solution and boiling of it for 1 min yielded a dark brown solution. The solution was left at room temperature (22 °C) undisturbed for 1 month, upon which time dark brown crystals were formed suitable for single-crystal X-ray diffraction analysis. The crystals were filtered and dried under vacuum. The yield was 0.0060 g (53% based on H_4_qtn). Anal. calcd for C_18_H_34_N_10_O_17_U_2_ (*M*_r_ = 1138.58): C, 18.99; H, 3.01; N,12.30; anal. found: C, 18.78; H, 3.11; N, 12.41.

### Synthesis of [(V^V^O_2_)_2_(μ-qtn)][PPh_4_]_2_·2H_2_O (**2·2H_2_O**)

NaV^V^O_3_ (0.0024 g, 0.020 mmol) was dissolved in distilled water (1.000 mL), and solid H_4_qtn (0.0046 g, 0.01 mmol) was added to it. The mixture was heated to 100 °C, the ligand was dissolved, and the solution’s color became yellow. The solution was left to reach room temperature, and then solid PPh_4_Cl (0.0075 g, 0.02 mmol) was added to it. The mixture was heated to boil until all solids were dissolved. The solution was cooled down to room temperature, and 0.0071 g of yellow crystals suitable for single-crystal X-ray diffraction analysis were formed. Yield: 55% (based on H_4_qtn). Anal. calcd for C_64_H_56_N_10_O_10_P_2_V_2_ (*M*_r_ = 1325.05): C, 58.01; H, 4.56; N, 10.57; anal. found: C, 58.17; H, 4.65; N, 10.48.

### Synthesis of [(U^VI^O_2_)_2_(μ-pdl)(H_2_O)_2_(MeOH)_2_]·3H_2_O·MeOH (**3**·3H_2_O·MeOH)

A mixture of H_4_pdl (0.0200 g, 0.0449 mmol) and [U^VI^O_2_(CH_3_COO)_2_]·2H_2_O (0.0190 g, 0.0449 mmol) in 5 mL of methanol was stirred for 2 h at room temperature to give a brown solid. The solid was filtered off, washed with methanol (5 mL) and diethyl ether (5 mL), and dried under vacuum. Yield: 87% (based on H_4_pdl). Single crystals suitable for X-ray analysis were obtained by vapor diffusion of methanol into a concentrated aqueous solution of the isolated brown powder. Anal. calcd for C_19_H_40_N_12_O_16_U_2_(*M*_r_ = 1168.65): C, 19.53; H, 3.45; N, 14.38; anal. found: C, 19.38; H, 3.59; N, 14.12.

### Synthesis of [(V^V^O_2_)_2_(μ-pdl)][PPh_4_]_2_ (**4**)

NaV^V^O_3_ (0.0024 g, 0.02 mmol) was dissolved in water (1.000 mL), a solution containing H_4_pdl (0.0050 g, 0.01 mmol) and NaOH (0.0016 g, 0.04 mmol) in H_2_O (2.000 mL) was added to it, and its color turned yellow. Solid PPh_4_Cl (0.0075 g, 0.02 mmol) was added to the stirred solution in one portion. The solution was left undisturbed and within an hour, X-ray quality single crystals of **4** were formed. Yield: 40% (based on H_4_pdl). Anal. calcd for C_64_H_58_N_12_O_8_P_2_V_2_ (*M*_r_ = 1287.08): C, 59.72; H, 4.54; N, 13.06; anal. found: C, 59.33; H, 4.78; N, 13.37.

### Synthesis of [(U^VI^O_2_)_2_(μ-enl)(H_2_O)_4_]·3H_2_O (**5·3H_2_O**)

Complex **5** was synthesized according to the procedure reported for **3** by reacting [U^VI^O_2_(CH_3_COO)_2_]·2H_2_O with H_4_enl instead of H_4_pdl. Yield: 82% (based on H_4_enl). Anal. calcd for C_12_H_32_N_12_O_15_U_2_ (*M*_r_ = 1060.51): C, 13.59; H, 3.04; N, 15.85; anal. found: C, 13.45; H, 2.93; N, 13.65.

### Synthesis of [(V^V^O_2_)_2_(μ-enl)][PPh_4_]_2_ (**6**)

Complex **6** was synthesized according to the procedure reported for **4** by reacting NaV^V^O_3_ with H_4_enl instead of H_4_pdl. In order to have crystals suitable for X-ray analysis, the crystalline solid was filtered, dried under vacuum, and dissolved in methanol. This solution was slowly diffused with diethyl ether vapors at 4 °C resulting in yellow needles. Yield: 71% (based on H_4_enl). Anal. calcd for C_61_H_60_N_12_O_8_P_2_V_2_ (*M*_r_ = 1252.30): C, 58.47; H, 4.83; N, 13.41; anal. found: C, 58.23; H, 4.99; N, 13.65.

## Results and Discussion

### Synthesis of the Ligand H_4_qtn and Compounds **1–6**

**Scheme 3:**
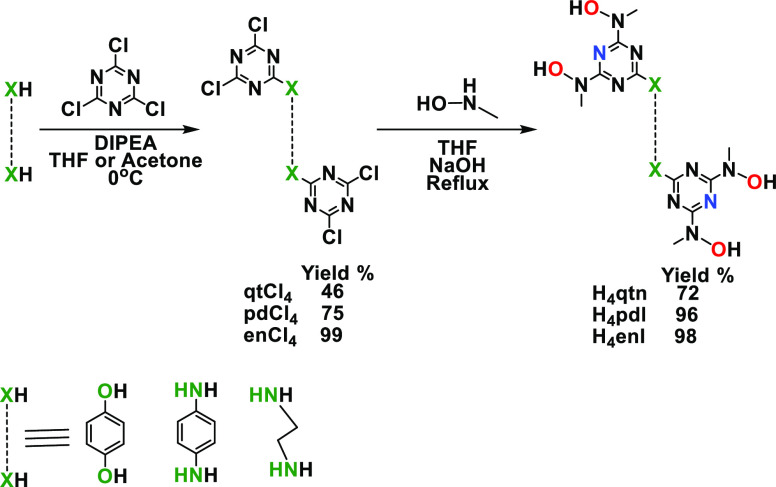
Synthesis of the Organic Molecules qtCl_4_ and H_4_qtn Used in This Study

The synthesis of the ligands is depicted in Scheme 3 and is based on the nucleophilic substitution of cyanuric chloride and takes place in two steps. The first step involves the reaction of the bridging group (either 1,4-hydroquinone or 1,4-phenyldiamine or ethylenediamine) with cyanuric chloride at 0 °C and in the presence of DIPEA to afford qtCl_4_, pdCl_4_, and enCl_4_. Temperature should be kept strictly at 0 °C to avoid further substitution of the triazine. The second step involves the substitution of the remaining four chlorine atoms by reacting qtCl_4_, pdCl_4_, and enCl_4_ with an excess of *N*-methylhydroxylamine at basic solutions. The organic ligands (H_4_qtn, H_4_pdl, and H_4_enl) are insoluble in aqueous solution in a wide range of pHs 3–10 (Figure S1). It is worth mentioning here that the right choice of the solvents for the synthesis of the ligands is very important in order to obtain pure products and high reaction yields.

**Scheme 4:**
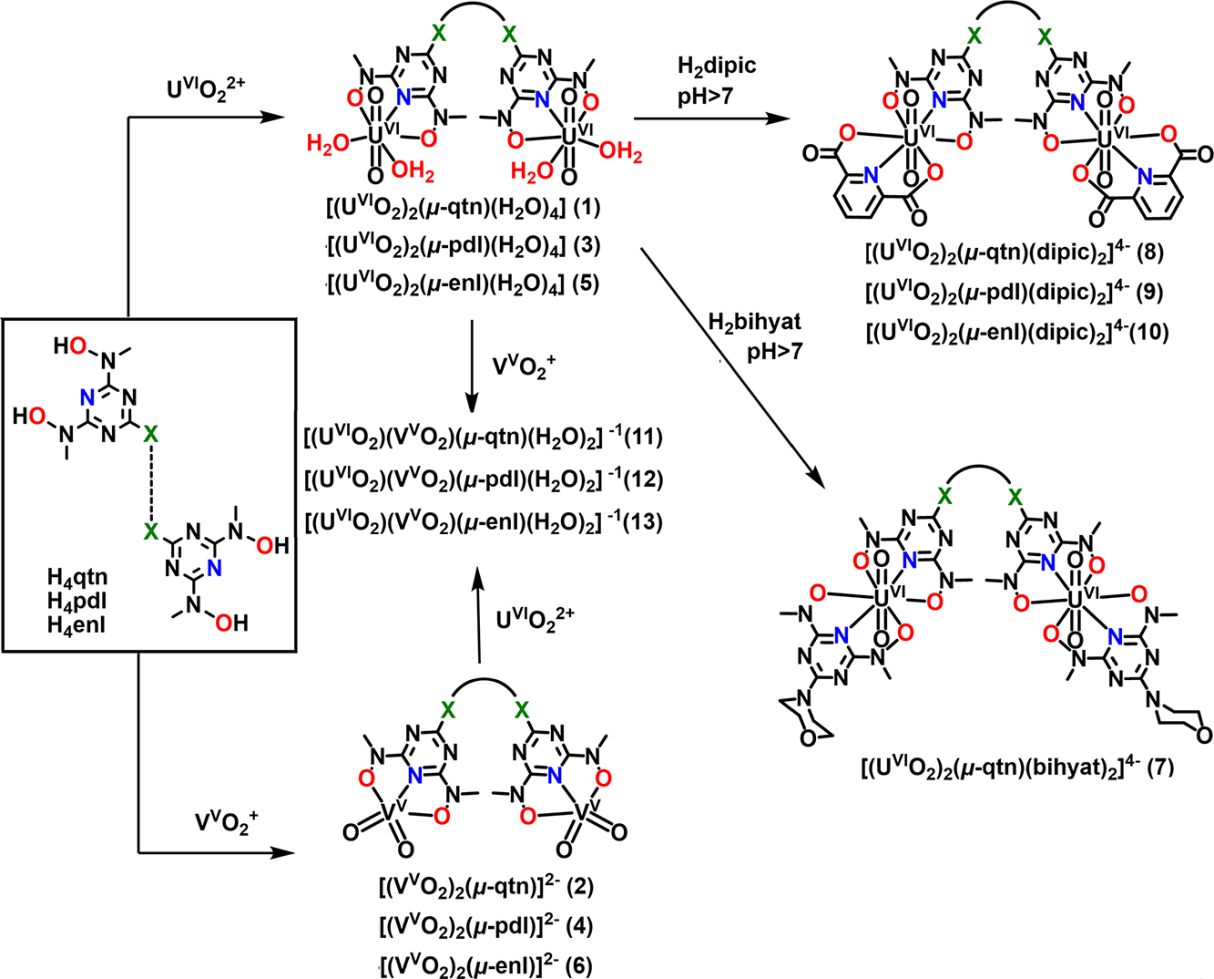
Synthesis of the Binuclear Complexes **1–6** of the Heteroleptic Uranium(VI) Complexes **7–10** and of the Heterometallic (U^VI^/V^V^) Complexes **11–13**

The binuclear uranium(VI) complexes **1**, **1′**, **3**, and **5** were synthesized in a one-pot reaction according to Scheme 4. [U^VI^O_2_(NO_3_)_2_(H_2_O)_2_]·4H_2_O or [U^VI^O_2_(CH_3_COO)_2_]·2H_2_O reacted with the BLs in aqueous or alcoholic solutions. When [U^VI^O_2_(NO_3_)_2_(H_2_O)_2_]·4H_2_O was used as a starting material, KOH was added to the solution with a molar ratio of [U^VI^O_2_]^2+^:H_4_qtn:KOH 2:1:4. The synthesis of the binuclear vanadium(V) complexes **2**, **4**, and **6** was accomplished by reacting an aqueous solution of NaV^V^O_3_ with BLs (V^V^O_2_^+^:BL 2:1) (Scheme 4). After the end of the reaction, PPh_4_Cl was added to the solution resulting in the precipitation of complexes **2**, **4**, and **6**. The U^VI^ and V^V^ binuclear complexes are soluble in water at pH ≥ 7. At pH < 7 the dissolved complexes precipitate out. Thus, all solution studies were performed at pHs 7, 9, 10, and 11. For practical reasons, the stability investigations were conducted at pH 9 or 10 to allow the BLs to dissolve in water. The findings demonstrate that production of the [U^VI^O_2_^2+^–OH] species at high pHs (>8.5) is the only distinction between pHs 7 and 9. The stability of the complexes increases as the pH decreases, but the reactivity is unchanged at either pH. Therefore, the information from this study done at pH 9 or 10 can be extrapolated to pH 7.

Compounds **7–13** were synthesized in solution and characterized by ^1^H NMR and MS. The heteroleptic complexes **7–10** were synthesized by adding 2 equivalents of either H_2_bihyat (**7**) or H_2_dipic (**8–10**) to 1 equivalent of aqueous solutions of the complexes **1**, **3**, or **5** (Scheme 4). The heterometallic compounds **11–13** formed in solution after the addition of V^V^O_4_^3–^ in the aqueous solution of **1**, **3**, or **5** or [U^VI^O_2_]^2+^ in the aqueous solution of **2**, **4**, or **6**. Both homometallic and bimetallic complexes are present in the solution after the reaction (Scheme 4).

### Characterization of the Complexes

#### X-ray Crystallographic Results

**Figure 1:**
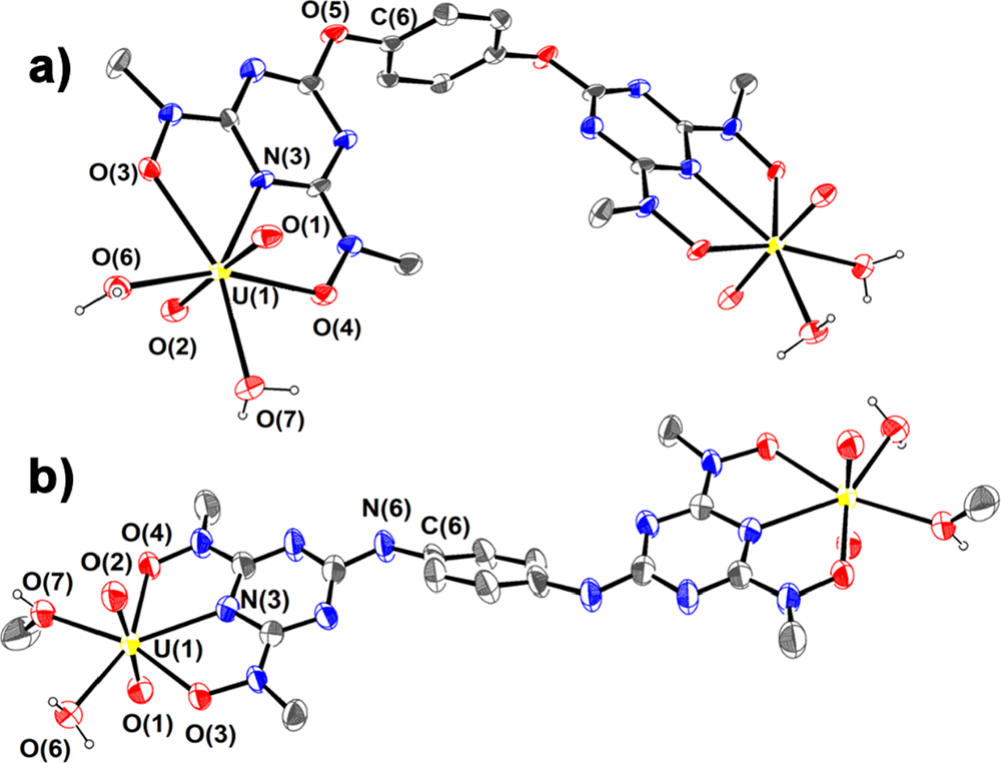
ORTEP plots of (a) **1** and (b) **3** with 50% thermal ellipsoids. Only hydrogen atoms attached to O(6) and O(7) are shown for clarity.

**Figure 2:**
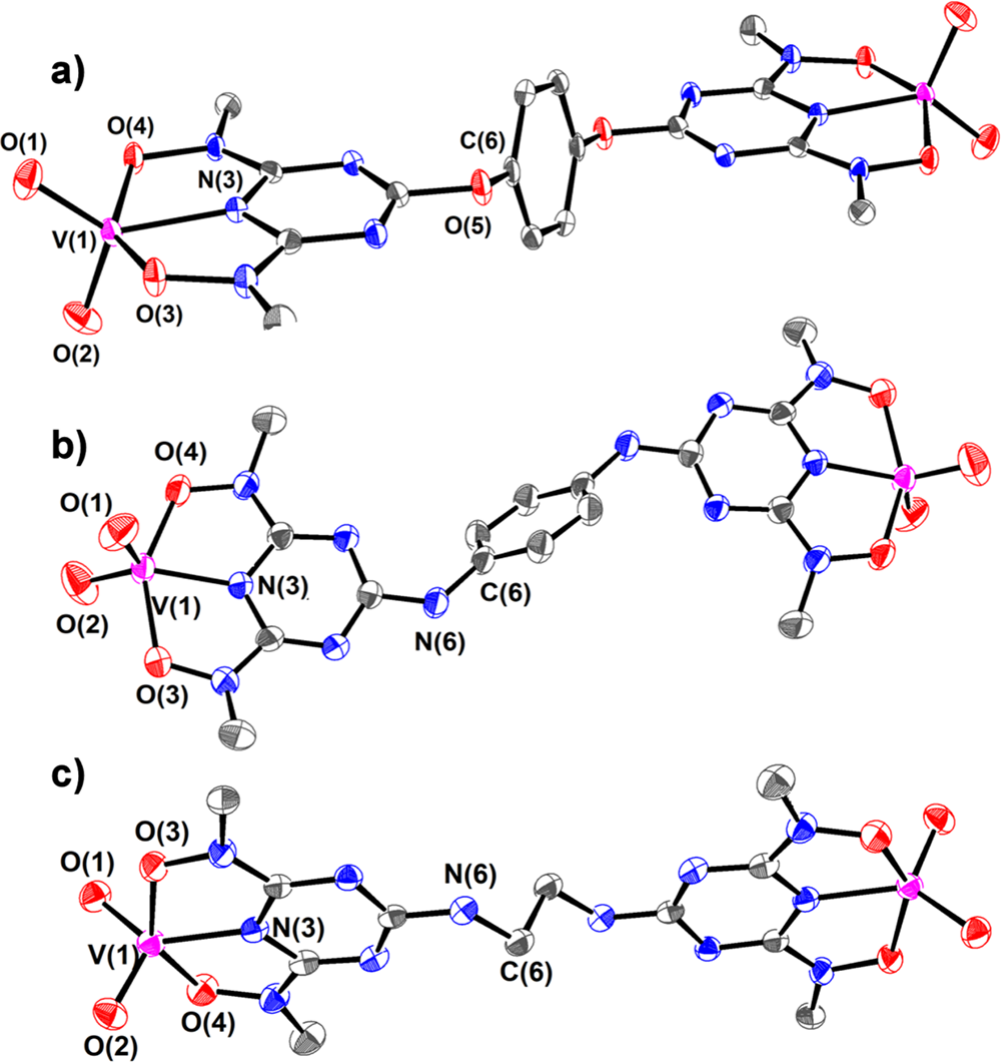
ORTEP plots of the anion of (a) **2**, (b) **4**, and (c) **6** with 50% thermal ellipsoids. Hydrogen atoms are omitted for clarity.

A summary of the crystallographic data and the final refinement details for binuclear complexes **1–4** and **6** are given in Tables S1 and S2. Interatomic distances and bond angles relevant to the U^VI^ and V^V^ coordination spheres are listed in Tables S3 and S4. ORTEP plots of the crystal structures of the binuclear complexes **1**, **3** and **2**, **4**, **6** are shown in Figures 1 and 2 respectively.

Each uranium(VI) atom in complexes **1** and **3** adopts a pentagonal bipyramidal structure with the terminal oxido groups O(1) and O(2) [*d*_mean_(U=O) ∼ 1.775 Å] occupying the two axial positions, whereas the triazine nitrogen atom N(3) [*d*_mean_(U–N_tr_) ∼ 2.438 Å], the two deprotonated hydroxylamine hydroxyls O(3) and O(4) [*d*_mean_(U–O_h_) ∼ 2.390 Å], and the two oxygen atoms O(6) and O(7) [*d*_mean_(U–O_w_) ∼ 2.371 Å] of two water molecules lie in the equatorial plane. The uranium(VI) atom is displaced above the equatorial plane by only ∼0.012 Å. The equatorial plane of the structure is perpendicular to the linear [U^VI^O_2_]^2+^ moiety [(O=U=O)_mean_ ∼ 176.0°].

**Scheme 5:**
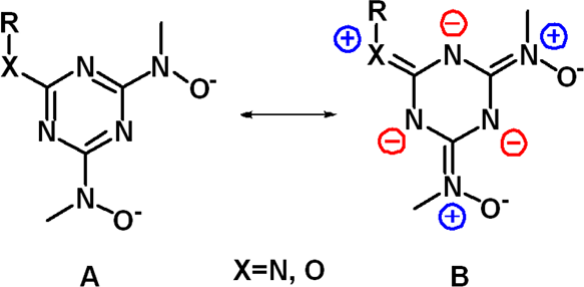
Resonance Structures of the BLs

This is the second crystallographically characterized example of an uranium complex incorporating a triazine ligand, which also shows the bonds between the U^VI^ ion and triazine nitrogen atom (∼2.43 Å) are much stronger than the bonds between [U^VI^O_2_]^2+^ and other related pyridine type nitrogen atoms (2.52–2.64 Å).^43^ For example, [U^VI^O_2_(dipic)(H_2_O)_2_] has a U^VI^–N_py_ bond length of 2.520(6) Å.^44^ The strong U^VI^–N_tr_ bond is attributed to the resonance structure **B** (Scheme 5) of the BLs. The flat sp^2^-hybridized hydroxylamine nitrogen atoms reveal that conformation **B** contributes mainly to the structure of the complex. In conformation **B**, the hydroxylamine nitrogen atoms are approximately sp^2^ hybridized, and thus the ring nitrogen atoms possess high electron densities. Therefore, a strong electron donation from the triazine nitrogen atom N(3) to uranium(VI) takes place, resulting in a relatively strong U^VI^–N bond. H_4_qtn and H_4_pdl ligands have O [O(5), hydroquinone] and N [N(6), 1,4-phenyldiamine] atoms in the trans position to N(3) in the triazine ring, respectively. However, despite the aromatic character of the triazine, the different atoms in the *para* position to N(3) do not influence the strength of the bond between U^VI^ and the triazine nitrogen atom, resulting in indistinguishable U^VI^–N(3) bond distances. The C(5)–O(5)–C(6) and the C(50)–N(6)–C(6) angles are 117.7° and 128.4°, suggesting that the hydroquinone oxygen and the 1,4-phenylenediamine nitrogen atoms are sp^2^ hybridized.

ORTEP plots of the crystal structures of **2**, **4**, and **6** are shown in Figure 2. Each vanadium(V) atom adopts a distorted square pyramidal geometry (τ = 0.215, 0.267, and 0.217 for **2**, **4**, and **6** complexes, respectively; τ = {[O(3)–V(1)–O(4)] – [N(3)–V(1)–O(2)]}/60)^45^ and is bonded to the BLs through the triazine nitrogen atom N(3) [*d*_mean_(V–N_tr_) ∼ 1.997 Å] and the two deprotonated hydroxylamine hydroxyl groups O(3) and O(4) [*d*_mean_(V–O_h_) ∼ 1.985 Å] as well as two cis oxido groups O(1) and O(2) [*d*_mean_(V=O) ∼ 1.631 Å]. The vanadium(V) atom in **2**, **4**, and **6** is displaced above the equatorial plane defined by the hydroxylamine oxygen, the triazine nitrogen, and one of the oxido atoms by 0.543, 0.493, and 0.539 Å, respectively. Similar to the crystal structures of the uranyl complexes, the bond length of V–N_tr_ is one of the shortest reported in the literature, ([V^V^O_2_(dipic)]^−^: *d*(V–N_py_) = 2.096 Å), due to the fact that the ligand is mostly in conformation **B**. In a fashion similar to complexes **1** and **3**, the V–N(3) bond lengths in the complexes **2**, **4**, and **6** are short as the V–N(3) bond distance of [V^V^O_2_(bihyat)]^−^. The C(5)–O(5)–C(6) and C(5)–N(6)–C(6) angles are 117.8°, 126.9°, and 125.2° in complexes **2**, **4**, and **6**, respectively, suggesting that the hydroquinone oxygen, 1,4-phenylenediamine, and ethylenediamine nitrogen atoms have sp^2^ hybridization.

#### IR and UV–Vis Spectroscopies

The solid-state IR spectra of BLs and the binuclear complexes **1–6** are shown in Figures S2–S4. The strong U=O and V=O stretching bands are located between 910 and 940 cm^–1^. These values fall within the expected range for [U^VI^O_2_]^2+^ and [V^V^O_2_]^+^ complexes.^13,14^

The UV–vis spectra of the aqueous solutions of the binuclear complexes **1–6** at various pHs are shown in Figures S5–S7. The UV–vis spectra of the aqueous solutions of the uranyl complexes **1**, **3**, and **5** at pHs 7.0–10.0 exhibit a broad signal covering a region from 300 to 700 nm consistent with the brown color of the solutions assigned to LMCT. The respective spectra of the aqueous solutions of the vanadate complexes **2**, **4**, and **6** also gave a broad band at higher energy ranging from 300 to 500 nm consistent with the light-yellow color of the solutions. The spectra of the binary binuclear complexes **1** and **6** are similar to the UV–vis spectra of the aqueous solutions of [U^VI^O_2_(bihyat)(H_2_O)_2_] and [V^V^O_2_(bihyat)]^−^, respectively. The UV–vis spectra of the aqueous solution of vanadate complexes are the same at pHs 7.0–10.0, revealing that in this pH range, the complexes retain their integrity. On the other hand, the UV–vis spectra of the aqueous solution of the uranyl complexes **1** show that the speciation is altered by increasing the pH from 7.0 to 9.0 (Figures S5–S7).

##### NMR Spectroscopy

The ^1^H NMR of complexes **1–6** and the ^51^V NMR spectra of **2**, **4**, and **6** in solution (D_2_O) at various pDs are shown in Figures S8–S12. The NMR data are summarized in Table 1. The spectra of the uranyl complexes **1**, **3**, and **5** at pD = 7.0 exhibit peaks at 3.489 and 7.264 ppm for **1**, at 3.574, 3.596, and 7.552 ppm for **3**, and at 3.568, 3.574, and 3.605 for **5**, assigned to the hydroxylamine methyl [H(1), H(1′)] and the [H(4)] protons of the bridging ligand, respectively. The peaks are shifted to lower field vs the respective peaks of the free BLs, confirming ligation of the BLs to the [U^VI^O_2_]^2+^ moiety. At pDs > 7, new broad peaks appeared in the ^1^H NMR spectra of the D_2_O solutions of **1**, **3**, and **5** assigned to U^VI^O_2_-(μ-ΟΗ)_2_-U^VI^O_2_^2+^-qtn^4–^ species and confirmed by electrospray ionization mass spectrometry (ESI-MS) (vide infra).^13,46^ This is in agreement with [U^VI^O_2_(bihyat)(H_2_O)_2_], in which high pDs form [U^VI^O_2_(bihyat)_2_]^2–^ and {[U^VI^O_2_(bihyat)(μ–OH)]}_2_^2–^ in aqueous solutions.^13^ The presence of more than one species in solution at pHs > 7 agrees with the UV–vis spectra, which are different at various pHs (vide supra) and are detected by MS (vide infra). In addition, the formation of the charged species U^VI^O_2_-(μ-ΟΗ)_2_-U^VI^O_2_^2+^-qtn^4–^ is supported by the high increase of the solubility in H_2_O similar to that of the neutral [U^VI^O_2_(bihyat)(H_2_O)_2_] at pHs > 7.

**Table 1:** ^1^H (^13^C) Chemical Shifts (ppm) of the D_2_O Solutions at pD = 7.0 of BLs and the Complexes **1–6** (^13^C NMR Shifts from 2D {^1^H, ^13^C} grHMQC)^a^

compound	H_1,1′_(C_1,1_′__)	H_4_(C_4_)	V_1_
H_4_qtn/(D_2_O)	3.198 (37.84)	7.197 (122.28)	
**1/**(D_2_O)	3.489 (37.83)	7.264 (122.72)	
**2/**(MeOD)	3.280 (34.24)	7.232 (122.14)	–513.3
H_4_pdl/(D_2_O)	3.334 (37.50)	7.461 (121.92)	
**3/**(D_2_O)	3.574 (37.70)	7.552 (123.34)	
**4/**(MeOD)	3.371 (35.51)	7.598 (122.04)	–512.9
H_4_enl/(D_2_O)	3.153 (37.12)	3.430 (40.42)	
**5**/(D_2_O)	3.468 3.357	3.605	
**6**/(D_2_O)	3.177 3.136 (35.29)	3.487 (40.67)	–513.6

a H_1,1_;(C_1,1′_) are the methyl groups of the methylhydroxylamines and H_4_(C_4_) the protons and carbons of the bridge.

The ^1^H NMR spectra of the D_2_O solutions of the vanadate complexes **2**, **4**, and **6** at pDs = 7.0–11.0 exhibit peaks at 3.280 and 7.232 ppm for **2**, at 3.371 and 7.546 ppm for **4**, and at 3.178, 3.137, and 3.486 for **6**, assigned to the hydroxylamine methyl [H(1), H(1′)] and the [H(4)] protons of the bridging ligand, respectively.

The peaks are shifted at lower field vs the respective peaks of the free BLs due to ligation of the BLs to [V^V^O_2_]^+^ structural unit; however, the shift of the peaks of aliphatic protons is 0.2 ppm less than the respective uranyl complexes. The presence of only one symmetric species in solution at various pHs (Figure S11) agrees with the UV–vis spectra.

At this point, it is worth noting that the hydroxylamine methyl groups [H_1_,_1_′__(C_1,1_′__)] of BLs and BLs’ complexes are chemically nonequivalent. However, all compounds except **5** and **6** in the ^1^H NMR spectra give only one signal for both H(1)and H(1′). This is attributed to the fast exchange between the hydroxylamine methyl groups through either rotation of the triazine ring around the C(5)–X bond (X = N or O), when BL is in resonance form A [Scheme S1A(a)], or flip of the triazine ring around atom X when BL is in resonance form B [Scheme S1A(b)]. The 2D EXSY and VT ^1^H NMR spectroscopies^18^ (Figures S13 and S14) reveal an exchange mechanism similar to the inverse umbrella of amines. In the case of the 1,4-phenylenediamine and ethylenediamine complexes **3**–**6**, the exchange mechanism proceeds first through the deprotonation of N(6)–H (Scheme S1C). The N(6)–H proton is more acidic for the 1,4-phenylenediamine than the ethylenediamine complexes, resulting in a faster exchange reaction rate for the former. The fluxional behavior of the complexes is further discussed in the ESI (Scheme S1).

The ^51^V NMR spectra of the vanadate complexes in solution (D_2_O) at pDs 5.0–11.0 (Figure S12) exhibit only one broad peak at −513 ppm, and this fact reveals that the complexes are hydrolytically stable. At pD 11.3, a very small quantity of V^V^O_4_^3–^ (∼5%) is formed. In contrast to uranyl-BL complexes which are hydrolytically stable up to pH 12, the respective vanadate compounds are hydrolyzed above pH 11. The ^51^V NMR chemical shifts of the peaks of **2**, **4**, and **6** are close to the peak of [V^V^O_2_(bihyat)]^−^ (−502 ppm), confirming the formation of the complex with the triazine-hydroxylamino chelate moiety.

### Thermodynamic Stability of Complexes **1–6**

#### Determination of the Stability Constants of V^V^O_4_^3–^ with BLs at pH = 9.0 by UV–Vis and ^1^H NMR Spectroscopies

The solution studies with BLs were not an easy task mainly due to the insolubility of the ligands (Figure S1) and thus were dissolved at high pHs above 12 in their stock solutions. Aliquots of these solutions were used in the NMR and UV–vis experiments at pHs 7–10 and concentrations ∼1 mM. The free ligand in these experiments remained soluble for approximately 24 h.

Stepwise addition of V^V^O_4_^3–^ into the solutions of BLs was monitored by ^1^H NMR spectroscopy and shows the formation of two species, the mononuclear [V^V^O_2_(H_2_BL)]^−^ and the binuclear [(V^V^O_2_)_2_(μ-BL)]^2–^. The mononuclear species are asymmetric and give two sets of peaks for the free and the ligated triazines. For example, H_2_qtn^2–^ and H_2_pdl^2–^ in [V^V^O_2_(H_2_BL)]^−^ shift two aromatic peaks [H(4)] ∼ 0.04 ppm to lower field from the free ligand and ∼0.04 ppm to higher field than the ligand in [(V^V^O_2_)_2_(μ-BL)]^2–^. The stability constants (*K*_2qtn_ = 0.30 ± 0.02, *K*_2pdl_ = 0.30 ± 0.01, and *K*_2enl_ = 0.23 ± 0.01, eq 1) of the equilibrium shown in eq 2 were calculated from the ^1^H NMR spectra of solutions of V^V^O_4_^3–^ and BLs at various concentrations (Figures S15 and S16). The values of *K*_2BL_ show that the BLs with aromatic bridges stabilize more the binuclear vs mononuclear complexes than the BL with the aliphatic bridge, attributed to the interactions through the bridge between the two metal ions in qtn^4–^ and pdl^4–^ ligands.
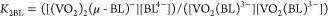
1
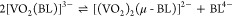
2

Apparently [U^VI^O_2_]^2+^ forms both mononuclear and binuclear species; however, the peaks were too broad due to the formation of uranyl–OH species, and it was not possible to be separated by NMR. The ^1^H NMR measurements show that for both metal ions at concentrations >0.1 mM (BL) and at ratios 2:1 (metal ion:BL) and at the pD range 7–10, the binuclear species exist only in solution.

The β_11_ [log(*K*_11_), eq 3] and β_21_ [log(*K*_21_), eq 4] at pH 9.1 of the equilibria in eqs 5 and 6, respectively were calculated by UV–vis spectroscopy (Figure S17).
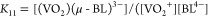
3
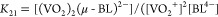
4
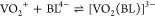
5
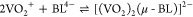
6

The only difference between UV–vis spectra of the aqueous solutions of vanadium complexes and the respective spectra of the ligands is a shoulder at 380–480 nm due to LMCT electron transitions. SQUAD^47^ was fed with the data of spectra of solutions containing various concentrations of vanadate (0.5–2.2 mM) and BL (0.8–1.2 mM) for each BL. The results were satisfactory only for pdl^4–^–VO_4_^3–^ giving the best fit for β_11pdl_ = 8.9 and β_21pdl_ = 17.0. The standard deviation in absorbance data for enl^4–^ and qtn^4–^ was more than 1% mainly due to the error from the poor quality of the absorbance data since the LMCT peak was very close to the absorbance of the ligand. The *K*_2pdl_ (eq 1) calculated from the UV–vis data was smaller (0.18) in comparison to the value with NMR, attributed to the presence of the buffer (Tris) in the solution, which is known to form complexes with vanadate.^48^

### [U^VI^O_2_]^2+^/[V^V^O_2_]^+^, BLs Binding in the Presence of either H_2_dipic or H_2_bihyat or CO_3_^2–^ Monitored by ^1^H NMR Spectroscopy

The stability of the vanadium and uranium–BL complexes was evaluated in the presence of either H_2_dipic or H_2_bihyat and CO_3_^2–^. H_2_dipic has been chosen because it is the strongest aminocarboxylate ligand for uranyl,^49^ while the ligand H_2_bihyat has exceptional strength for both metal ions, surpassing even amidoximes and aminocarboxylate ligands.^13^ The CO_3_^2–^ is a potent uranium ligand and the primary marine uranyl species [U^VI^O_2_^2+^–CO_3_^2–^].

The ^1^H NMR spectra of [VO_2_]^+^ in solution (D_2_O) in the presence of two ligands, either BL/H_2_bihyat or BL/H_2_dipic or BL/CO_3_^2–^, do not show any difference from the spectra of vanadate complexes in D_2_O, at pDs 7.0 and 9.0, even with very high excess of H_2_bihyat/H_2_dipic/CO_3_^–^ (up to [H_2_bihyat]/[BL] = 19, [H_2_dipic]/[BL] = 55, [CO_3_^2–^]/[BL] = 200).

**Figure 3:**
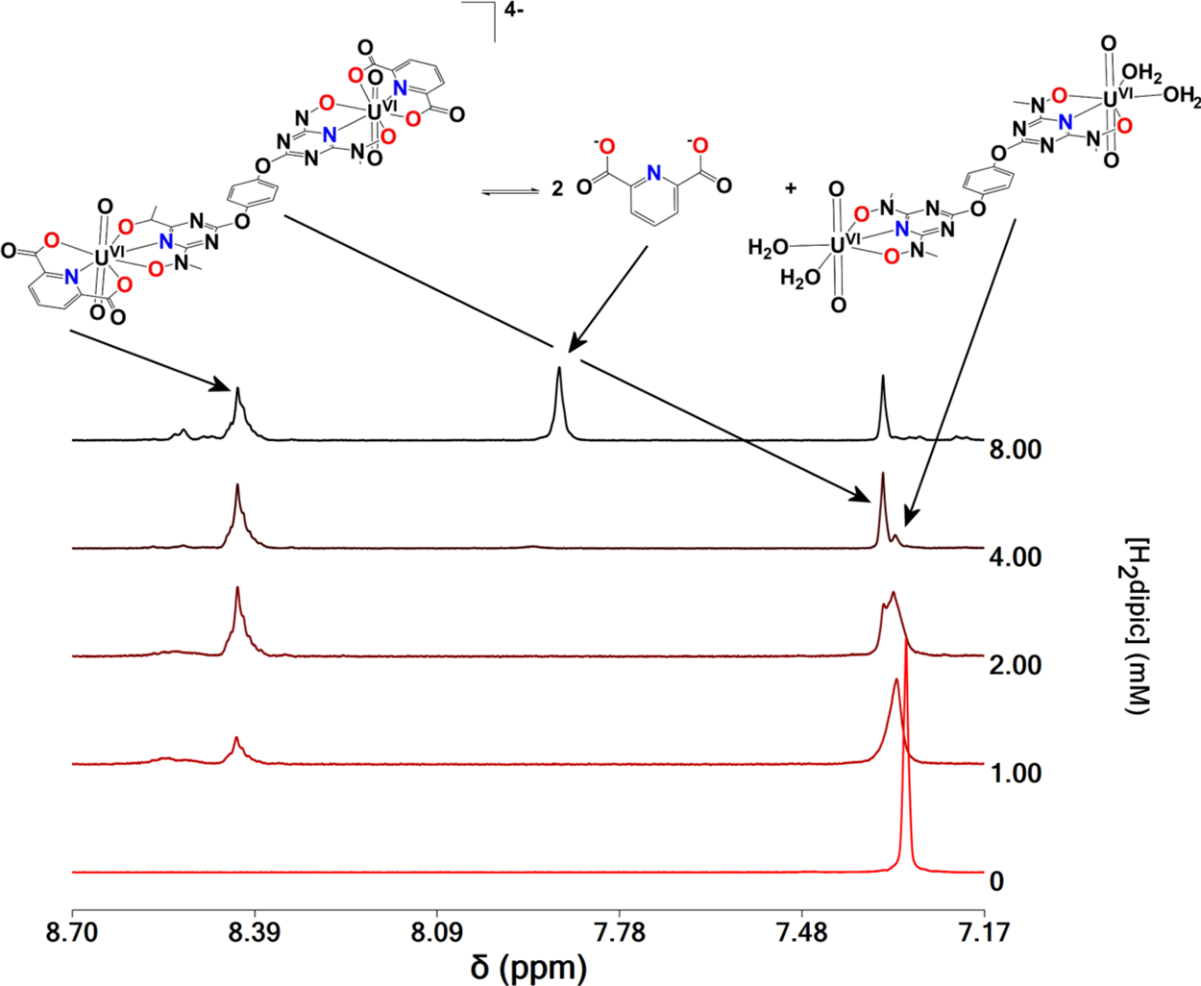
Aromatic region of the ^1^H NMR spectra of D_2_O solutions of **1** (2.00 mM) and H_2_dipic (0–8 mM) at pD = 7.0.

The ^1^H NMR spectra of [UO_2_]^2+^ in solution (D_2_O) in the presence BL/H_2_bihyat, BL/H_2_dipic, and BL/CO_3_^2–^ at pDs 7.0 and 9.0 are shown in Figures 3 and S18–S25. Reaction of the complexes **1**, **3**, and **5** with either H_2_bihyat or H_2_dipic results in the formation of the heteroleptic complexes [(U^VI^O_2_)_2_(μ-qtn)(bihyat)_2_]^4–^ (**7**), [(U^VI^O_2_)_2_(μ-qtn)(dipic)_2_]^4–^ (**8**), [(U^VI^O_2_)_2_(μ-pdl)(dipic)_2_]^4–^ (**9**), and [(U^VI^O_2_)_2_(μ-enl)(dipic)_2_]^4–^ (**10**) (eqs 7 and 8, Scheme 4). Surprisingly complexes **3** and **5** do not form any mixed ligand complex with H_2_bihyat (Figure S22). Presumably, this difference is related to the replacement of the electron-withdrawing hydroquinone oxygen atom in qtn^4^–^^ with the less electron-withdrawing nitrogen atom in pdl^4–^ and enl^4–^, which results in lower basicity for qtn^4–^ than pdl^4–^ and enl^4–^. The formation of [(U^VI^O_2_)_2_(bihyat)_2_]^2–^ and the mixed complexes are favored at high pHs^13^ and probably the high basicity of pdl^4–^ and enl^4–^ does not allow the formation of mixed ligand complexes with bihyat^2–^ for complexes **3** and **5**. The binuclear complexes **7–10** remain stable in solution (D_2_O) even after the addition of high excess of either H_2_bihyat or H_2_dipic (up to [H_2_bihyat]/[BL] = 19, [H_2_dipic]/[BL] = 55).
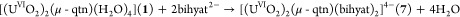
7
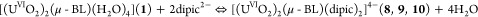
8

Considering that the concentration of H_2_O is constant, it can be included in *K*_dipic(BL)_, and thus *K*_dipic(BL)_ can be calculated from *K*_dipic(BL)_ = [(U^VI^O_2_)_2_(μ-BL)(dipic)_2_^4–^]/([(U^VI^O_2_)_2_(μ-BL)(H_2_O)_4_][ dipic^2–^]^2^).

The chemical shifts of protons H(1) of complex **7** (3.516 ppm) are deshielded after the replacement of the two water molecules of **1** [H(1)3.486 ppm] by bihyat^2–^. The ^1^H NMR peaks of the bound ligand to U^VI^-bihyat^2–^ [H(5) 3.583 ppm and H(6,7) 3.721 ppm] are shifted to lower field toward the peaks of free H_2_bihyat [H(5)3.248 ppm and H(6,7) 3.659 ppm; (Figure S19)] at both pHs 7.0 and 9.0. The H(4) protons of the binuclear complexes **8** [7.336 ppm] and **9** [7.527] are shifted downfield compared with the complexes **1** [7.318 ppm] and **3** [7.321 ppm].

The aliphatic protons H(1,1′) for complexes **8**–**10** and H(4) for **10** give well-defined peaks compared with the broad signals of **1**, **3**, and **5** due to the formation of U^VI^O_2_-(μ-OH)_2_-U^VI^O_2_^4+^-BL^4–^ species and shifted to lower field. It is worth mentioning that the broad peaks of the ^1^H NMR spectra of the uranyl–BL complexes at pDs > 7 after the addition of dipic^2–^ become sharp. This is because the coordination of dipic^2–^ to the uranyl–BL complexes blocks the sites available for the formation of U^VI^O_2_-(μ-OH)_2_-U^VI^O_2_^4+^-BL^4–^ species. Apparently, such species do not form in solution, and the only species are complexes **8**, **9**, or **10** depending on the BL ligand.

The aromatic protons of the free H_2_dipic give peaks at 7.823 ppm (pD = 10.0), 7.886 ppm (pD 9.0), and 7.925 ppm (pD 7.0). The dipic^2–^ ligated to [U^VI^O_2_]^2+^ gives peaks at 8.421, 8.373, and 8.347 ppm for complexes **8**, **9**, and **10**, respectively, at pDs 7–11. The larger the deshielding of the peaks of dipic^2–^ ligated to the metal, the stronger the coordination of [U^VI^O_2_]^2+^ with dipic^2–^. This suggests that the BL electron-donating strength is qtn^4–^< pdl^4–^ < enl^4**^–^**^ with qtn^4–^ being the weaker electron donor of all BLs. The stronger binding of dipic^2–^ in **8** than **9** and **10** is depicted and from the ^1^H NMR calculated equilibrium constants *K*_dipic(BL)_ = [(U^VI^O_2_)_2_(μ-BL)(dipic)_2_^4–^]/([(U^VI^O_2_)_2_(μ-BL)(H_2_O)_4_][ dipic^2–^]^2^) (eq 8). *K*_dipic(qtn)_ = 11 mM^–2^ > *K*_dipic(pdl)_ = 0.66 mM^–2^ > *K*_dipic(enl)_ = 0.35 mM^–2^ suggests that qtn^4–^ is a weaker binder than pdl^4–^ and enl^4–^ for the uranyl moiety.

Addition of excess (up to [CO_3_^2–^]/[BL] = 200) of CO_3_^2–^ into the solutions of **1**, **3**, and **5** in order to remove [U^VI^O_2_]^2+^ from BLs was unsuccessful. The ^1^H NMR spectra of the solutions of **1**, **3**, and **5** became more complicated after the addition of CO_3_^2–^, and the observed features change as a function of [CO_3_^2–^] (Figures S24 and S25). **1**, **3**, and **5** form carbonate complexes, however, without replacing BLs. The broadness and the complication of the peaks of the ^1^H NMR spectra did not allow further investigation of the speciation of the solutions.

Apparently, BLs are extraordinarily strong chelators for both [U^VI^O_2_]^2+^ and [V^V^O_2_]^+^ cations. Taking into account that H_2_bihyat and H_2_dipic are the strongest binders reported so far for both cations,^13,14,50,51^ nonetheless, they cannot remove the metal ions from the binary binuclear complexes **1**–**6**. In addition, BLs remove the metal ions from [U^VI^O_2_(bihyat)(H_2_O)_2_]^+^ and [U^VI^O_2_(dipic)(H_2_O)_2_]^+^ (these complexes were synthesized in situ in solution by mixing the appropriate quantities of the metal ion and the ligand, and their formation was monitored by ^1^H NMR).

#### [V^V^O_2_]^+^ Binding in the Presence of Two BLs

**Figure 4:**
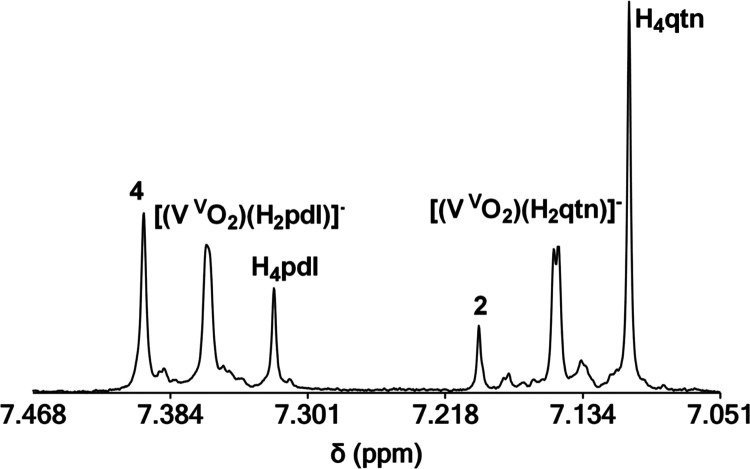
^1^H NMR (aromatic region) of a D_2_O solution containing H_4_qtn (2.00 mM), H_4_pdl (2.00 mM), and V^V^O_3_^4–^ (4.00 mM) at pD = 9.0.

The binding ability of BLs to [V^V^O_2_]^+^ was examined by reacting two BLs with V^V^O_4_^3–^ in D_2_O solutions at pH 7–10. Experiments with [U^VI^O_2_]^2+^ were not examined because of the broadness and complexity of the ^1^H NMR spectra of the uranyl complexes. Addition of excess BLs into solutions of the metal ion results in both 1:1 {[(V^V^O_2_)(BL)]^–^} and 1:2 {[(V^V^O_2_)_2_(μ-BL)]^2–^} complexes, as evident from the ^1^H NMR spectra (Figures 4 and S25). The unreacted free BLs in solution reveal that the binding strength of BLs for V^V^O_4_^3–^ is enl^4–^ > pdl^4–^ > qtn^4–^ with enl^4–^ being the strongest BL chelator. The results are close to the theoretical calculations (enl^4–^ > pdl^4–^∼qtn^4–^) in the negative charge regime (high pH).

#### Binding Selectivity of BLs toward [U^VI^O_2_]^2+^ and [V^V^O_2_]^+^ Cations

**Figure 5:**
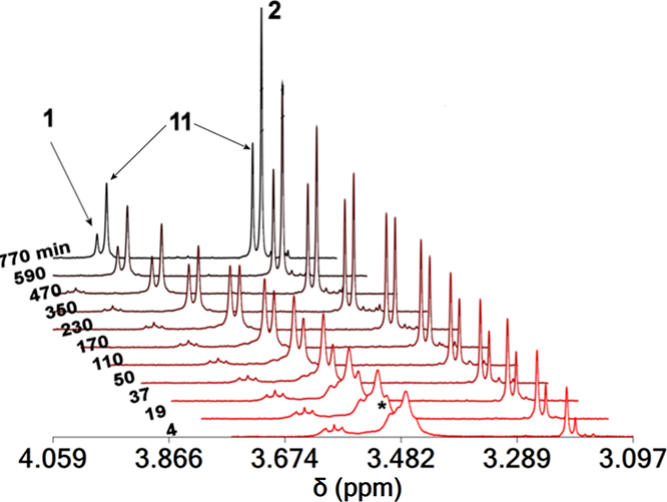
Aliphatic part of the ^1^H NMR spectra of **1** in solution (D_2_O) and V^V^O_4_^3–^ (20 mM) at pD = 9.0 vs time showing the slow formation of **2** and **11**. The signals denoted with the asterisk originated from the H^1^ peaks of U^VI^O_2_-(μ-OH)_2_-U^VI^O_2_^4+^-**qtn**^**4–**^ species.

The ^1^H and ^51^V NMR spectra of the reaction of BLs with various mixtures of [U^VI^O_2_]^2+^ and [V^V^O_2_]^+^ cations at pDs 7.0–10.0 are shown in Figures 5 and S27–S33. The gradual addition of V^V^O_4_^3–^ to a D_2_O solution of complexes **1**, **3**, and **5** at pDs 7.0–10.0, results in the gradual replacement of [U^VI^O_2_]^2+^ by the [V^V^O_2_]^+^ moiety and the formation of the heterometallic complexes [(U^VI^O_2_)(V^V^O_2_)(μ-BL)(H_2_O)_2_]^−^ (**11**, **12**, and **13**Scheme 4) and complexes **2**, **4**, and **6**, respectively, accorsing toeqs 9 and 10. Similar results were obtained from the addition of [U^VI^O_2_]^2+^ into a D_2_O solution of the binuclear complex **2**. The rate of the forward reaction (Figure S33), replacement of [U^VI^O_2_]^2+^ by [V^V^O_2_]^+^, is much slower than the backward reaction, replacement of [V^V^O_2_]^+^ by [U^VI^O_2_]^2+^ (Figure S34). Thus, in order to reach equilibrium, the samples were heated prior to each measurement. The free [U^VI^O_2_]^2+^ in the solution precipitates out as a yellow hydroxide salt [U^VI^O_2_(OH)_2_]. In the presence of vanadate, [U^VI^O_2_]^2+^ precipitates as a mixed V^V^–U^VI^ hydroxide yellow salt with the metal ions in the 1:1 ratio.
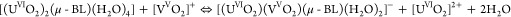
9
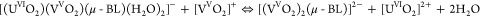
10

The slow rate of [V^V^O_2_]^+^ reaction with **1**, **3**, and **5** might be attributed to a mechanism in which the U^VI^-μ-OH-U^VI^ bonds break down toward the formation of U^VI^- μ-O-V^V^ oxometallates, and then [V^V^O_2_]^+^ replaces the U^VI^–O–V^V^ moieties. The suggested mechanism is also supported from the fast rates of substitution of [U^VI^O_2_]^2+^ from [V^V^O_2_]^+^ in **1**, **3**, and **5** at lower pDs (7.0), whereas the formation of U^VI^–O-V^V^ cluster is less likely. In addition, the fast rates of the reverse reactions of **2**, **4**, and **6** with [U^VI^O_2_]^2+^ to give **1**, **3**, **5**, and **11–13** are also evident for the mechanism because **2** does not form V^V^- μ-O-V^V^-**qtn**^**4–**^ molecules, and therefore, the coordination of [U^VI^O_2_]^2+^ is not inhibited.

The ^1^H NMR spectra of the mixed-metal asymmetric complexes **11** and **12** in D_2_O show two peaks for the CH_3_–N–O^–^ moieties at 3.497, 3.255, and 3.584, 3.221 ppm, respectively (Figures S35–S38). In addition, **11** gave two doublets for the hydroquinone protons (7.310, 7.256 ppm and *J*_4–5_ = 7.6 Hz, Figure 5), and **12** gave two broad peaks at 7.468 and 7.408 ppm. 2D^18^ grCOSY has been used to identify the coupling between the aromatic protons (Figure S28). The ^1^H NMR spectra of complex **13** gave two peaks at 3.083 and 3.043 assigned to the methyl groups of hydroxylamines.

After the addition of V^V^O_3_^–^ into the solutions of **1**, **3**, or **5**, a yellow precipitate was formed. The ^51^V NMR spectra of the solutions did not show any signal attributable to the V^V^O_4_^3–^ anion even with an excess of vanadate in solution, indicating that U^IV^O_2_^2+^ coprecipitates with V^V^O_4_^3–^ as a 1:1 salt. At this point, it is worth noting that when excess of V^V^O_4_^3–^ is added in the aqueous solution, U^VI^O_2_^2+^-bihyat results in the formation of the heterobimetallic [(U^VI^O_2_)_3_(V^V^O_4_)_2_(H_2_bihyat)_2_] compound in addition to [U^VI^O_2_(bihyat)(H_2_O)_2_].^13^

Integration of the ^1^H NMR peaks of each species gave for the solutions containing 2 mM (BL):4 mM (V^V^O_4_^3–^):4 mM (U^IV^O_2_^2+^) at pD = 10 the following speciation: (a) for H_4_qtn: 39% (**2**): 39% (**11**): 22% (**1**), (b) for H_4_pdl: 64% (**4**): 26% (**12**): 10% (**3**) and (c) for H_4_enl: 83% (**6**): 17% (**13**): 0% (**5**). The ligands become better binders for uranyl at pDs > 10. The results show that BLs are stronger vanadium binders than uranium in agreement with the theoretical calculations. H_4_enl is the stronger vanadium binder, whereas H_4_pdl shows preference for uranyl at pHs > 10.

The decrease of the selectivity of BLs vs bihyat^2–^ to bind [U^VI^O_2_]^2+^ might be attributed to the difficulty, defined by the binuclear geometry of U–BL complexes, to acquire [U^VI^O_2_(BHT)_2_]^2–^-type coordination in solution. However, as shown in this study, the equilibrium between either the binucleating or mononucleating ligands in solutions containing both V^V^O_4_^3–^ and [U^VI^O_2_]^2+^ is very complex. This has to do with the generation of various bimetallic V^V^O_4_^3–^–U^VI^O_2_^2+^ inorganic species that might be also responsible for the selectivity.

The mononucleating ligand H_2_bihyat at alkaline pDs >7 forms [U^VI^O_2_(bihyat)_2_]^2–^, significantly increasing the affinity and selectivity of the ligand toward [U^VI^O_2_]^2+^. In addition, the larger negative charge of [(V^V^O_2_)_2_(μ-BL)]^2–^ (**2**) than [(V^V^O_2_)(bihyat)]^−^ might offer an extra stabilization for the binuclear vanadate complexes, through a better solvation, than the neutral [(U^VI^O_2_)_2_(μ-BL)(H_2_O)_4_], thus diminishing the preference of the binucleating BHT ligand for [U^VI^O_2_]^2+^ binding.

**Scheme 6:**
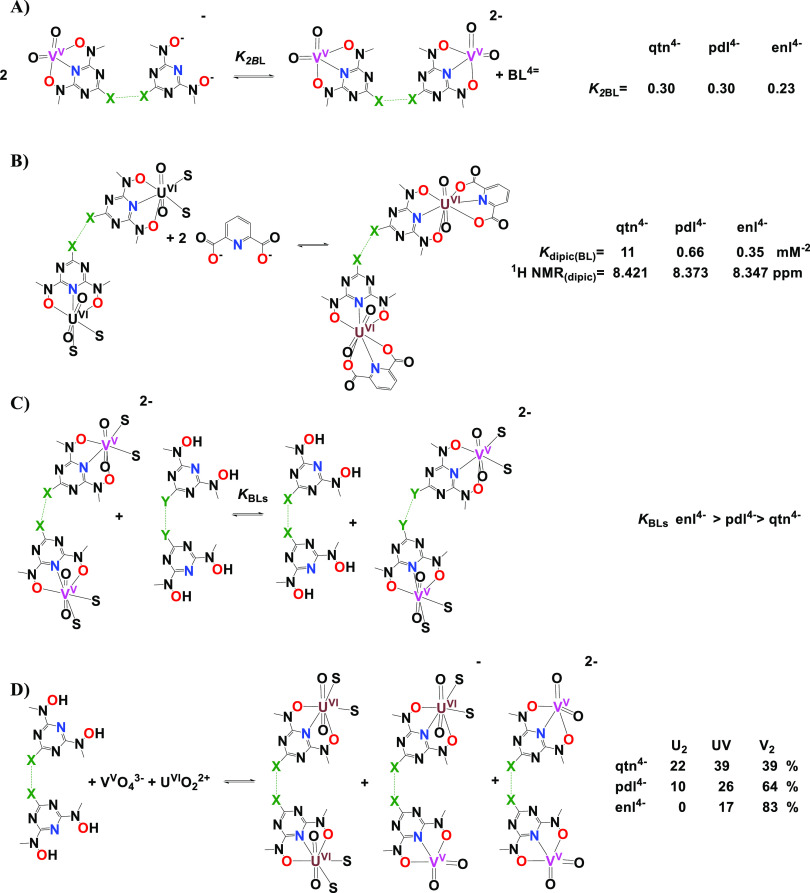
Summary of the Thermodynamic Stability of the V^V^ and U^VI^ Complexes on the Basis of ^1^H NMR Spectroscopy, (A) V^V^O_4_^3–^-BLs Titration, (B) Competition Studies of BLs and H_2_dipic for [U^VI^O_2_]^2+^ Binding. (C) [V^V^O_2_]^+^ Binding in the Presence of two BLs. (D) Binding Selectivity of BLs toward V^V^O_4_^3–^ and [U^VI^O_2_]^2+^; the % is the Percentage of the Three Complexes Formed in Solution

The results of the ^1^H NMR stability studies are summarized in Scheme 6.

#### ESI-MS

The ESI-MS studies of the solutions of BLs with uranyl or vanadate at pH = 9.0 are shown in Figure S45. The MS spectra show peaks assigned mainly to the bimetallic species. The MS spectra of the uranyl show the presence of a larger number of species including clusters of higher molecular weight than the vanadate–BL solutions.

The U^VI^O_2_^2+^-qtn^4–^ solutions after the addition of H_2_bihyat show formation of species **6** (Scheme 4 and Figure S46A). In contrast, U^VI^O_2_^2+^-pdl^4–^/enl^4–^ did not give any heteroleptic complexes (Figure S46B) in agreement with the ^1^H NMR (supra infra). Aqueous solutions of U^VI^O_2_^2+^-qtn^4–^/pdl^4–^/enl^4–^ with H_2_dipic show peaks from species **8**–**10** (Figure S46C,D).

MS spectra of the solutions of BLs in the presence of both [U^VI^O_2_]^2+^ and [V^V^O_2_]^+^ gave peaks of the heterobimetallic U^VI^O_2_^2+^–V^V^O_2_^+^–BL complexes **11–13**, supporting their formation as suggested by ^1^H NMR spectroscopy (Figure 5).

ESI-MS provided a unique opportunity in this work not only to identify^52−57^ and confirm the structural stability of the species in the reaction mixture as a function of the pH value but also allowed us to monitor the occurred speciation and selectivity of the designed ligands against the heavy metals under investigation. The observed change of the oxidation state of the metal centers in some cases is due to the ionization and consecutive ion-transfer process of the charged species and has been observed previously in numerous occasions.^52−57^ Additionally, it allowed us to sharpen the data obtained from the NMR studies discussed above and draw safer conclusions following this cooperative study. Initially we investigated the behavior of the reaction mixture using either H_4_qtn or H_4_enl in the presence of a single transition metal (either [U^VI^O_2_]^2+^ or [V^V^O_2_]^+^) under identical experimental conditions. Figure S45 demonstrates the ability of both ligands to coordinate with the transition metals of interest forming bimetallic species. In the case of H_4_qtn and [U^VI^O_2_]^2+^, we observed doubly charged characteristic isotopic envelopes located in the region of 1000–1100 *m*/*z* values which can be assigned to two bimetallic moieties {(U^V^_2_O_10_N_10_C_16_H_16_)_2_(H_2_O)_5_}^2–^ located at 1029.1 *m*/*z* flanked by a series of envelopes attributed to the same moiety with varying combinations of solvent molecules. In the case of [V^V^O_2_]^+^, we observed again a vanadium-based bimetallic species with a relevant distribution envelope centered at 949.0 *m*/*z* and can be assigned to {(V^V^_2_O_10_N_10_C_16_H_16_)(Ph_4_P)}^−^. Interestingly in the case of H_4_enl and [U^VI^O_2_]^2+^ and due to the flexibility of the ligand, we observed doubly charged bimetallic species in the region of 450–600 *m*/*z* but also a tetrametallic triply charged [U^VI^O_2_]^2+^ species located at 657.1 *m*/*z* with a formula of {(U_2_O_8_N_12_C_12_H_18_)_2_(CH_3_OH)(OH_2_)_3_OH}^3–^.

The second part of our study involved the investigation of the competitive nature of ligands for [U^VI^O_2_]^2+^ metal centers based on their known coordination abilities (Figure S46). More specifically, we explored the mixtures of H_4_qtn/H_2_bihyat, H_4_pdl/H_2_bihyat, H_4_qtn/H_2_dipic, and H_4_enl/H_2_dipic all in 1:2 ratios in the presence of 2 equiv of [U^VI^O_2_]^2+^. In every case, the ditopic ligands H_4_qtn, H_4_pdl, and H_4_enl exhibited their efficacy for coordination by “capturing” in every case two [U^VI^O_2_]^2+^ centers. In a competitive chemical environment of H_4_qtn and H_2_bihyat, the majority of the species appear to be bimetallic and monometallic complexes of H_4_qtn with their distribution envelopes centered at 715.1, 779.2, and 828.3 *m*/*z* and to a lesser extent H_4_qtn:H_2_bihyat 1:1 moiety (644.1 *m*/*z*). Interestingly, in the case of H_4_pdl/H_2_bihyat couple, only monometallic [U^VI^O_2_]^2+^ species of H_4_pdl have been identified with the relevant singly charged distribution envelope centered at 713.1 *m*/*z*. Moving on to the last two cases of H_4_qtn/H_2_dipic and H_4_enl/H_2_dipic, the increased coordination ability of H_2_dipic becomes apparent. In both cases, we were able to identify bimetallic [U^VI^O_2_]^2+^ species of 1:1 as well as 1:2 ratios of H_4_qtn/H_2_dipic and H_4_enl/H_2_dipic ratios with the relevant doubly charged distribution envelopes centered at 658.1, 677.0, and 695.0 *m*/*z* and 633.1, 638.9, 651.5, and 670.9 *m*/*z* values for the two cases of mixed ligand systems, respectively.

Finally, we embarked on an effort to explore the behavior of the ditopic ligands in the competitive coordination environment of [U^VI^O_2_]^2+^ and [V^V^O_2_]^+^. In the case of the more rigid ligand H_4_qtn, we observed a range of doubly charged bimetallic [U^VI^O_2_]^2+^ or [V^V^O_2_]^+^ species with their relevant envelopes centered at 304.0, 609.0, and 515.1 *m*/*z* values with formulas {(V^IV^_2_O_8_N_12_C_16_H_18_)}^2–^, {(V^IV^_2_O_8_N_12_C_16_H_18_)H}^−^ {(U^VI^U^V^O_9_N_12_C_16_H_18_)(CH_3_OH)}^2–^, as well as mixed-metal bimetallic [U^VI^O_2_]^2+^/[V^V^O_2_]^+^, with their distribution envelopes centered at 406.0 and 795.1 *m*/*z* attributed to {(U^VI^V^IV^O_8_N_12_C_16_H_18_)OH}^2–^ and {(U^VI^V^IV^O_8_N_12_C_16_H_18_)}^−^, respectively, as shown in Figure S47A. Interestingly, in the case of the more flexible ditopic **H**_**4**_**enl** ligand, there was a preference toward the formation of singly charged bimetallic [V^V^O_2_]^+^ species centered at 561.0, 582.9, and 598.9 *m*/*z* attributed to {(V^V^_2_O_8_N_12_C_12_H_18_)H}^−^, {(V^III^_2_O_8_N_12_C_12_H_18_)H_5_(H_2_O)}^−^, and {(V^IV^V^V^O_8_N_12_C_12_H_18_)H_3_(H_2_O)_2_}^−^, respectively, even though a small trace of also singly charged [U^VI^O_2_]^2+^/[V^V^O_2_]^+^ mixed-metal bimetallic moiety {(U^V^V^V^O_8_N_12_C_12_H_18_)}^−^ has been detected at 747.1 *m*/*z*.

## Computational Results

To assess the complexation stability of the four ligands (bihyat^2–^, qtn^4–^, enl^4–^, and pdl^4–^) to uranyl and vanadate, a computational survey concerning the complexation reactions of all ligands was carried out (see Computational Methods section). Geometry optimizations were performed on the obtained crystal structure coordinates, and in their absence, the atoms were edited to obtain the respective isomer.

The main challenge is to find a suitable reference state reactant in aqueous solution. The problem is further compounded by the pH dependence of the process. Since the pH values of the reactions are close to the neutral range, both acidic and alkaline reactions will be considered.

From the literature data, namely, Cruywagen’s review,^58^ it is reasonable to assume that at high concentrations of H^+^, the dominant species of vanadium(V) will be [V^V^O_2_]^+^, whereas in alkaline solution, it will be dihydrogen vanadate H_2_V^V^O_4_^–^. For the case of uranyl, there is a speciation study by Panias and Krestou^59^ that establishes the predominance of [U^VI^O_2_]^2+^ in acidic conditions and [U^VI^O_2_(OH)_3_]^−^ in basic media.

**Figure 6:**
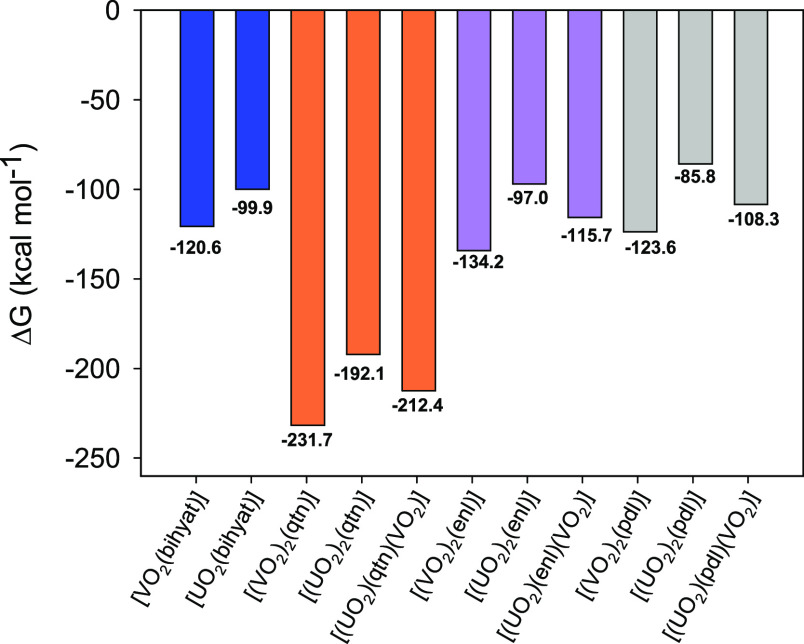
DFT:rev-PBE-D4/TZP(DZP) calculated free energies of formation of the several possible complexes in acidic solution. Blue: bihyat^2–^, orange: qtn^4–^, violet: enl^4–^, and gray: pdl^4–^.

The following complexation free energies of a set of test reactions were computed:
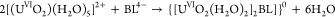
11
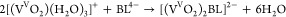
12

where L = qtn^4–^, enl^4–^, and pdl^4–^. These will be representative of a proton rich medium. For the bihyat^2^–^^ ligand, the simpler monometallic complexation will take place with three water molecules leaving. These results are summarized in Figure 6 below.

It should be noted that the overall absolute values in Figure 6 are likely to be inflated since the bihyat^2–^, qtn^4–^, enl^4–^, and pdl^4–^ ligands will likely be partially protonated in solution.

These numbers, however, allow us to draw some interesting trends. Throughout the spectrum of ligands, there is a consistent preference for the complexation with vanadate. The mixed {[U^VI^O_2_(H_2_O)_2_]BL(V^V^O_2_)}^−^ complexes have their energy lie in between [(V^V^O_2_)_2_BL]^2–^ and {[U^V^O_2_(H_2_O)_2_)]_2_BL} dimers.

The qtn^4–^ ligand stands out as having the most affinity for the metal oxido units.

In order to assess the alkaline solution regime, the next series of complexation free energies were computed in accordance with the reaction schemes:
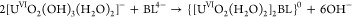
13
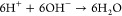
14
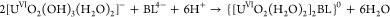
15

For the uranyl species, since the hydroxide anion is a poor leaving group, reaction 3 will be endergonic. It was decided therefore to weigh this reaction against the autoionization of water so that the more stable water molecule could be the leaving group.

The same was carried out for vanadate with a slightly different variation, i.e.,
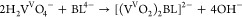
16
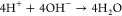
17
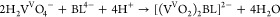
18

and for the mixed species
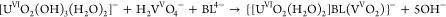
19
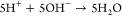
20
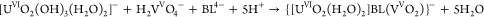
21

For the bihyat^2–^ ligand, the following monometallic reactions were tested:
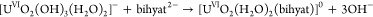
22
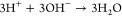
23
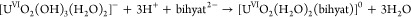
24
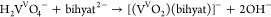
25
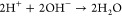
26
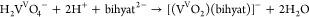
27

**Figure 7:**
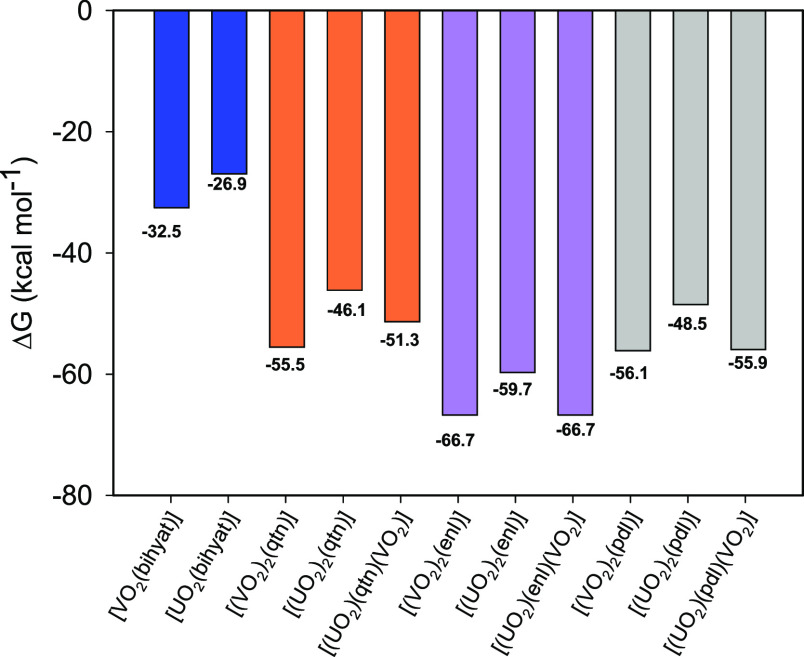
DFT:rev-PBE-D4/TZP(DZP) calculated free energies of formation of the several possible complexes in alkaline solution. Blue: bihyat^2–^, orange: qtn^4–^, violet: enl^4–^, and gray: pdl^4–^.

The figures of these reactions are depicted in Figure 7. It may be seen that the anionic oxido species afford a different stability scenario.

Within each class of ligand, the complexation energies of dihydrogen vanadate and trihydroxouranyl are much closer, almost to the point of indistinguishability. In the case of enl^4–^ ,for example, the complexation energies are exactly the same, i.e., −66.7 kcal mol^–1^. The most contrasting values in the bimetallic class are with the qtn^4–^ ligand where the homometallic dimers are 4 kcal mol^–1^ apart. In the case of bihyat^2–^, the difference is 5.5 kcal mol^–1^ between H_2_V^V^O_4_^–^ and [U^VI^O_2_(OH_3_)]^−^. The calculations reveal that the V^V^-bihyat^2–^ complex is more stable than U^VI^-bihyat^2–^ in contrast to the experimental results.^13^ However, in the theoretical studies, the presence of the U^VI^ species with two bihyat^2–^ {[U^VI^O_2_(bihyat)_2_]^2–^} and the −OH bridged binuclear U^VI^-bihyat^2–^ have not been considered. Nevertheless, the theoretical studies show that the ligation of the mononucleating ligand with V^V^ or U^VI^ is much weaker than BLs, in agreement with the experiment.

A two-fragment molecular orbital (FMO) analysis was also performed on the {[U^VI^O_2_(H_2_O)_2_]_2_BL} species in order to quantify the electron donation from the anionic ligands to the metal sites. The two fragments were the two [U^VI^O_2_(H_2_O)_2_]^2+^ moieties plus the BL^4–^ ligands qtn^4–^, pdl^4^–^^, and enl^4–^. The FMO Mulliken populations are −0.277, −0.300, and −0.315, the minus sign signifying that the electron transfer goes from the ligand fragment BL^4–^ to the two-site cationic fragment. This is consistent with the NMR results which demonstrated the ^1^H chemical shift changing in the same direction (Figure S39).

## Conclusions

The binuclear complexes [(U^VI^O_2_)_2_(BL)(H_2_O)_4_] and [(V^V^O_2_)_2_(BL)]^2–^ with the novel hydroxylamino-triazine binucleating ligands H_4_qtn, H_4_pdl, and H_4_enl (BLs) were synthesized and structurally and physicochemically characterized. The X-ray structure analysis of both metal ions with BLs reveals an extraordinary strong binding of BLs to [U^VI^O_2_]^2+^ and [V^V^O_2_]^+^. The ligand BLs used in this study are much stronger chelators than the amidoximes that are currently utilized for uranyl mining from the sea, according to competing investigations of the BLs with ligands like H_2_dipic and H_2_bihyat. This is attributed to the negative formal charge of the triazine nitrogen atom and the deprotonated hydroxylamine oxygen donor atoms. The UV–vis and ^1^H and ^51^V NMR spectra of the aqueous solutions of binuclear complexes **1–6** confirm the strong binding properties of BLs to [U^VI^O_2_]^2+^ and [V^V^O_2_]^+^ and also reveal the high thermodynamic stability of the complexes in a large pD range, 7–12. The bridging moiety of the ligands influences the stability of the complexes, with the ligands exhibiting aromatic bridges, qtn^4–^ and pdl^4–^, to form less stable complexes than the aliphatic, enl^4–^. This is attributed to the better delocalization of the negative triazine charge in the aromatic rings than the aliphatic chain and, thus, lower basicity for the chelating moieties. ESI-MS and ^1^H NMR have shown that uranyl complexes in solution at alkaline pH form U^VI^–OH–U^VI^ polymeric species.

Reactions of either H_2_dipic or H_2_bihyat with the uranyl complexes **1**, **3**, and **5** result in the formation of heteroleptic binuclear complexes **7–10** as evident from ^1^H NMR spectroscopy and ESI-MS. In the equatorial plane of the binuclear complex **7**, the ligands qtn^4–^-bihyat^2–^ and in complexes **8**–**10,** the ligands BLs-dipic^2–^ coexist. Complexes **3** and **5** do not react with H_2_bihyat to form the heteroleptic binuclear uranyl complexes, and this is attributed to the strong electron donor properties of pdl^4–^/enl^4–^ that do not allow the coordination of a strong donating ligand (bihyat^2–^) in a trans position. This finding suggests that two triazine-hydroxylaminate chelate groups from the two strong donating BLs cannot occupy the equatorial plane of [U^VI^O_2_]^2+^, supporting the possibility that this type of chelation is the basis for bihyat^2–^’s preference for [U^VI^O_2_]^2+^ over [V^V^O_2_]^+^. The calculated net interfragment electron donations of the BLs to [U^VI^O_2_]^2+^ is linearly dependent on the ^1^H NMR chemical shift of the protons of dipic^2–^ lying at the equatorial plane of complexes **8**–**10**, supporting that qtn^4–^ is a weaker electron donor than pdl^4–^/ enl^4–^. High excess up to saturation in water of either H_2_dipic or H_2_bihyat or CO_3_^2–^ does not replace BLs^–^ from complexes **1–6.** Considering that H_2_bihyat is one of the strongest ligands for [U^VI^O_2_]^2+^ and [V^V^O_2_]^+^, the BLs form uranium(VI) and vanadium(V) complexes that are even more thermodynamically stable than H_2_bihyat, which was also confirmed by theory. Reactions of uranyl with the vanadate complexes **2**, **4**, and **6** result in the formation of the homometallic **1**–**6** and heterometallic **11**–**13** binuclear complexes as evident from ^1^H NMR spectroscopy and ESI-MS. The reverse reaction, i.e., addition of vanadate to uranyl complexes **1**, **3**, and **5**, is a much slower reaction than the addition of uranyl to vanadate complexes because of the formation of the U^VI^–OH–U^VI^–BL polymeric species. Although the thermodynamic stability of [U^VI^O_2_]^2+^/BLs and [V^V^O_2_]^+^/BLs^–^ has increased significantly compared to bihyat^2–^, the BLs are less selective for [U^VI^O_2_]^2+^ over [V^V^O_2_]^+^ than bihyat^2^ since the most stable complexes in solution are [(V^V^O_2_)_2_(μ-BL)]^2–^. From the three binucleating ligands, enl^4–^ forms the most stable hydrolytically metal complexes as predicted from the theoretical calculations.

In conclusion, the binucleating BHT-type siderophores, such as BLs used in this study, exhibit exceptional thermodynamic stability for hard metal ions; thus, they are potentially suitable for biodetoxification and hard metal separation technologies. In order to increase selectivity for [U^VI^O_2_]^2+^ over [V^V^O_2_]^+^, new chelators have to be designed forcing the coordination of two triazine chelating groups at the equatorial plane of the metal ion. However, as depicted in this work, the electron-donating properties of the two chelators at the equatorial plane of [U^VI^O_2_]^2+^ should be judiciously chosen, thus minimizing the competition between the groups to ligate uranyl.

## Supporting Information Available

Electronic Supporting Information (ESI) available: For ESI and crystallographic data in CIF for other electronic format see DOI:
Solubility of H_4_qtn *vs* pH; IR spectra; variable pH UV spectra; ^1^H NMR spectra; VT ^1^H NMR spectra; calculation of *K*_2pdl_ from ^1^H NMR; speciation diagram based on the spectroscopic titration of 1.000 mM H_4_pdl and addition of various quantities of [VO_4_^3–^] at pH 9.1; aromatic and aliphatic parts of the ^1^H NMR spectra; ESI-MS spectra; crystal data and structure refinement for compounds **1**, **2**, **3**, **4**, and **6**; interatomic distances (Å) and angles (deg) relevant to the U^VI^ and V^V^ coordination sphere; and DFT-optimized structures CCDC: 2211326 for **1**, 2211327 for **2**, 2284962 for **3**, 2284963 for **4** and 2284964 for **6** A data set collection^60^ of the optimized structures is available in the ioChem-BD repository^61^ (PDF)
