# Supplementary material for: Design and Modulation of Selectivity toward Vanadium(V) and Uranium(VI) Ions: Coordination Properties and Affinity of Hydroxylamino-Triazine Siderophores
Source: Inorg Chem. 2023 Nov 29;62(49):19971–85. doi: 10.1021/acs.inorgchem.3c02678 (PMC10716903; doi:10.1021/acs.inorgchem.3c02678)
Supplement: Supplementary file 1 — ic3c02678_si_001.pdf [file ic3c02678_si_001.pdf]

Supporting Information

Design and Modulation of Selectivity toward Vanadium(V) and Uranium(VI) Ions: Coordination Properties and Affinity of Hydroxylamino-Triazine Siderophores

Angelos Amoiridis,[a] *Michael Papanikolaou,*[a] *Manolis Vlasiou,*[b] *Nuno A. G. Bandeira,*[*c] *Haralampos N. Miras,*[*d] *Themistoklis Kabanos*[*e] *and Anastasios Keramidas*[*a]

[a] *University of Cyprus, Department of Chemistry, 2109, Nicosia, Cyprus. Email:* akeramid@ucy.ac.cy

[b] University *of Nicosia, School of Veterinary Medicine, 2414, Nicosia, Cyprus.*

[c] *Biosystems and Integrative Sciences Institute (BioISI)- Departamento de Química e Bioquímica. Faculdade de Ciências Universidade de Lisboa; 8.5.53 - C8 Campo Grande, 1749-016 Lisboa Portugal. E-mail:* nuno.bandeira@ciencias.ulisboa.pt

[d] *School of Chemistry, The University of Glasgow. Glasgow G12 8QQ (UK). Email:* Charalampos.moiras@glasgow.ac.uk

[c] *Department of Chemistry, Section of Inorganic and Analytical Chemistry, University of Ioannina, 45110 Ioannina, Greece. Email:* tkampano@uoi.gr

Contents Pages

Experimental **S6**

Figure S1. Solubility of H_4qtn *vs* pH. Based on ^{1}H NMR and using CH_3COONa as internal standard for quantification. **S13**

Figure S2. IR spectrum of qtCl_4, H_4qtn, **1** and **2**. **S14**

Figure S3. IR spectrum of H_4pdl, **3** and **4**. **S15**

Figure S4. IR spectrum of H_4enl, **5** and **6**. **S16**

Figure S5. Variable pH UV-spectra of A) **1** (1 mM), B) **2** (0.75 mM) and C) qtn^{4-} (0.05 mM) at pH 11.8. **S17**

Figure S6. Variable pH UV-spectra of A) **3** (0.125 mM, pHs 7-10), B) **4** (0.125 mM, pHs 7-10) and C) pdl^{4-} (0.100 mM) at pH 12. **S18**

Figure S7. Variable pH UV-spectra of A) **5** (2.00 mM, pHs 7-10), B) **6** (0.500 mM, pHs 7-10) and C) enl^{4-} (0.125 mM) at pH 12. **S19**

Figure S8. ^{1}H NMR spectra of **1** in solution (D_2O) (2.00 mM) at various pDs. The signals denoted with the asterisk are due to the H(1) peaks of $U^{VI}O_2$-(μ-OH)$_2$-$U^{VI}O_2^{4+}$-enl^{4-} species. **S20**

Figure S9. ^{1}H NMR spectra of **3** in solution (D_2O) (2.00 mM) at various pDs. The signals denoted with the asterisk are due to the H(1) peaks of $U^{VI}O_2$-(μ-OH)$_2$-$U^{VI}O_2^{4+}$-enl^{4-} species. **S21**

Figure S10. ^{1}H NMR spectra of **5** in solution (D_2O) (2.00 mM) at various pDs. The signals denoted with the asterisk are due to the H(1) peaks of $U^{VI}O_2$-(μ-OH)$_2$-$U^{VI}O_2^{4+}$-enl^{4-} species. **S22**

Figure S11. ^{1}H NMR spectra of D_2O solutions of **2** (2.00 mM) at various pDs. **S23**

Figure S12. ^{51}V NMR spectra of D_2O solutions of **2** (2.00 mM) at various pDs. V_1 is VO_4^{3-}. **S24**

Figure S13. A) VT ^{1}H NMR spectra of **6** in solution (D_2O) (2.00 mM) at pH=10, B) 2D **{**^{1}H} EXSY NMR spectra of **6** in solution (D_2O) (2.00 mM) at pH=10 and 20 °C. **S25**

Figure S14. A) VT ^{1}H NMR spectra, experimental and simulated, of **6** in solution (D_2O) (2.00 mM) at pH=10, B) Eyring plot ln(k/T) *vs* 1/T, $\Delta S^{\#}$=197 J/mol, $\Delta H^{\#}$=127 kJ/mol/K. **S26**

Figure S15. ^{1}H NMR spectra of the titration of Hpdl [in solution (D_2O), 2.00 mM] by VO_4^{3-} (0-4.50 mM) at pD = 9.0. **S27**

Figure S16. a) Diagram for the calculation of K_{2pdl} from the ^{1}H NMR of Figure S15, b) speciation diagram considering 1.00 mM of the total concentration of [VO_4^{3-}]. **S28**

Figure S17. a) Speciation diagram based on the spectroscopic titration of 1.000 mM H_4pdl and addition of various quantities of [VO_4^{3-}] at pH 9.1. The points are the experimental concentrations used for the titration experiment. b) Predicted spectra for [V^VO_2(pdl)]$^{-1}$ and the experimental for [(V^VO_2)$_2$(pdl)]$^{2-}$. **S29**

Figure S18. ^{1}H NMR spectra of D_2O solution of $U^{VI}O_2^{2+}$ (2.00 mM), H_2qtn (4.00 mM) and H_2bihyat (0 – 8 mM) at pD = 9.0. **S30**

Figure S19. ^{1}H NMR spectra of *trans*-[$U^{VI}O_2$]$^{2+}$ [in solution (D_2O), 2.00 mM], H_4qtn (4.00 mM) and H_2bihyat (0 – 8 mM) at pD = 7.0. **S31**

Figure S20. ^{1}H NMR spectra of *trans*-$[U^{VI}O_2]^{2+}$ [in solution (D_2O), 2.00 mM], H_4qtn (4.00 mM) and H_2dipic (0 – 8 mM) at pD = 7.0. **S32**
Figure S21. ^{1}H NMR spectra of *trans*-$[U^{VI}O_2]^{2+}$ [in solution (D_2O), 2.00 mM], H_4pdl (4.00 mM) and H_2dipic (0 – 40 mM) at pD = 10.0. **S33**
Figure S22. ^{1}H NMR spectra of *trans*-$[U^{VI}O_2]^{2+}$ [in solution (D_2O), 2.00 mM], H_4pdl (4.00 mM) and H_2bihyat (0 – 20 mM) at pD = 9.0. **S34**
Figure S23. ^{1}H NMR spectra of *trans*-$[U^{VI}O_2]^{2+}$ [in solution (D_2O), 2.00 mM], H_4enl (4.00 mM) and H_2dipic (0 – 20 mM) at pD = 10.0. **S35**
Figure S24. ^{1}H NMR spectra of *trans*-$[U^{VI}O_2]^{2+}$ [in solution (D_2O), 2.00 mM], H_4pdl (4.00 mM) and Na_2CO_3 (0 – 400 mM) at pD = 9.0. **S36**
Figure S25. ^{1}H NMR spectra of *trans*-$[U^{VI}O_2]^{2+}$ [in solution (D_2O), 2.00 mM], H_4enl (4.00 mM) and Na_2CO_3 (0 – 400 mM) at pD = 9.0. **S37**
Figure S26. ^{1}H NMR (aromatic region) of D_2O solution containing H_4enl, H_4pdl and VO_3^{4-} at various concentration at pD=9.0. **S38**
Figure S27. ^{51}V NMR spectra of D_2O solution of $U^{VI}O_2^{2+}$ (2.00 mM), H_2qtn (4.00 mM) and VO_4^{3-} (0 – 20 mM) at pD = 7.0. **S39**
Figure S28. 2D {^{1}H}COSY of the aromatic part of the ^{1}H NMR spectra of D_2O solution of **1** and VO_4^{3-} (20 mM) at pD = 9.0. **S40**
Figure S29. Aromatic part of the ^{1}H NMR spectra of D_2O solution of **1** and VO_4^{3-} (20 mM) at pD = 9.0 *vs* time. **S41**
Figure S30. Aliphatic part of the ^{1}H NMR spectra of D_2O solution of **1** and VO_4^{3-} (20 mM) at pD = 7.0 *vs* time. The signals denoted with the asterisk are originated from the H(1) peaks of $U^{VI}O_2$-(μ-OH)$_2$-$U^{VI}O_2^{4+}$-qtn^{4-} species. Numbering according to Scheme 3. **S42**
Figure S31. ^{51}V NMR spectra of D_2O solution of **1** and VO_4^{3-} (8 mM) *vs* time at pD = 9.0. The signals denoted with the asterisk are originated from the ^{51}V peaks of $U^{VI}O_2$-(μ-OH)$_2$-$V^{V}O_2^{3+}$-qtn^{4-} species. **S43**
Figure S32. ^{51}V NMR spectra of D_2O solution of **1** and VO_4^{3-} (4 mM) *vs* time at pD = 9.0. The signals denoted with the asterisk are originated from the ^{51}V peaks of $U^{VI}O_2$-(μ-OH)$_2$-$V^{V}O_2^{3+}$-qtn^{4-} species. **S44**
Figure S33. Diagram of concentration of complex **2** (red circles) and complex **5** (black circles *vs* time after the addition in an aqueous solution of complex **1** (2.00 mM) with VO_4^{3-} (20.0) at pD 9.0. **S45**
Figure S34: UV-spectra of **2** (1.00 mM, pH=9.0) without $[UO_2]^{2+}$ (t=0’) and its reaction with 2.00 mM of $[UO_2]^{2+}$ *vs* time (measurement every 7 seconds for 175 seconds) **S46**
Figure S35. ^{1}H NMR spectra of *trans*-$[U^{VI}O_2]^{2+}$ in solution (D_2O, 2.00 mM), H_4enl (4.00 mM) and $V^{V}O_4^{3-}$ (2 – 7 mM) at pD = 10.0. **S47**
Figure S36. ^{1}H NMR spectra (aromatic region) of *trans*-$[U^{VI}O_2]^{2+}$ in solution (D_2O, 2.00 mM), H_4pdl (4.00 mM) and $V^{V}O_4^{3-}$ (2 – 7 mM) at pD = 10.0. **S48**
Figure S37. ^{1}H NMR spectra (aliphatic region) of *trans*-$[U^{VI}O_2]^{2+}$ in solution (D_2O, 2.00 mM), H_4pdl (4.00 mM) and $V^{V}O_4^{3-}$ (2 – 7 mM) at pD = 10.0 **S49**
Figure S38. ^{1}H NMR spectra of *trans*-$[U^{VI}O_2]^{2+}$ in solution (D_2O, 2.00 mM), H_4qtn (4.00 mM) and $V^{V}O_4^{3-}$ (2 – 7 mM) at pD = 10.0. **S50**
Figure S39. Correlation of the ^{1}H NMR chemical shifts of the protons of the dipic^{2-} in complexes **8-10** *vs* FMO Mulliken populations. **S51**

Figure S40. ESI-MS of ligand **H_2bihyat** in MeOH in the presence of 4 drops of aqueous solution of base (NaOH 1M). **S52**
Figure S41. ESI-MS of ligand **H_2dipic** in MeOH in the presence of 4 drops of aqueous solution of base (NaOH 1M). **S52**
Figure S42. ESI-MS of ligand **H_4enl** in MeOH in the presence of 4 drops of aqueous solution of base (NaOH 1M). **S53**
Figure S43. ESI-MS of ligand **H_4pdl** in MeOH in the presence of 4 drops of aqueous solution of base (NaOH 1M). **S53**
Figure S44. ESI-MS of ligand **H_4qtn** in MeOH in the presence of 4 drops of aqueous solution of base (NaOH 1M). **S54**
Figure S45. ESI MS of solutions (H_2O:CH_3OH; 1:1 *v/v*) at pH=9.0 of A) H_4qtn (2 mM) : *trans*-$U^{VI}O_2^{2+}$ (4 mM), B) H_4qtn (2 mM) : *cis*-$V^{V}O_2^{+}$ (4 mM), C) H_4enl (2 mM) : *trans*-$U^{VI}O_2^{2+}$ (4 mM).**S55**
Figure S46. ESI MS of solutions (H_2O:CH_3OH; 1:1 *v/v*) at pH=9.0 of A) H_4qtn (2 mM) : H_2bihyat (4 mM) : *trans*-$U^{VI}O_2^{2+}$ (4 mM), B) H_4pdl (2 mM) : H_2bihyat (4 mM) : *trans*-$U^{VI}O_2^{2+}$ (4 mM), C) H_4qtn (2 mM) : H_2dipic (4 mM) : *trans*-$U^{VI}O_2^{2+}$ (4 mM), D) H_4enl (2 mM) : H_2dipic (4 mM) : *trans*-$U^{VI}O_2^{2+}$ (4 mM). **S56**
Figure S47. ESI-MS of solutions (H_2O:CH_3OH; 1:1 *v/v*) at pH=9.0 of A) H_4qtn (2 mM) : *cis*-$V^{V}O_2^{+}$ (4 mM): *trans*-$U^{VI}O_2^{2+}$ (4 mM), B) H_4enl (2 mM) : *cis*-$V^{V}O_2^{+}$ (4 mM): *trans*-$U^{VI}O_2^{2+}$ (4 mM). **S57**
Scheme 1. The chemdraw structures in the scheme shows the chemical inequivalence of the two methyl groups of the hydroxylamine of the half part of the BL and depict: A) Exchange mechanisms between the hydroxylamine methyl groups (a) resonance form A (Scheme 5) rotation around C(5)-X bond, B) resonance form B (Scheme 5) flip of C(5)-X bond centred on X. Bond resonance of BLs in metal complexes based on the crystal **S58**
Table S1. Crystal Data and Structure Refinement for the compounds **1** and **2**. **S61**
Table S2. Crystal Data and Structure Refinement for the compounds **3, 4** and **6**. **S63**
Table S3. Interatomic Distances (Å) and Angles (deg) Relevant to the U^{VI} and V^{V} Coordination Sphere. **S64**
Table S4. Interatomic Distances (Å) and Angles (deg) Relevant to the U^{VI} and V^{V} Coordination Sphere. **S66**
Table S5. DFT Optimized Structures. **S69**

Experimental

Materials and Methods. All chemicals and solvents were purchased from Merck. Microanalyses for C, H, and N were performed using a Euro-Vector EA3000 CHN elemental analyzer. FT-IR transmission spectra of the compounds, were acquired using a Shimadzu IRSpirit model spectrophotometer. The kinetic UV-vis measurements were recorded on a Photonics UV-vis spectrophotometer Model 400, equipped with a CCD array, operating in the range 250 to 1000 nm. The ligand H_2bihyat was synthesized according to the literature. Merck silica gel 60 F_{254} TLC plates were used for thin layer chromatography.

Single X-ray crystal Structure Analysis. Single crystal analysis was performed on an Xcalibur Oxford Diffractometer equipped with a Sapphire 3 CCD detector and a 4-cycle Kappa geometry goniometer, using enhanced Mo Kα (λ=0.71073 Å) X-ray source and graphite radiation monochromator. Analytical absorption correction was applied using CrysAlis RED software. CrysAlis CCD and CrysAlis RED software were used for data collection and data reduction/cell refinement respectively. The structure of the compounds was solved by direct methods and refined by full-matrix least-squares techniques on F^2 using SHELXS-97.[1] Special computing molecular graphics incorporated in the WinGX 3.2 interface were used. All the non-H atoms were anisotropically refined. The positions of hydrogen atoms in all structures were calculated from stereochemical considerations and kept fixed isotropic during refinement or found in DF map and refined with isotropic thermal parameters.

NMR and UV-vis Spectroscopies.

NMR measurements. All NMR samples were prepared from crystalline uranium(VI) compounds in D_2O at room temperature just prior to NMR spectrometric determinations. NMR spectra were recorded on a Bruker Avance III 500 MHz spectrometer. A 30°-pulse width was applied for both the ^1H and ^{13}C NMR measurements, and 2 s relaxation delay. The samples were prepared from purified molecules at room temperature just prior to NMR spectrometric measurements. Data acquisition and processing were accomplished using TopSpin 4.0.6 and MultiSpecNMR 4.0.0 (https://sourceforge.net/projects/multispecnmr/). Standard pulse programs as implemented in TopSpin were used for data acquisition.

Sample Preparation for ^{1}H & ^{51}V NMR and UV-Vis measurements. The samples in D_2O at standard 1 : 2 ratios of ligand to metals at different pH values were prepared as follows: The ligand [H_4qtn, 0.0125 g / H_4pdl 0.0125 g / H_4enl, 0.0110 g] was dissolved in 4.90 mL of D_2O achieving a final concentration of 5.70 mM. Due to the fact that the ligands have low solubility in water, 2.0 – 5.0 μL of NaOD 6M were added in order to dissolve them completely in D_2O. $[U^{VI}O_2(NO_3)_2(H_2O)_2]\cdot 4H_2O$ (0.0309 g) and $NaV^{V}O_3$ (0.0076 g) were dissolved in 5.00 mL of D_2O each, achieving a final concentration of 5.70 mM. From these solutions, samples that contain 2 mM of the ligand and 4 mM of $U^{VI}O_2^{2+}$/ VO_2^+ were prepared by mixing 350.0 μL of the ligand's solution with 325.0 μL of the appropriate metal solution. After that, D_2O was added to achieve a final volume of 1.00 mL leading to the complexes' **1-5** concentration of 2.00 mM. The pD was adjusted with 2.0 – 10.0 μL additions using 0.5 M NaOD/DCl solutions prior to the measurements.

For the UV-Vis measurements, the solutions were prepared as mentioned above using H_2O and adding the appropriate portion of water to achieve the desirable concentration.

Mixed Metal NMR Experiments. Sample Preparation. Stock solutions of the ligands [H_4qtn, 0.0125 g / H_4pdl 0.0125 g / H_4enl, 0.0110 g], $[U^{VI}O_2(NO_3)_2(H_2O)_2]\cdot 4H_2O$ (0.0281 g) and were prepared by dissolving each substance in 4.900 mL of D_2O and their concentration was 5.7 mM for the ligand, 11.4 mM for $[U^{VI}O_2(NO_3)_2(H_2O)_2]\cdot 4H_2O$. Due to the fact that, the ligands have low solubility in water, 2.0 – 5.0 μL of NaOD 6M were added in order to dissolve them completely in D_2O. Stock solution for $NaV^{V}O_3$ (0.1 M) was made by dissolving 0.0610 g of the substance in 5.00 mL of D_2O. For mixed metal experiments, each sample was prepared by mixing 350.0 μL of the ligand solution with the appropriate quantity (20.0, 30.0, 40.0, 50.0, 60.0 and 70.0 μL) of the $NaV^{V}O_3$ solution. The pD was then adjusted to be lower than 10 to assure the ligand-vanadate complexation. Finally, 350.0 μL of the $[U^{VI}O_2(NO_3)_2(H_2O)_2]\cdot 4H_2O$ solution was added followed by fixing the final volume of each sample at 1.00 mL using D_2O. The final concentrations of the samples were 2 mM of the ligand, 4 mM of $[U^{VI}O_2(NO_3)_2(H_2O)_2]\ \cdot 4H_2O$, and varying (2, 3, 4, 5, 6, 7 mM) of $NaV^{V}O_3$. ^{1}HNMR measurements were recorded at pDs ~7, and 9. The pH was adjusted with 2.0 – 10.0 μL additions using 0.5 M NaOD/DCl solutions prior to the measurements. For the kinetic experiments, the 350.0 μL of the uranyl solution was firstly mixed with 350 μL of the ligand's solution, the pH was adjusted to the desired pD, and the appropriate quantity of the $NaV^{V}O_3$ solution was added prior to the measurement.

^{1}H NMR spectroscopy was used for kinetic experiments. The reaction rates of the kinetic experiments were calculated from the initial reaction rates *vs* reactants concentration. The error was calculated from three experiments.

Mixed Ligand NMR Experiments. Sample Preparation. Stock solutions of the ligands [H_4qtn, 0.0135 g / H_4pdl, 0.0134 g / H_4enl, 0.0120 g] and $[U^{VI}O_2(NO_3)_2(H_2O)_2]\cdot 4H_2O$ (0.0300 g) were prepared by dissolving each substance in 9.000 and 1.500 mL of D_2O respectively so that their concentrations were adjusted at 3.33 mM for the ligands and 40 mM for $[U^{VI}O_2(NO_3)_2(H_2O)_2]\cdot 4H_2O$. H_2bihyat, dipicolinic acid (H_2dipic) and CO_3^{2-} stock solutions were prepared by dissolving H_2bihyat (0.1026 g), H_2dipic (0.0670 g) or $NaHCO_3$ (0.3360 g) in 2.00 mL of D_2O in order to achieve a concentration of 0.20 M, 0.20 M and 2.00 M respectively. Due to the fact that all ligands have low solubility in water, 2.0 – 5.0 μL of NaOD 6M were added in order to completely dissolve the ligands in D_2O. For the mixed ligand experiments, each sample was prepared by mixing 600.0 μL of the ligands solution with the appropriate quantity of the competing ligand's solution (5.0, 10.0, 20.0, 40.0 μL), or the carbonate solution (6, 15, 45, 60, 100, 140 and 200) and then 100.0 μL of the $[U^{VI}O_2(NO_3)_2(H_2O)_2]\cdot 4H_2O$. solution. For more concentrated D_2O solutions, standard solutions of higher concentration were used. In each sample, the appropriate quantity of D_2O was added to achieve a final volume of 1.000 mL and a final concentration of 2 mM of the H_4qtn ligand, 4mM of the $[U^{VI}O_2(NO_3)_2(H_2O)_2]\cdot 4H_2O$ and varying (1, 2, 4, 8, 20 mM) of the competing ligands or (12, 30, 90, 120, 200, 280 and 400 mM) of the carbonate solution. ^{1}HNMR measurements were recorded at pDs ~7, and 9. The pD was adjusted with 2.0 – 10.0 μL additions using 0.5 M NaOD/DCl solutions prior to the measurements.

In all NMR experiments except the kinetic studies the samples were heated before measurements, in order to reach thermodynamic equilibrium between the species in the solution. Each experiment was repeated at least five times.

Vanadium complexes' formation constant determination experiments. NMR Studies. The experimental procedure used for this experiment was the same mentioned above. Standard solutions of the ligands (5.70 mM) were prepared in D_2O, from which 350 μL were used for each sample. The variable Vanadates' concentrations were achieved by using the appropriate quantities of the 5.70 mM $NaVO_3$ mother solution. In each sample, the appropriate quantity of D_2O was added to achieve a final volume of 1.000 mL. The pH was adjusted with 2.0 – 10.0 μL additions using 0.5 M NaOD/DCl solutions prior to the measurements.

Vanadium complexes' formation constant determination experiments. UV Studies. For the UV measurements, all solutions were prepared in aqueous 0.1 M tris buffer solution (pH=9.0). The ligands' concentrations were kept constant throughout the titrations at 0.1, 0.5 and 0.1 mM for H_4qtn, H_4pdl and H_4enl respectively by using 200 μL from each ligand's mother solution (0.01 M). Vanadates concentrations varied from 0.05 to 2.20 mM by using the appropriate volume from a $NaVO_3$ mother solution (0.08 M) prepared in tris buffer (0.1 M, pH=9.0). All solutions used for the UV experiments were adjusted to a final volume of 2.00 mL by adding the appropriate quantity of the previously prepared tris buffer solution (0.1 M, pH=9.0).

Determination of commercial $NaVO_3$ purity. The purity of the commercial $NaVO_3$ was determined as peroxovanadate by UV-Vis spectroscopy. First, the molecular absorptivity (ε) of peroxovanadate was found using 99.95% V_2O_5. The determination of ε was carried out by reacting of V_2O_5 with excess of H_2O_2 (3 equivalents) in 0.5 M H_2SO_4.[2] Various concentrations of this solution were plotted against the absorption at 453 nm. The molecular absorptivity, ε=188.39 mol L^{-1} cm^{-1} per vanadium, was calculated from eight samples.

$NaVO_3$ reacted with excess of H_2O_2 (3 equivalents) in 0.5 M H_2SO_4 leading to the characteristic, red-colored solution. The concertation of vanadium in this solution was calculated from the absorbance at 453 nm. The parity of the $NaVO_3$ salt was calculated as the mean value of eight samples containing various amounts of vanadium and found to be 89.42 %.

Electrospray Ionization Mass Spectrometry (ESI-MS)

All MS data were collected using a Q-trap, time-of-flight MS (Maxis Impact MS) instrument supplied by Bruker Daltonics Ltd. The detector was a time-of-flight, micro-channel plate detector and all data was processed using the Bruker Daltonics Data Analysis 4.1 software, whilst simulated isotope patterns were investigated using Bruker Isotope Pattern software and Molecular Weight Calculator 6.45. The calibration solution used was Agilent ES tuning mix solution, Recorder No. G2421A, enabling calibration between approximately 100 *m/z* and 2000 *m/z*. This solution was diluted 60:1 with MeCN. Samples were dissolved in H_2O:MeOH solvent mixture and introduced into the MS *via* direct injection at 180 μL h^{-1}. The ion polarity for all MS scans recorded was negative, at 180 ^{o}C, with the voltage of the capillary tip set at 4000 V, endplate offset at –500 V, funnel 1 RF at 300 Vpp and funnel 2 RF at 400 Vpp.

Computational Methods

The Amsterdam Modelling Suite[3] version 2020.101 was used as the numerical tool. The revised[4] PBE[5] functional with Grimme's 4th generation dispersion correction[6] (rev-PBE-D4) was employed throughout as a computational protocol. The chosen Slater type atomic basis sets were of TZP quality for the metal atoms (U,V) and DZP quality for the main group atoms. A [He] frozen core was chosen

for the light elements C,N,O, [Ne] for vanadium and [Lu^{3+}] for uranium. The COSMO[7] implicit solvation model was used in the free energy estimations with default parameters for water (ε=78.4). The stationary points were all characterized as minima upon calculation of the vibrational frequencies.

For the hydration reactions of the anionic oxo species the experimental free energy of the auto-ionization reaction of water (pK_w=13.997 at 25ºC) was used, +19.1 kcal mol^{-1}, to calculate the global reaction schemes mentioned in the text.

The Fragment Molecular Orbital analysis concerned the two $[U^{VI}O_2(H_2O)_2]^{2+}$ fragments plus the BL^{4-} ligands. Each fragment was calculated as closed shell (spin-restricted) and with the same implicit solvation as the whole molecule.

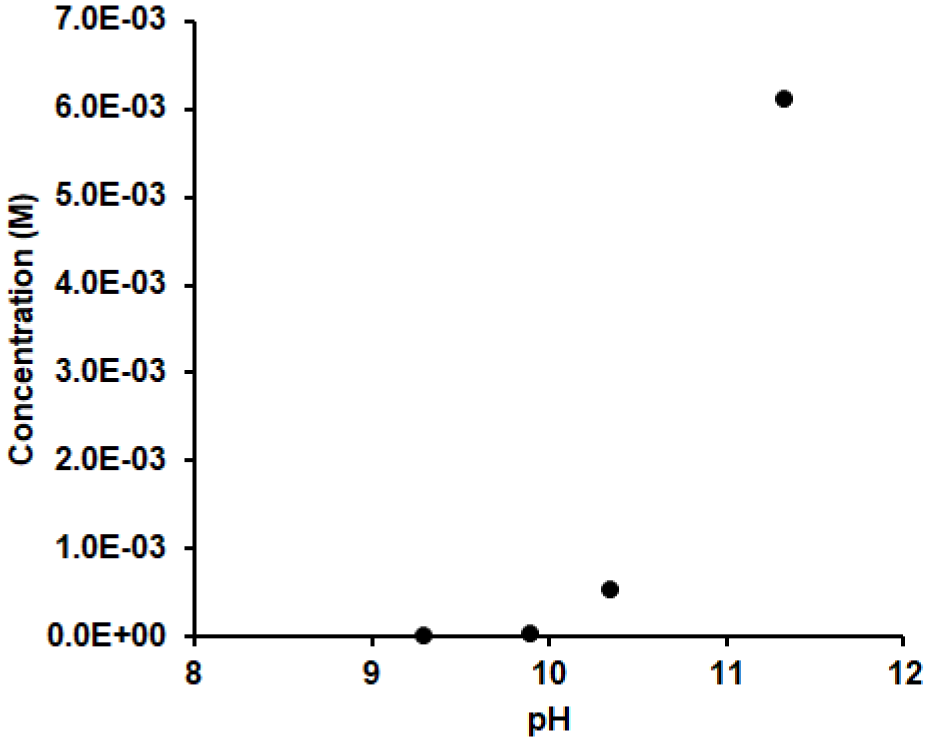

Figure S1. Solubility of H_4qtn *vs* pH. Based on ^{1}H NMR and using CH_3COONa as internal standard for quantification.

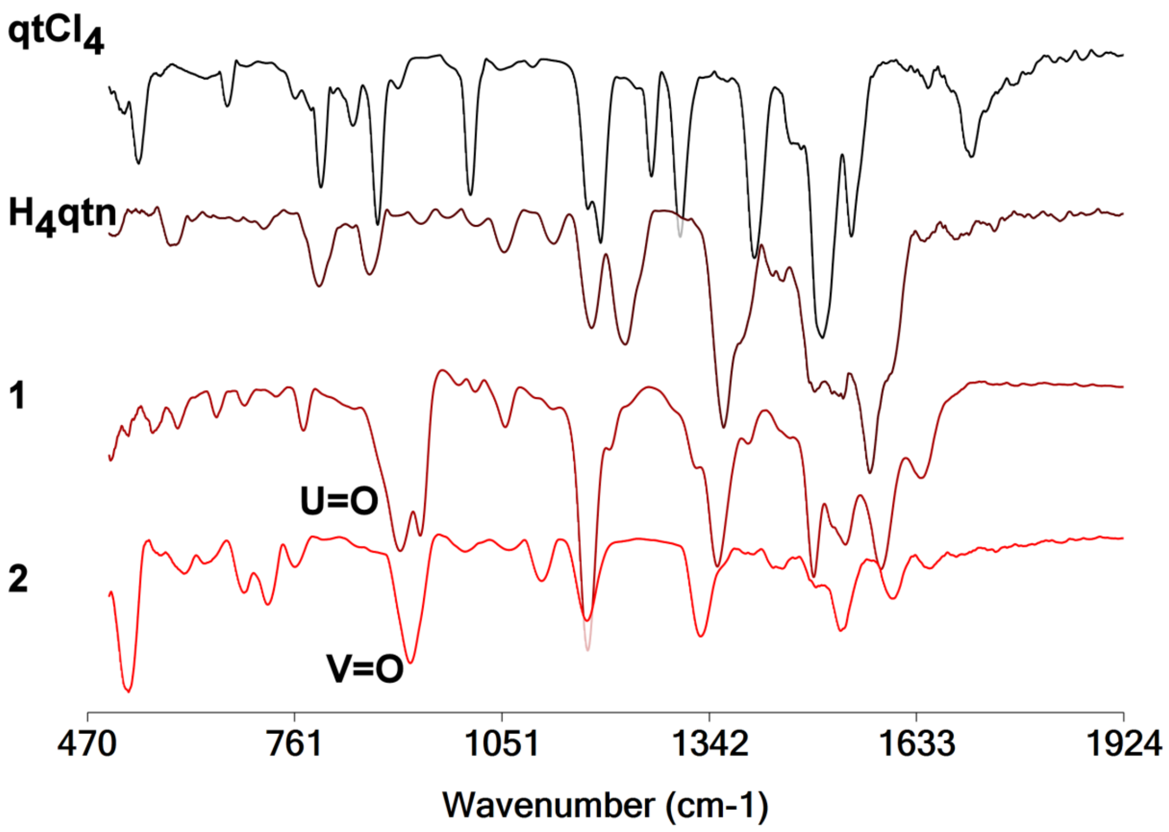

Figure S2. IR spectrum of $qtCl_4$, H_4qtn, **1** and **2**.

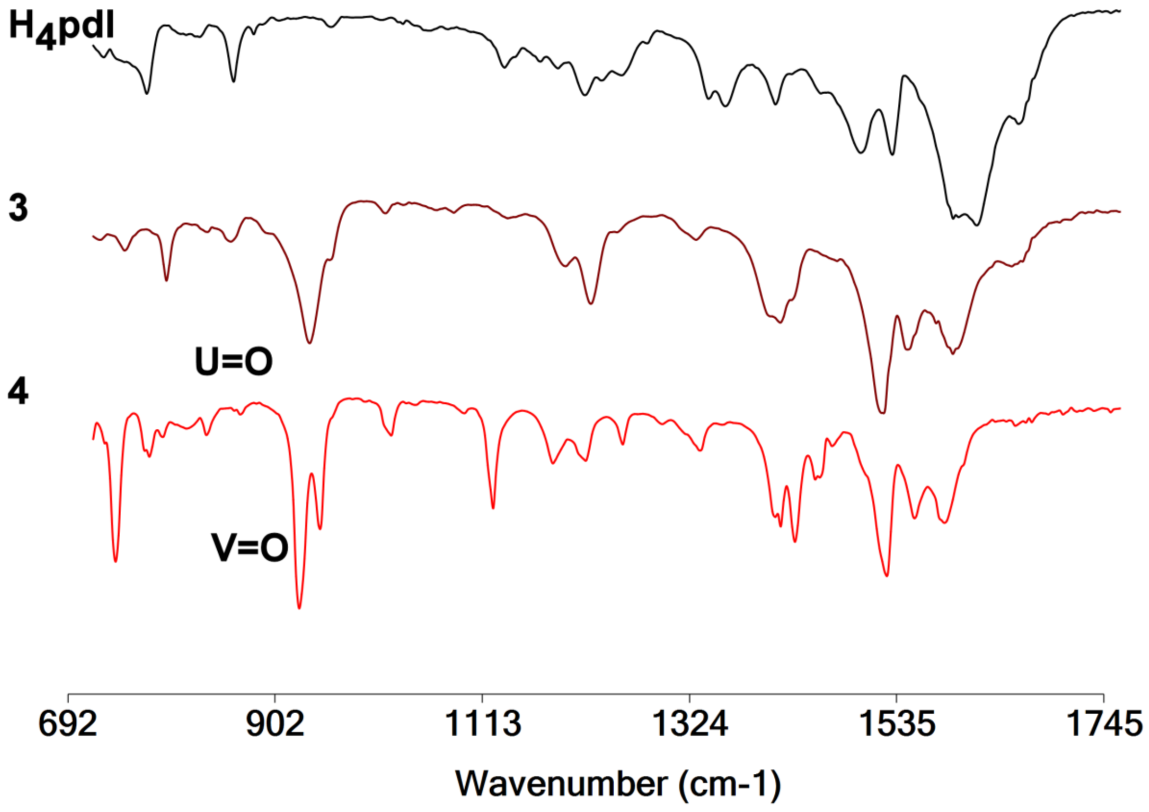

Figure S3. IR spectrum of H_4pdl, **3** and **4**.

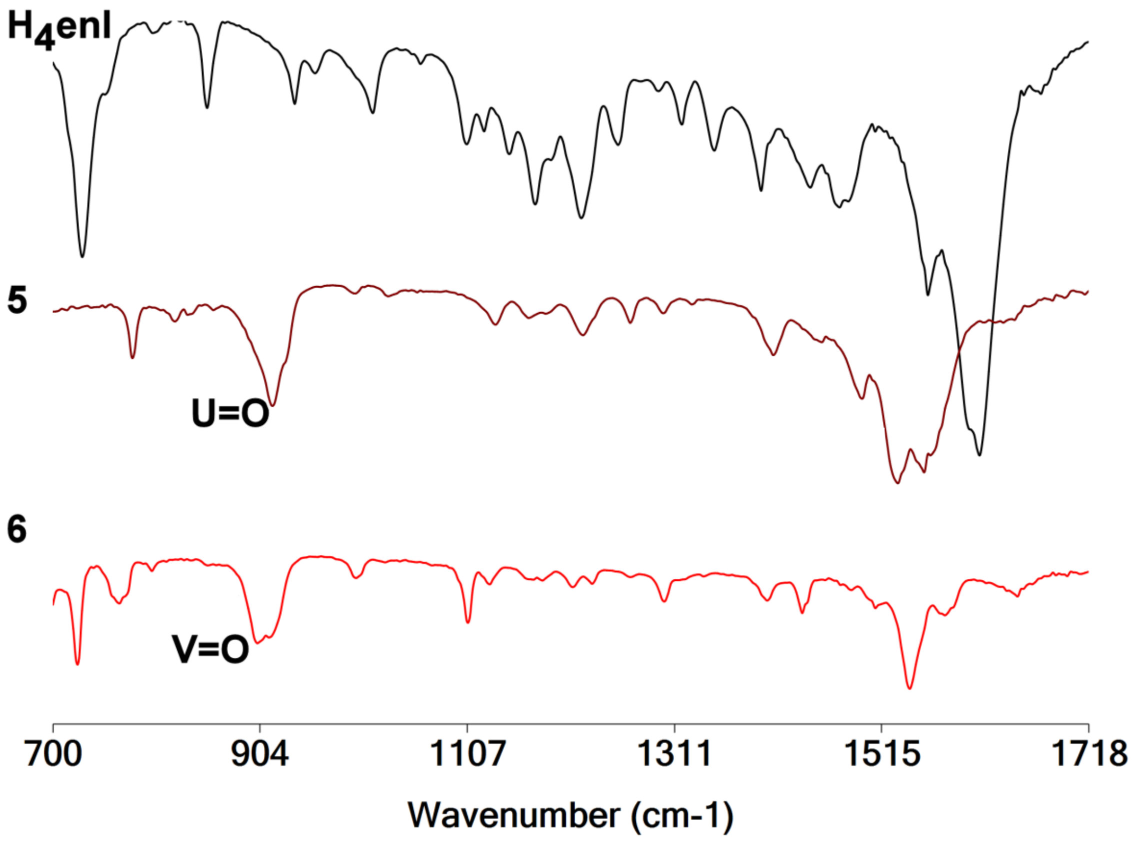

Figure S4. IR spectrum of H_4enl, **5** and **6**.

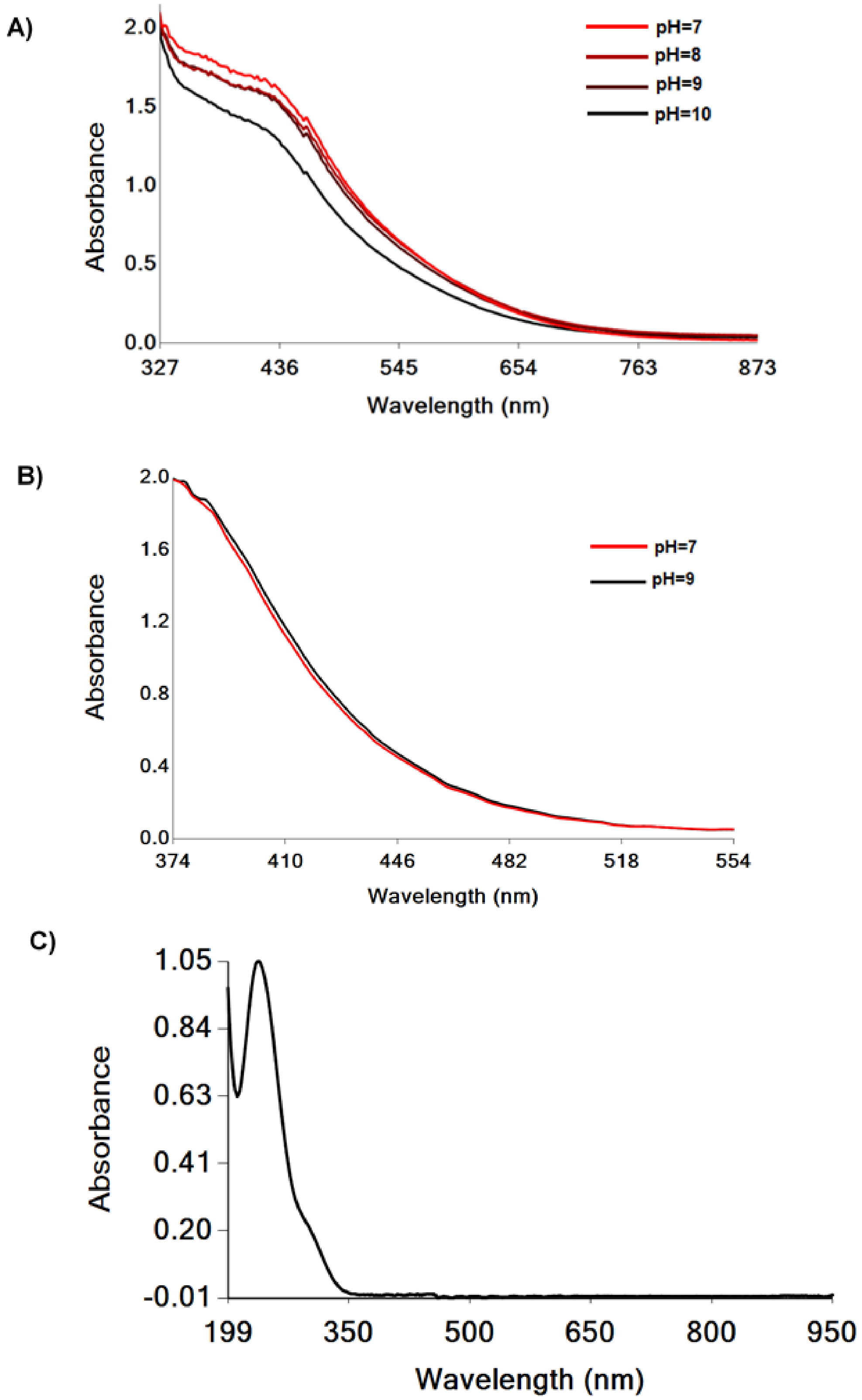
A)
pH=7
pH=8
pH=9
pH=10
Absorbance
2.0
1.5
1.0
0.5
0.0
327
436
545
654
763
873
Wavelength (nm)
B)
pH=7
pH=9
Absorbance
2.0
1.6
1.2
0.8
0.4
0.0
374
410
446
482
518
554
Wavelength (nm)
C)
Absorbance
1.05
0.84
0.63
0.41
0.20
-0.01
199
350
500
650
800
950
Wavelength (nm)

Figure S5. Variable pH UV-spectra of A) **1** (1 mM), B) **2** (0.75 mM) and C) qtn^{4-} (0.05 mM) at pH 11.8.

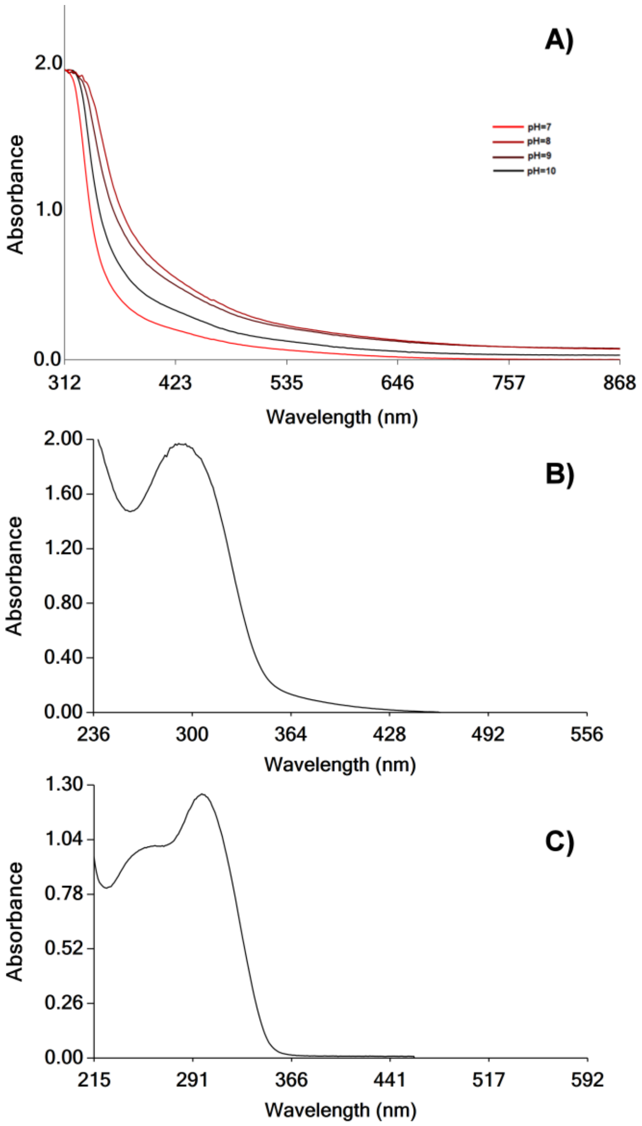

Figure S6. Variable pH UV-spectra of A) **3** (0.125 mM, pHs 7-10), B) **4** (0.125 mM, pHs 7-10) and C) pdl^{4-} (0.100 mM) at pH 12.

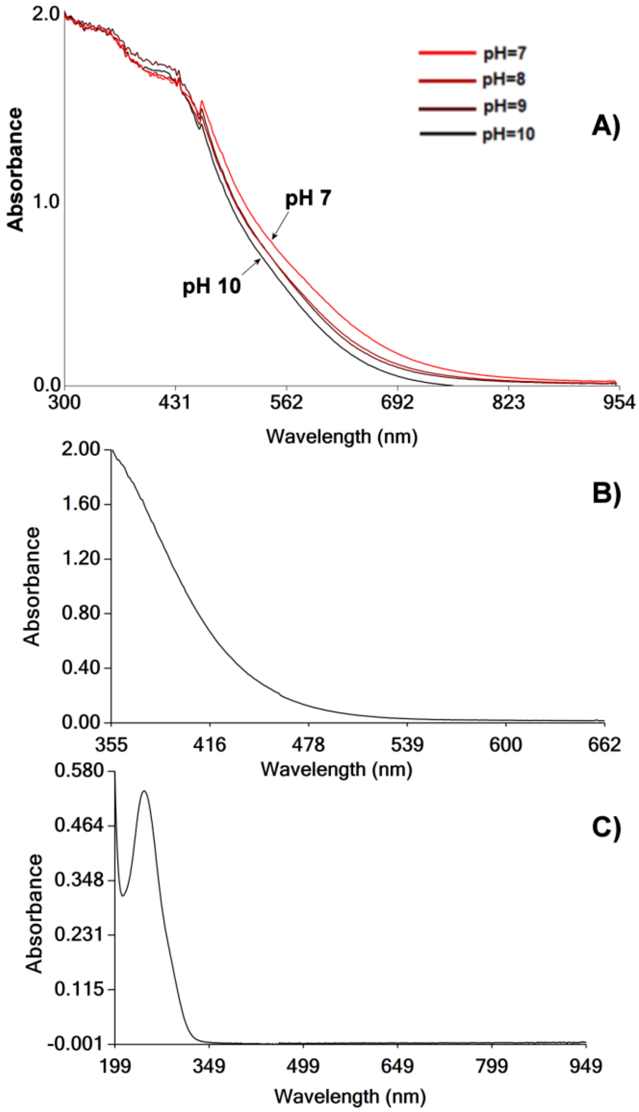

Figure S7. Variable pH UV-spectra of A) **5** (2.00 mM, pHs 7-10), B) **6** (0.500 mM, pHs 7-10) and C) enl^{4-} (0.125 mM) at pH 12.

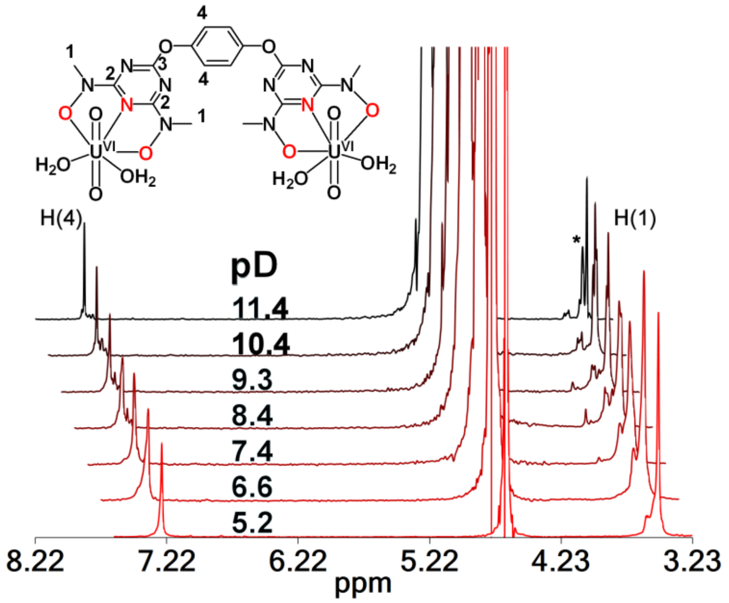

Figure S8. ^{1}H NMR spectra of **1** in solution (D_2O) (2.00 mM) at various pDs. The signals denoted with the asterisk are due to the H(1) peaks of $U^{VI}O_2$-(μ-OH)$_2$-$U^{VI}O_2^{4+}$-enl^{4-} species.

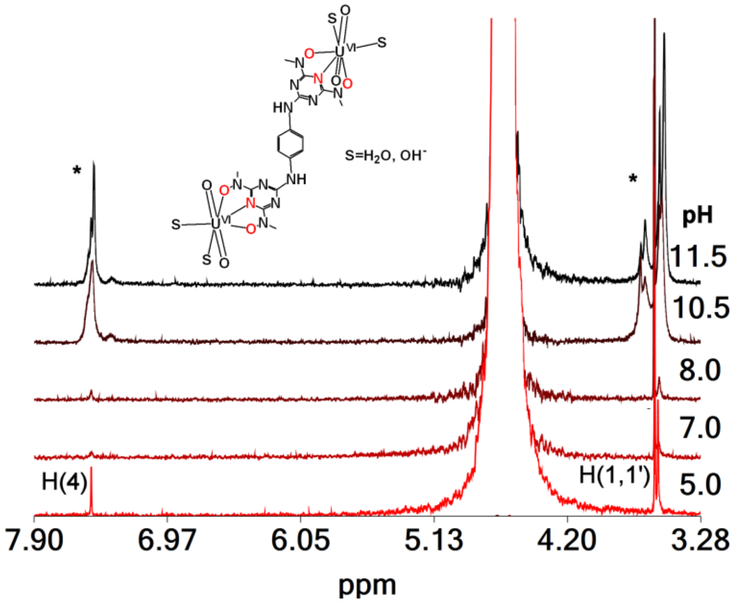

Figure S9. ^{1}H NMR spectra of **3** in solution (D_2O) (2.00 mM) at various pDs. The signals denoted with the asterisk are due to the H(1) peaks of $U^{VI}O_2$-(μ-OH)$_2$-$U^{VI}O_2{}^{4+}$-enl^{4-} species.

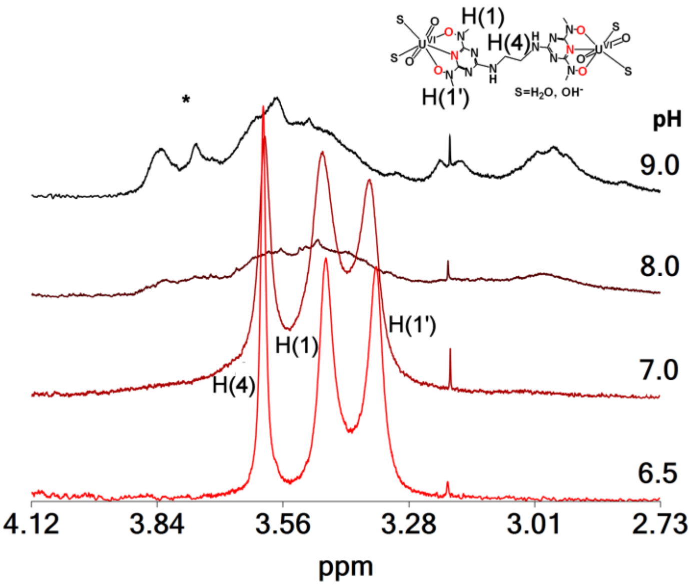

Figure S10. ^{1}H NMR spectra of **5** in solution (D_2O) (2.00 mM) at various pDs. The signals denoted with the asterisk are due to the H(1) peaks of $U^{VI}O_2$-(μ-OH)$_2$-$U^{VI}O_2^{4+}$-enl^{4-} species.

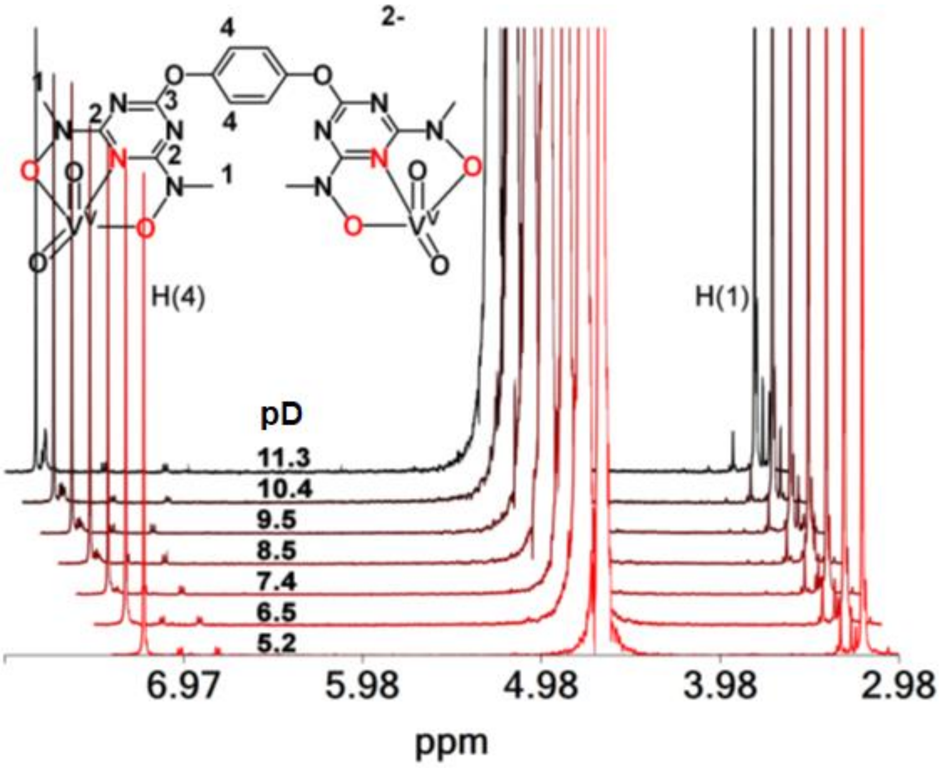

Figure S11. ^{1}H NMR spectra of D_2O solutions of **2** (2.00 mM) at various pDs

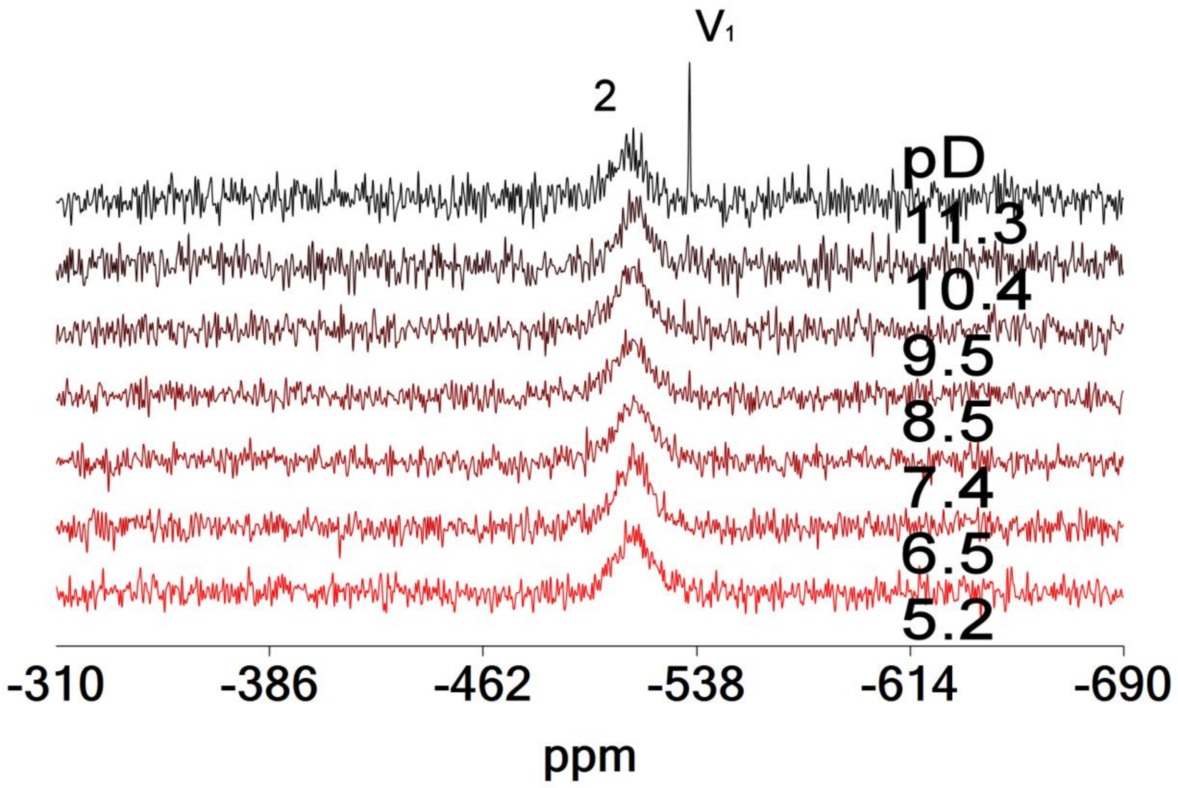

Figure S12. ^{51}V NMR spectra of D_2O solutions of **2** (2.00 mM) at various pDs. V_1 is VO_4^{3-}.

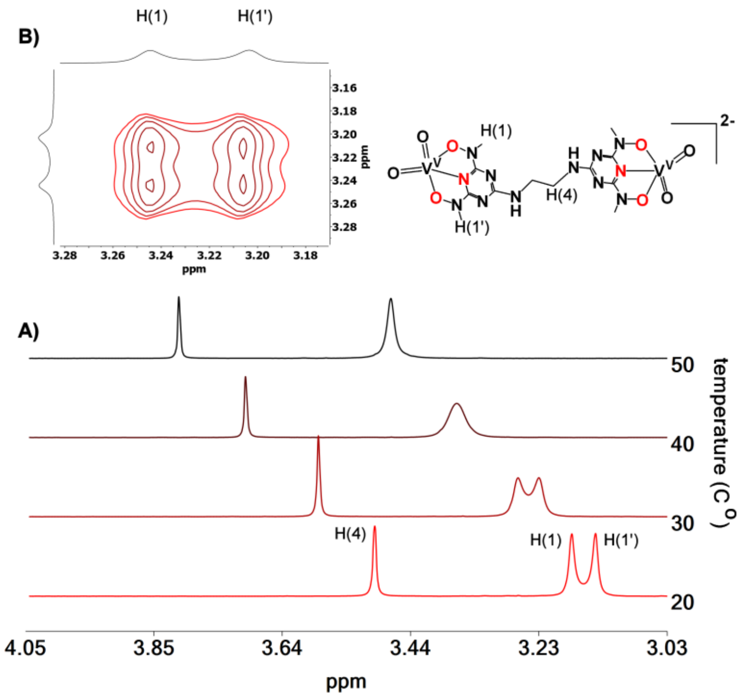

Figure S13. A) VT ^{1}H NMR spectra of **6** in solution (D_2O) (2.00 mM) at pH=10, B) 2D **{**^{1}H} EXSY NMR spectra of **6** in solution (D_2O) (2.00 mM) at pH=10 and 20 ^{o}C.

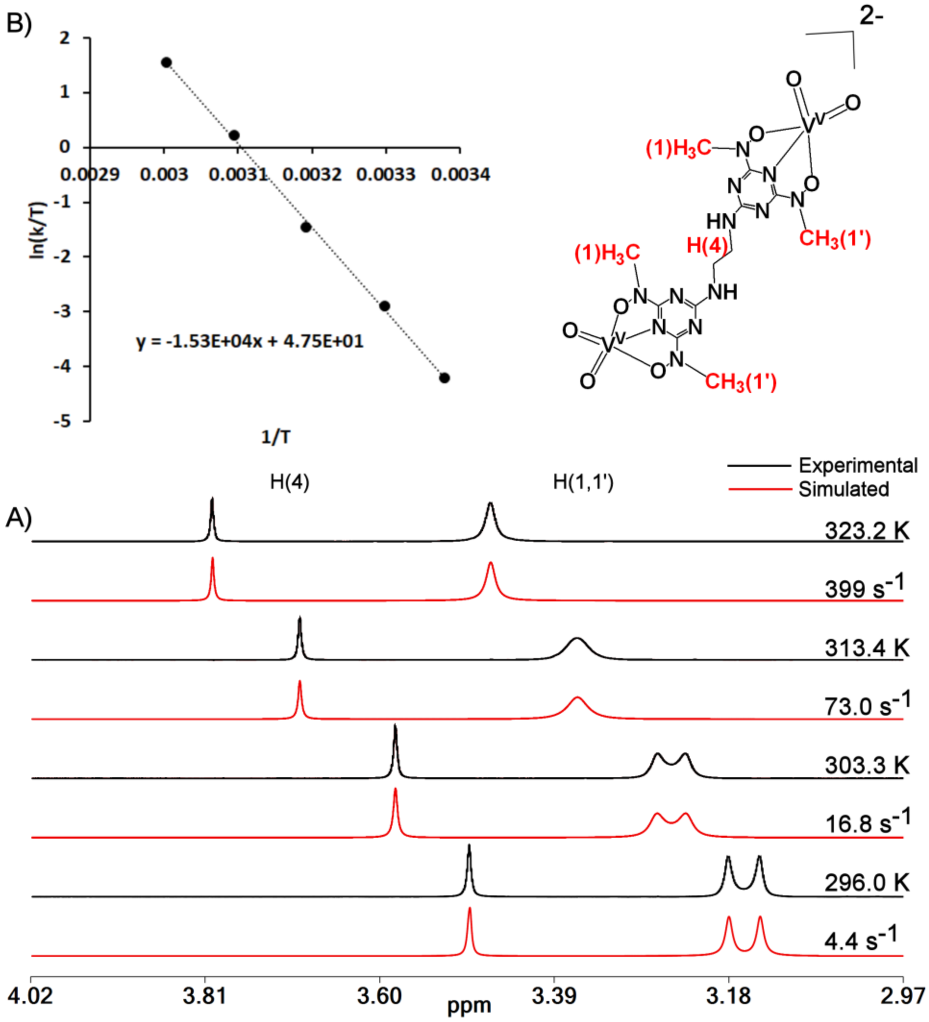

Figure S14. A) VT ^{1}H NMR spectra, experimental and simulated, of **6** in solution (D_2O) (2.00 mM) at pH=10, B) Eyring plot ln(*k*/T) *vs* 1/T, $\Delta S^{\#}$=197 J/mol, $\Delta H^{\#}$=127 kJ/mol/K.

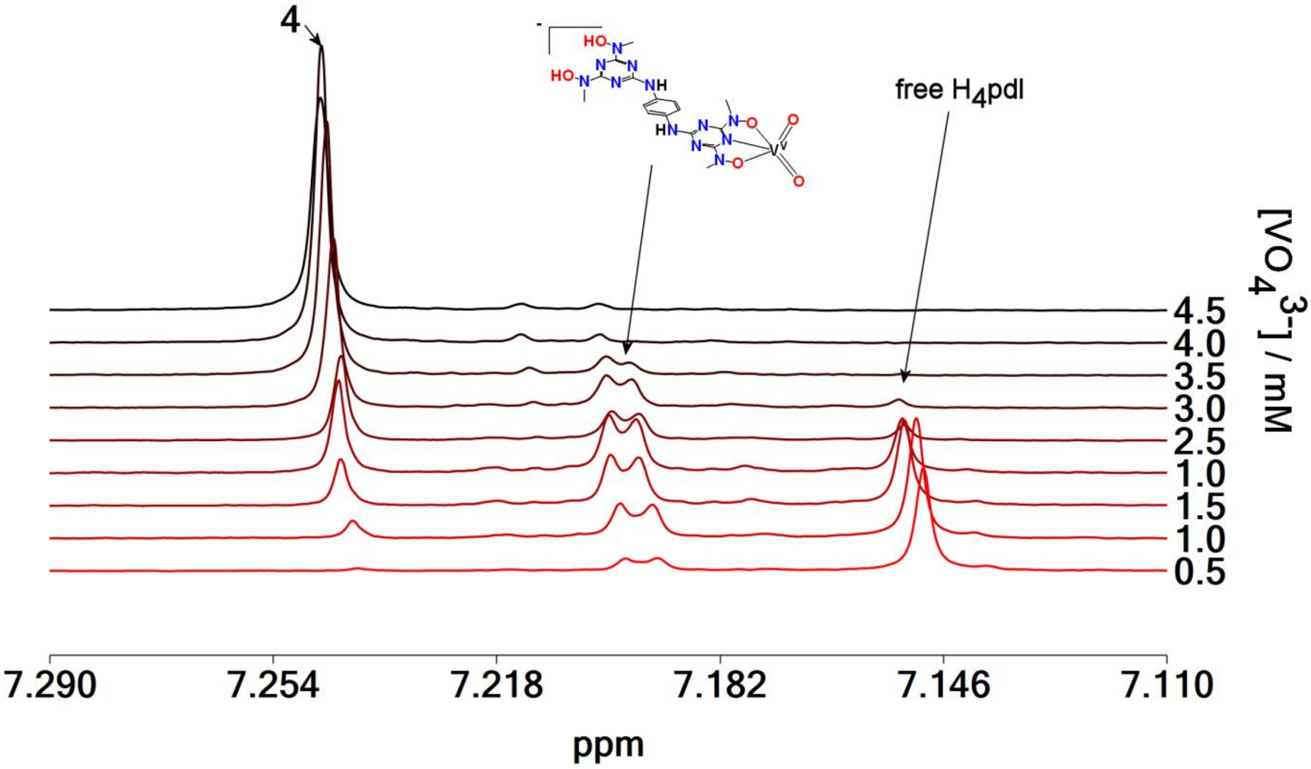

Figure S15. ^{1}H NMR spectra of the titration of Hpdl [in solution (D_2O), 2.00 mM] by VO_4^{3-} (0-4.50 mM) at pD = 9.0.

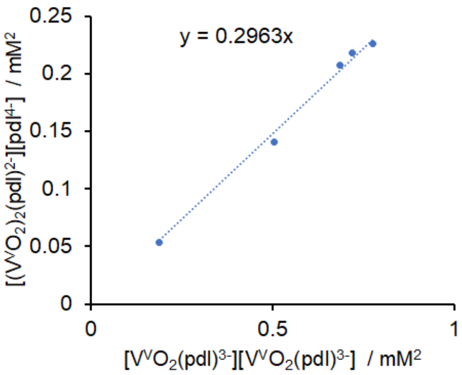

a)

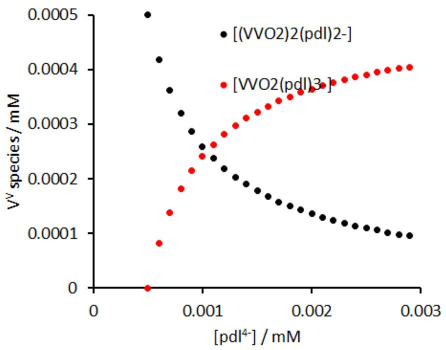

b)

Figure S16. a) Diagram for the calculation of K_{2pdl} from the ^{1}H NMR of Figure S15, b) speciation diagram considering 1.00 mM of the total concentration of $[VO_4^{3-}]$.

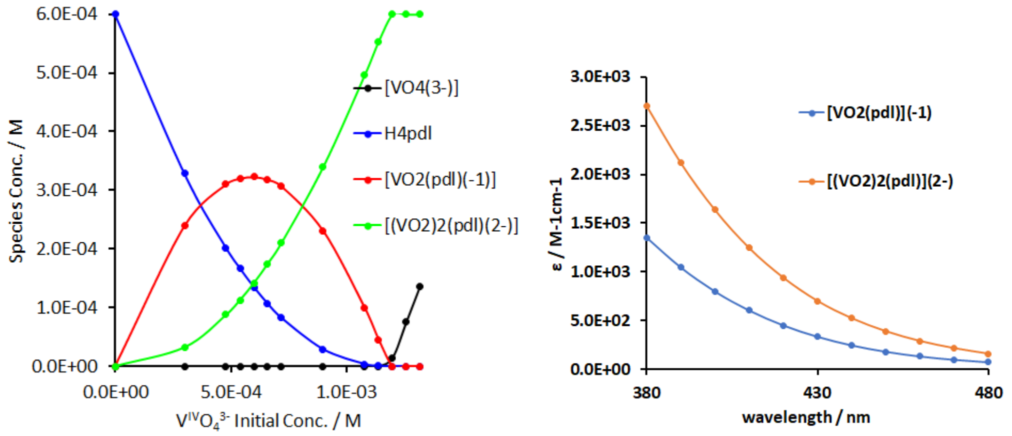

Figure S17. a) Speciation diagram based on the spectroscopic titration of 6.000 x10^{-4} mM H_4pdl and addition of various quantities of [VO_4^{3-}] at pH 9.1. The points are the experimental concentrations used for the titration experiment. b) Predicted spectra for [V^VO_2(pdl)]$^{-1}$ and the experimental for [(V^VO_2)$_2$(pdl)]$^{2-}$

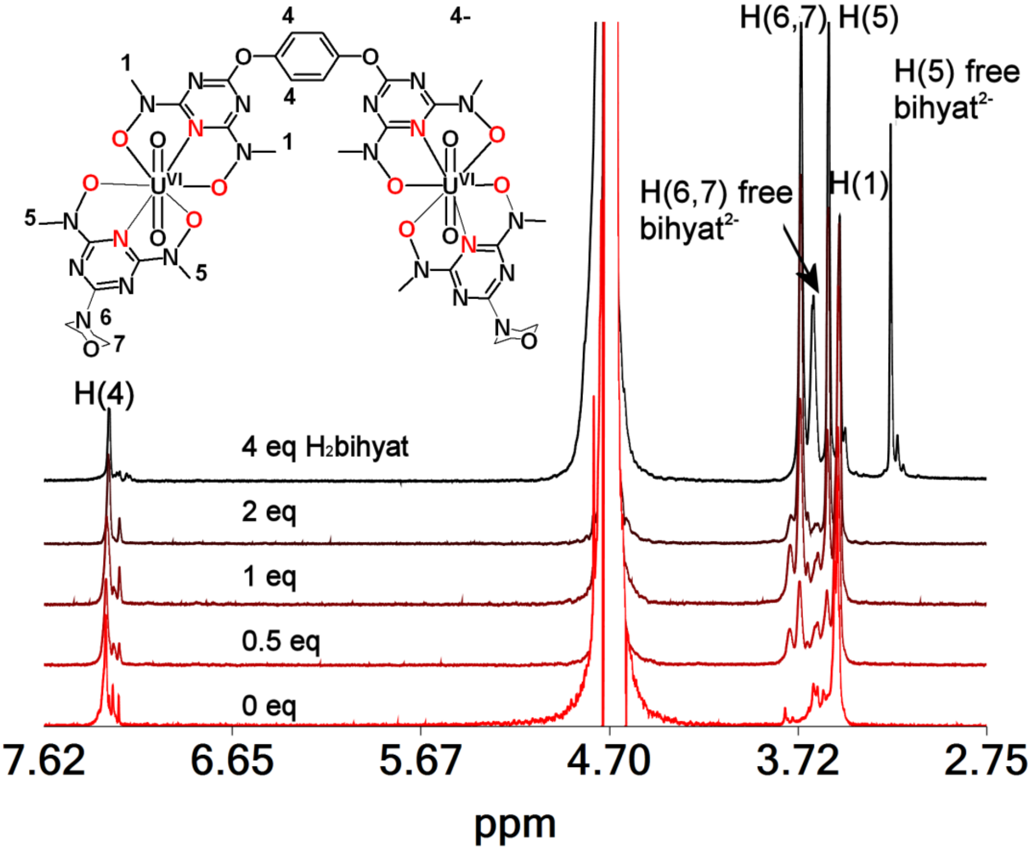

Figure S18. ^{1}H NMR spectra of D_2O solution of $U^{VI}O_2^{2+}$ (2.00 mM), H_2qtn (4.00 mM) and H_2bihyat (0 – 8 mM) at pD = 9.0

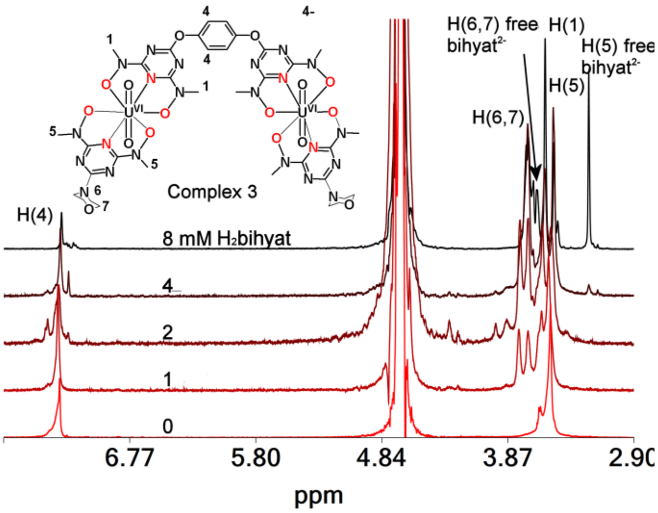

Figure S19. ^{1}H NMR spectra of *trans*-$[U^{VI}O_2]^{2+}$ [in solution (D_2O), 2.00 mM], H_4qtn (4.00 mM) and H_2bihyat (0 – 8 mM) at pD = 7.0

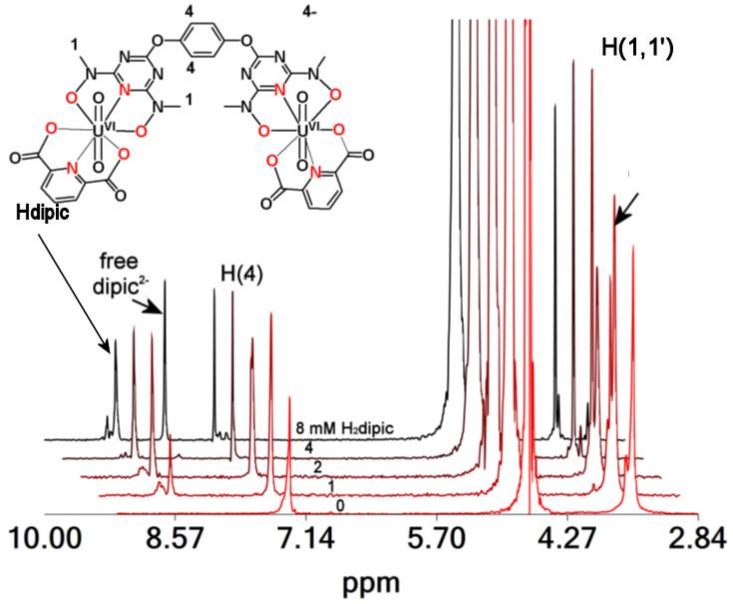

Figure S20. ^{1}H NMR spectra of *trans*-$[U^{VI}O_2]^{2+}$ [in solution (D_2O), 2.00 mM], H_4qtn (4.00 mM) and H_2dipic (0 – 8 mM) at pD = 7.0.

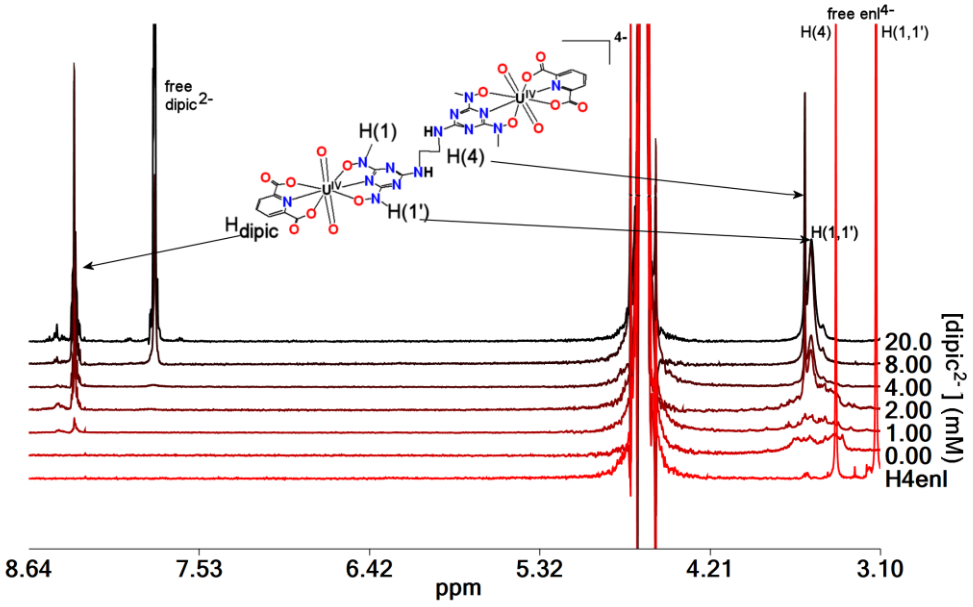

Figure S21. ^{1}H NMR spectra of *trans*-$[U^{VI}O_2]^{2+}$ [in solution (D_2O), 2.00 mM], H_4pdl (4.00 mM) and H_2dipic (0 – 40 mM) at pD = 10.0.

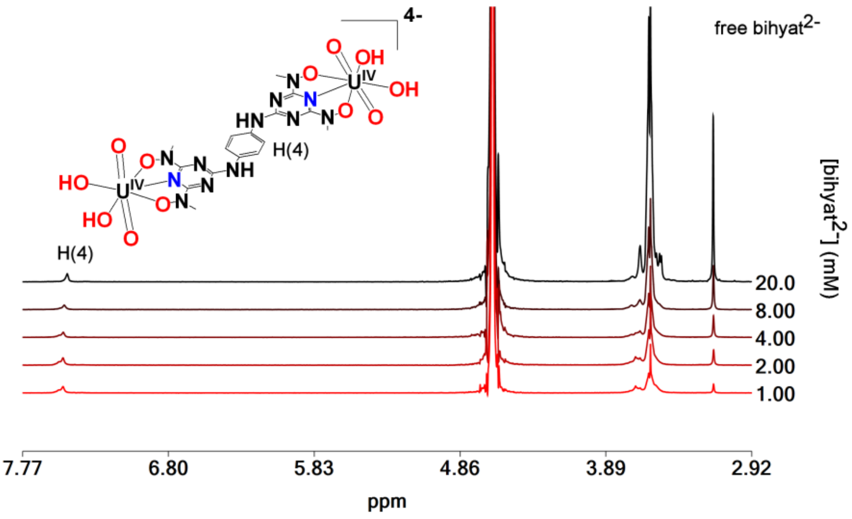

Figure S22. ^{1}H NMR spectra of *trans*-$[U^{VI}O_2]^{2+}$ [in solution (D_2O), 2.00 mM], H_4pdl (4.00 mM) and H_2bihyat (0 – 20 mM) at pD = 9.0

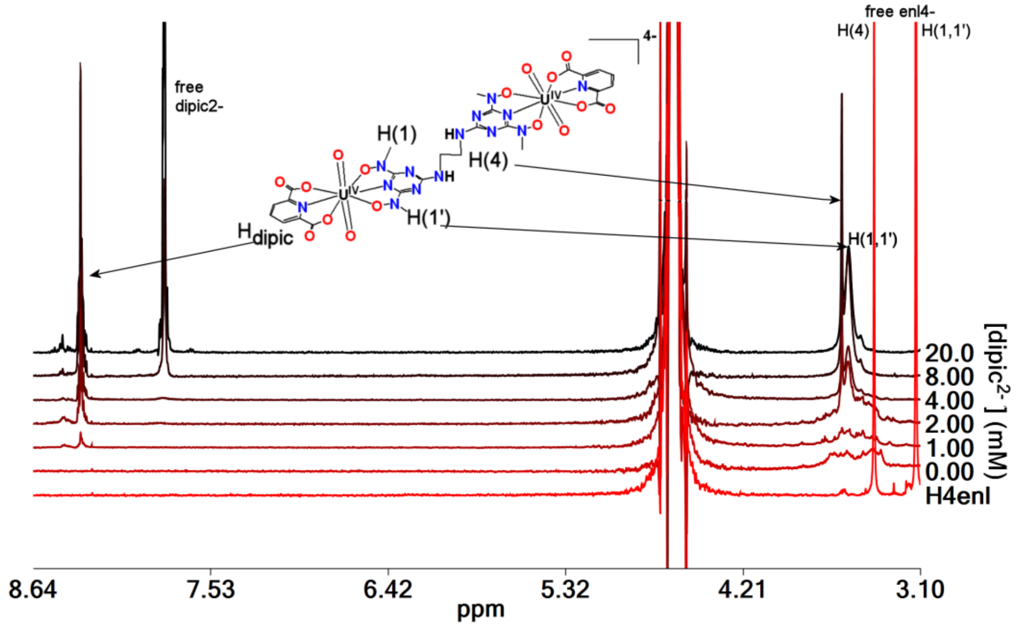

Figure S23. ^{1}H NMR spectra of *trans*-$[U^{VI}O_2]^{2+}$ [in solution (D_2O), 2.00 mM], H_4enl (4.00 mM) and H_2dipic (0 – 20 mM) at pD = 10.0.

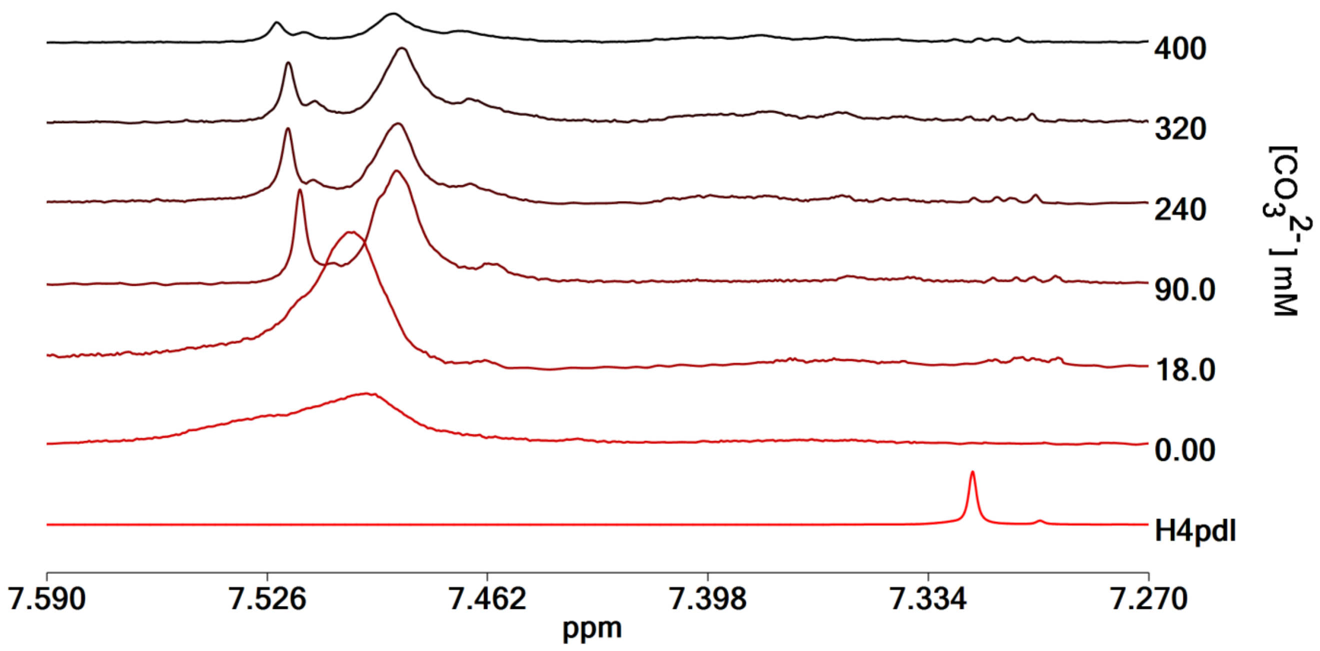

Figure S24. ^{1}H NMR spectra of *trans*-$[U^{VI}O_2]^{2+}$ [in solution (D_2O), 2.00 mM], H_4pdl (4.00 mM) and Na_2CO_3 (0 – 400 mM) at pD = 9.0.

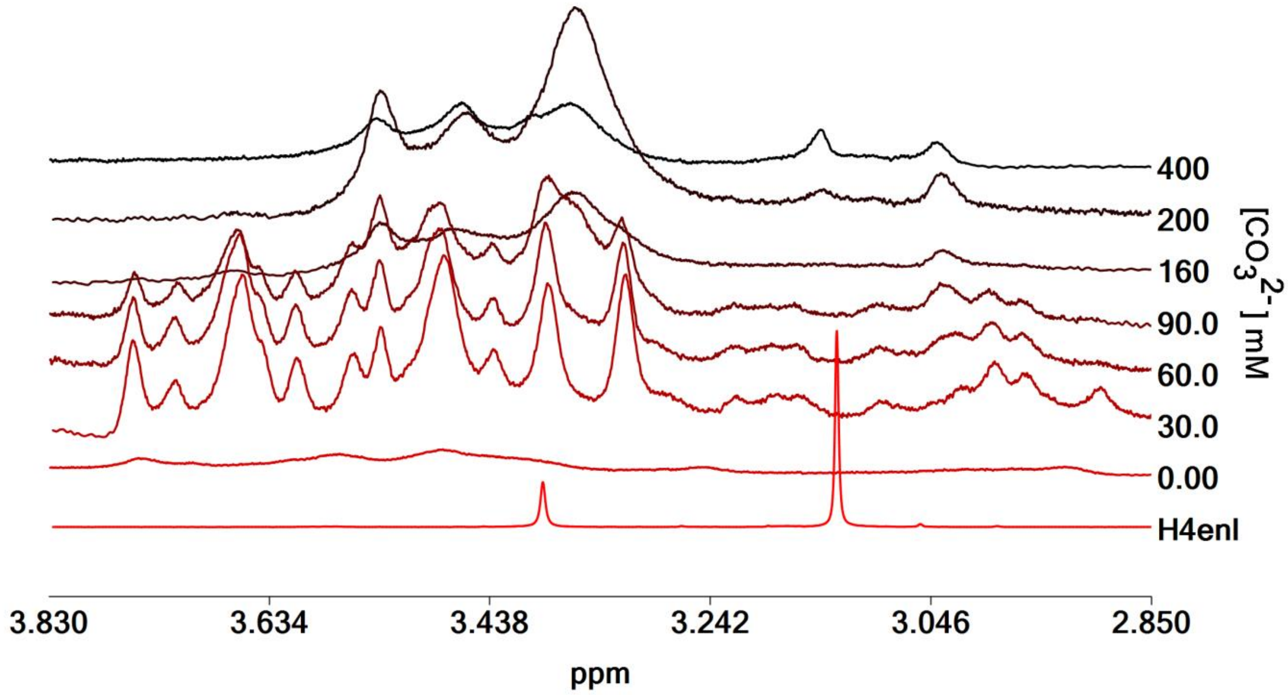

Figure S25. ^{1}H NMR spectra of *trans*-$[U^{VI}O_2]^{2+}$ [in solution (D_2O), 2.00 mM], H_4enl (4.00 mM) and Na_2CO_3 (0 – 400 mM) at pD = 9.0.

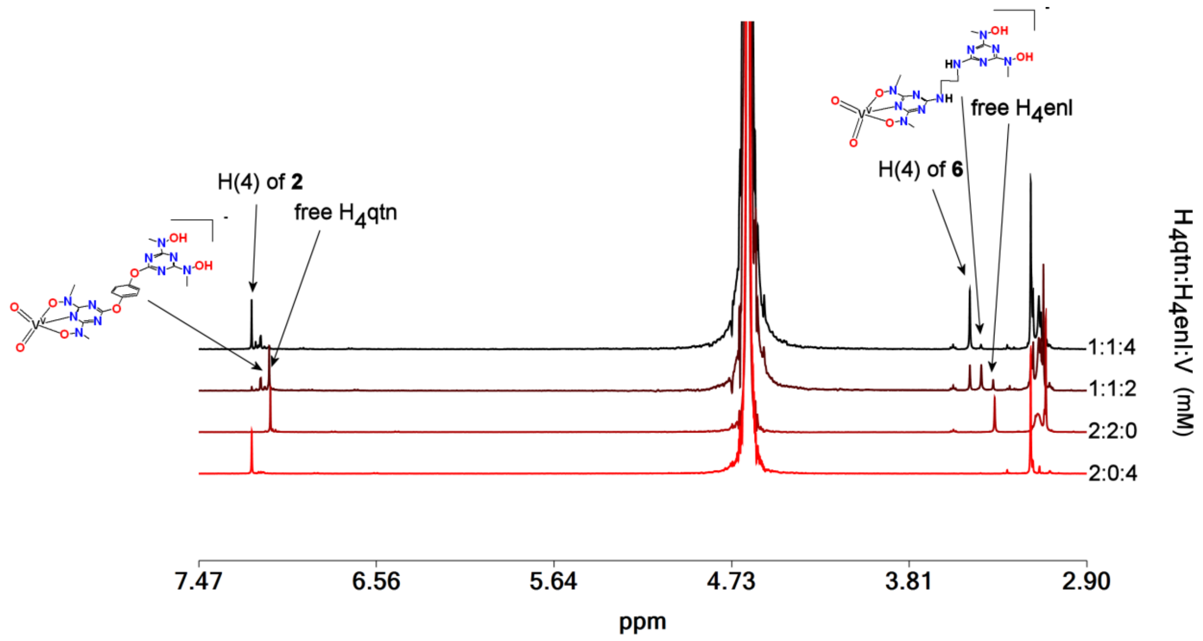

Figure S26. ^{1}H NMR (aromatic region) of D_2O solution containing H_4enl, H_4pdl and VO_3^{4-} at various concentration at pD=9.0.

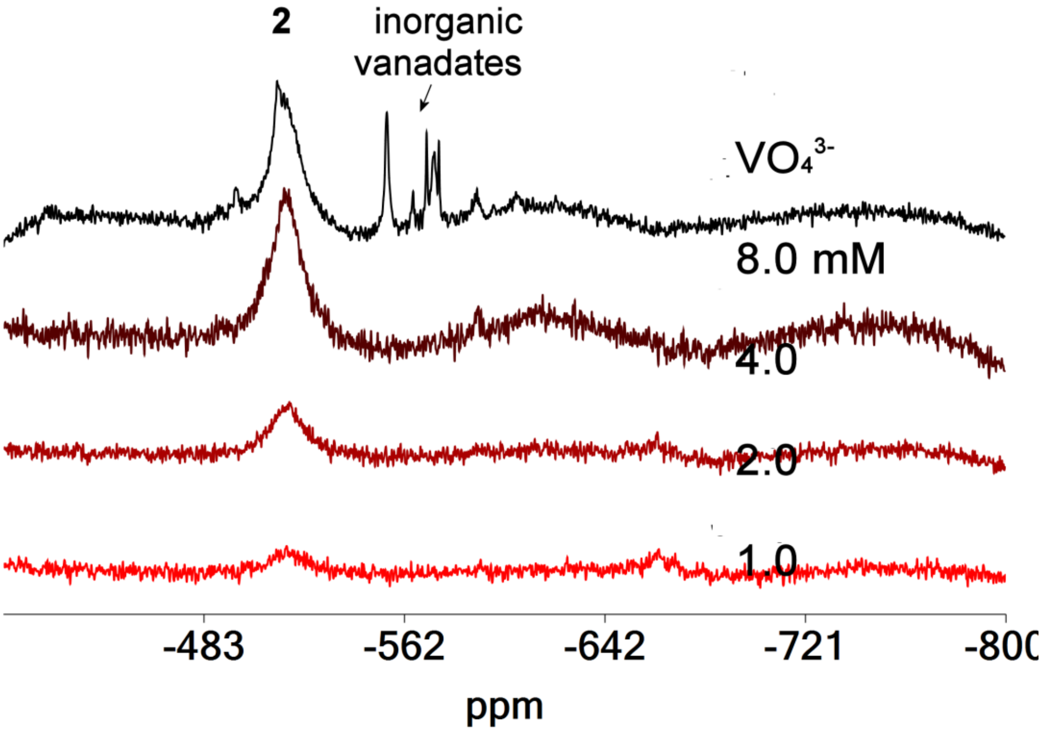

Figure S27. ^{51}V NMR spectra of D_2O solution of $U^{VI}O_2^{2+}$ (2.00 mM), H_2qtn (4.00 mM) and VO_4^{3-} (0 – 20 mM) at pD = 7.0

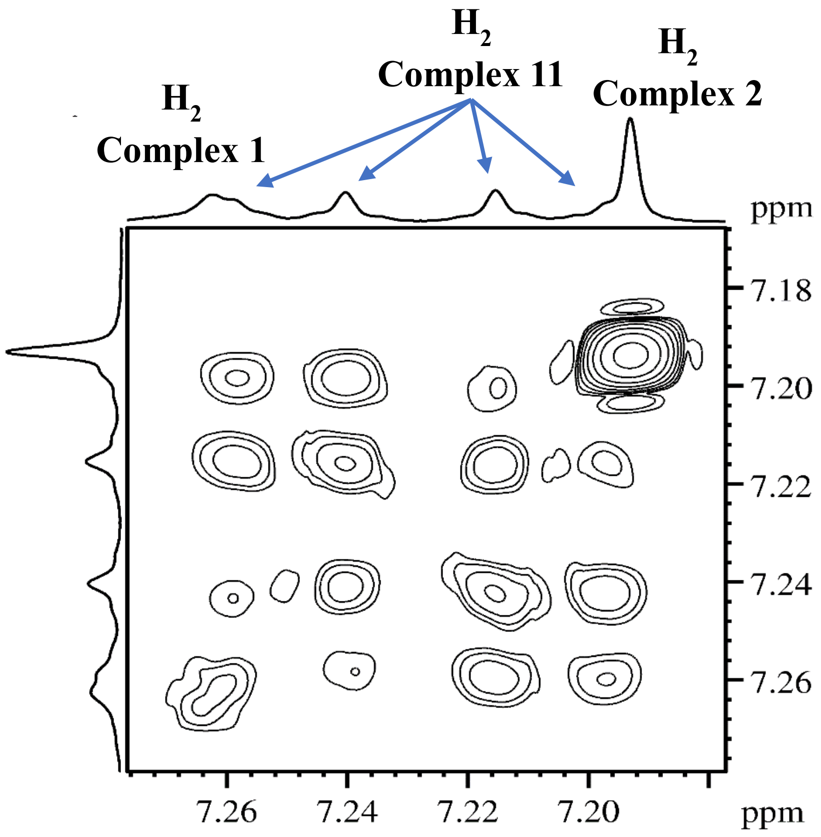

Figure S28. 2D {^{1}H}COSY of the aromatic part of the ^{1}H NMR spectra of D_2O solution of **1** and VO_4^{3-} (20 mM) at pD = 9.0

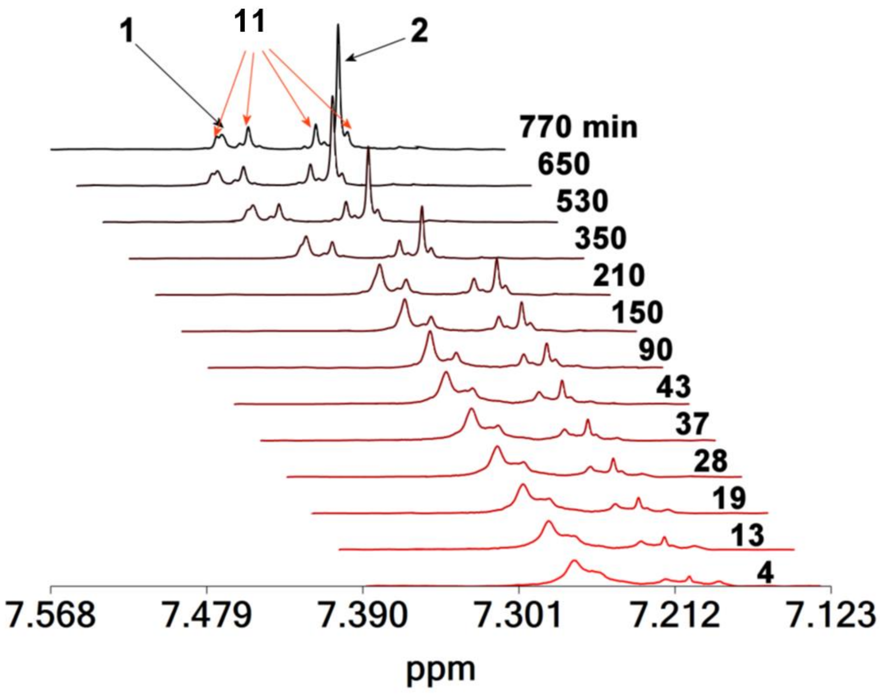

Figure S29. Aromatic part of the ^{1}H NMR spectra of D_2O solution of **1** and VO_4^{3-} (20 mM) at pD = 9.0 *vs* time.

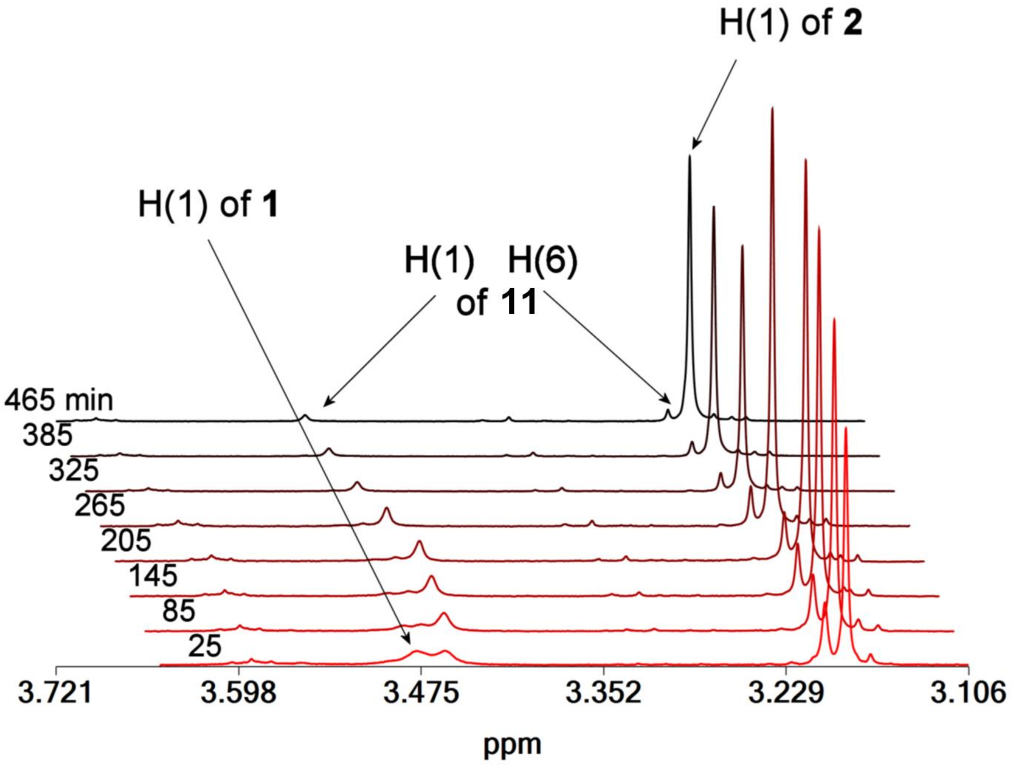

Figure S30. Aliphatic part of the ^{1}H NMR spectra of D_2O solution of **1** and VO_4^{3-} (20 mM) at pD = 7.0 *vs* time. The signals denoted with the asterisk are originated from the H(1) peaks of $U^{VI}O_2$-(μ-OH)$_2$-$U^{VI}O_2^{4+}$-qtn^{4-} species. Numbering according to Figure 5.

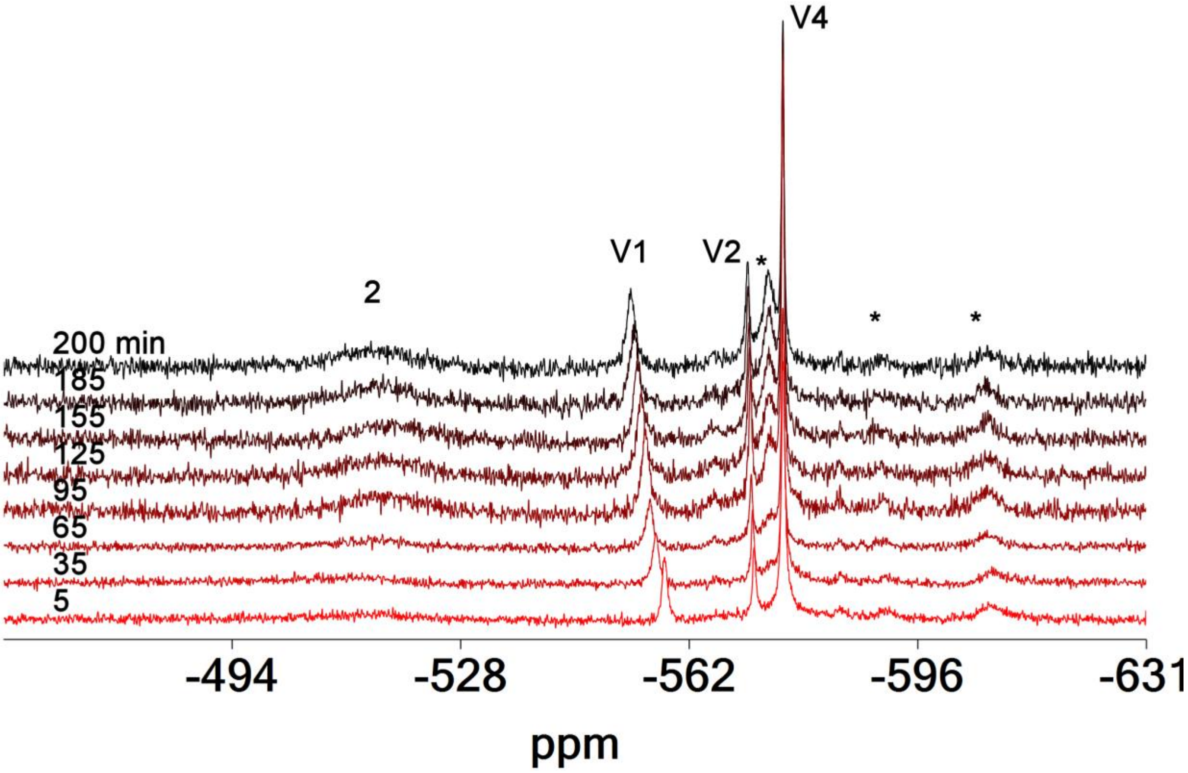

Figure S31. ^{51}V NMR spectra of D_2O solution of **1** and VO_4^{3-} (8 mM) *vs* time at pD = 9.0. The signals denoted with the asterisk are originated from the ^{51}V peaks of $U^{VI}O_2$-(μ-OH)$_2$-$V^VO_2^{3+}$-qtn^{4-} species .

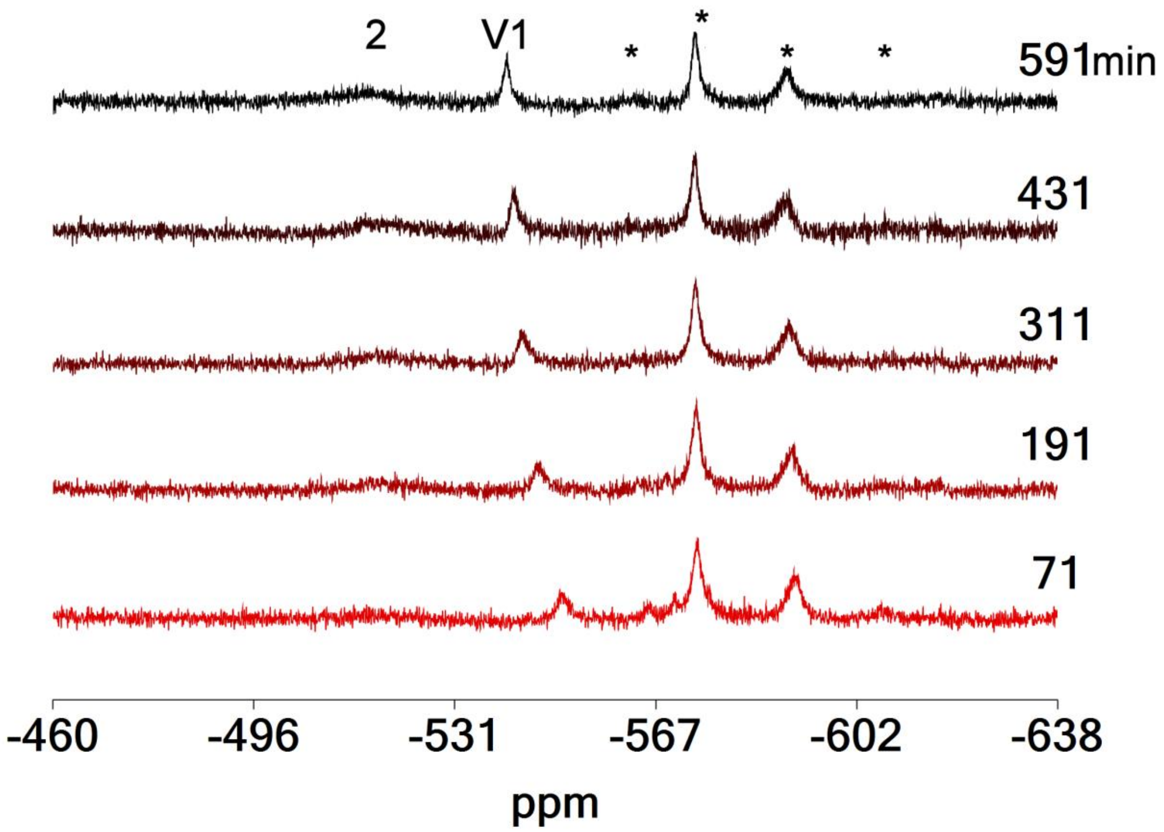

Figure S32. ^{51}V NMR spectra of D_2O solution of **1** and VO_4^{3-} (4 mM) *vs* time at pD = 9.0. The signals denoted with the asterisk are originated from the ^{51}V peaks of $U^{VI}O_2$-(μ-OH)$_2$-$V^{V}O_2^{3+}$-qtn^{4-} species.

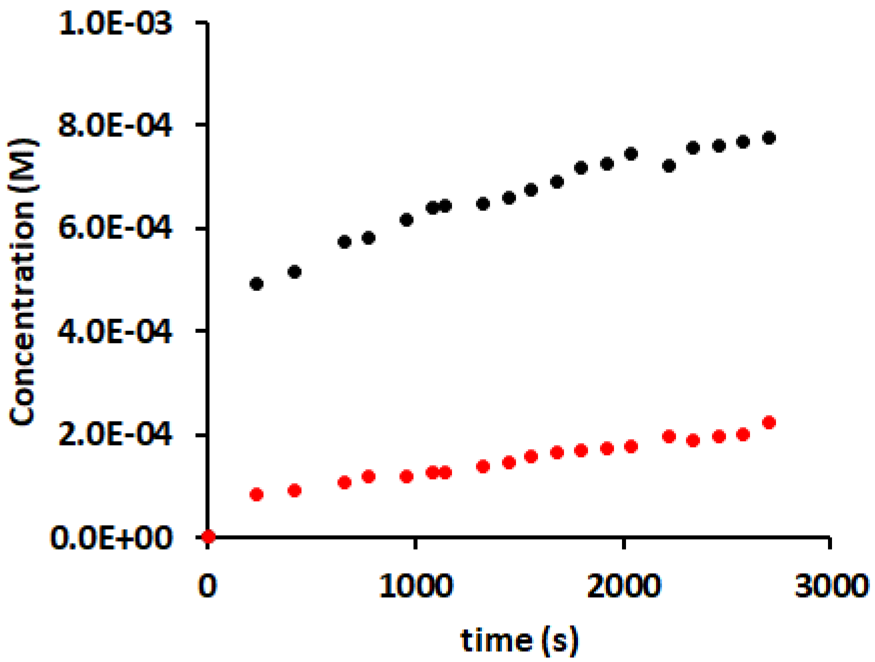

Figure S33. Diagram of concentration of complex **2** (red circles) and complex **5** (black circles *vs* time after the addition in an aqueous solution of complex **1** (2.00 mM) with VO_4^{3-} (20.0) at pD 9.0.

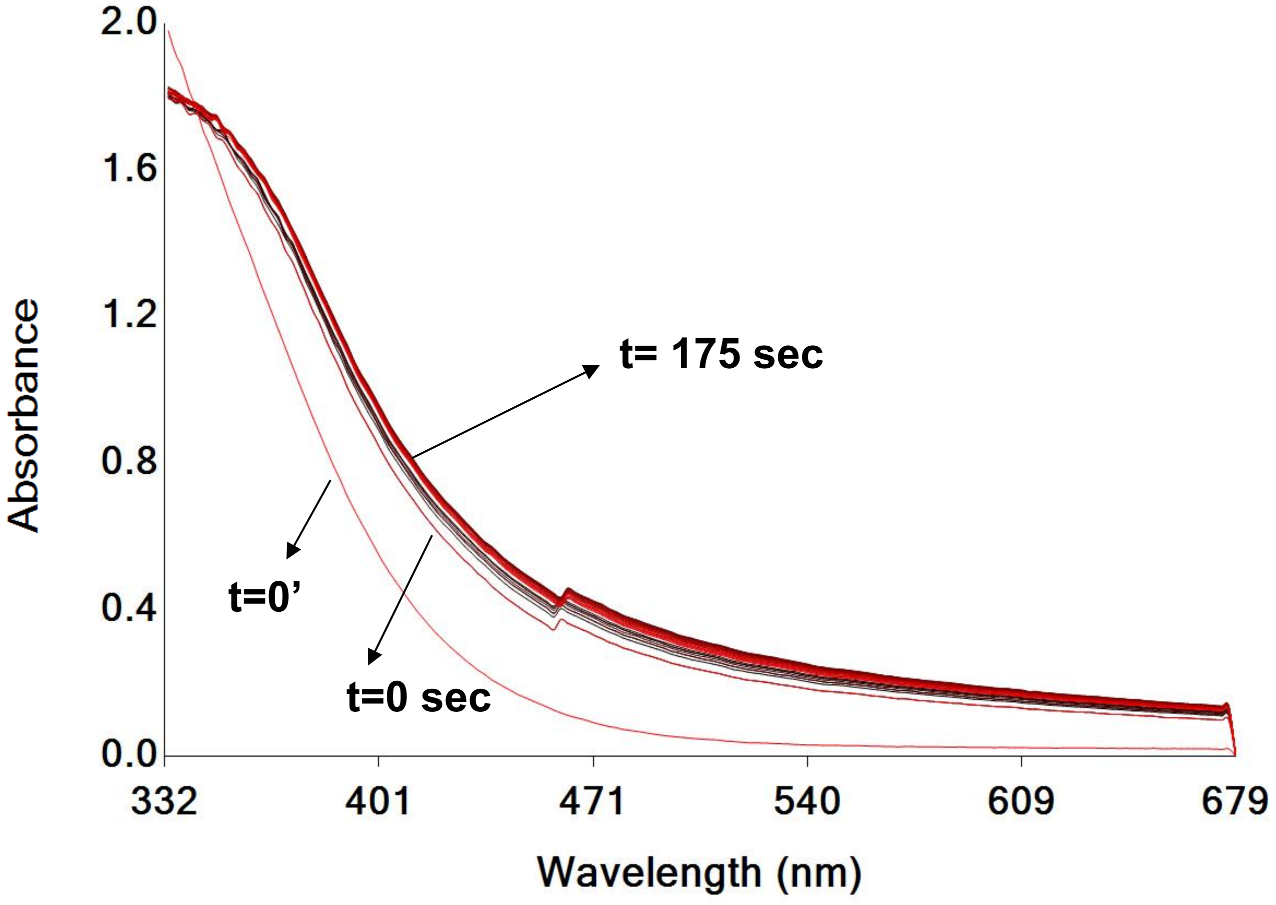

Figure S34: UV-spectra of **2** (1.00 mM, pH=9.0) without $[UO_2]^{2+}$ (t=0') and its reaction with 2.00 mM of $[UO_2]^{2+}$ *vs* time (measurement every 7 seconds for 175 seconds)

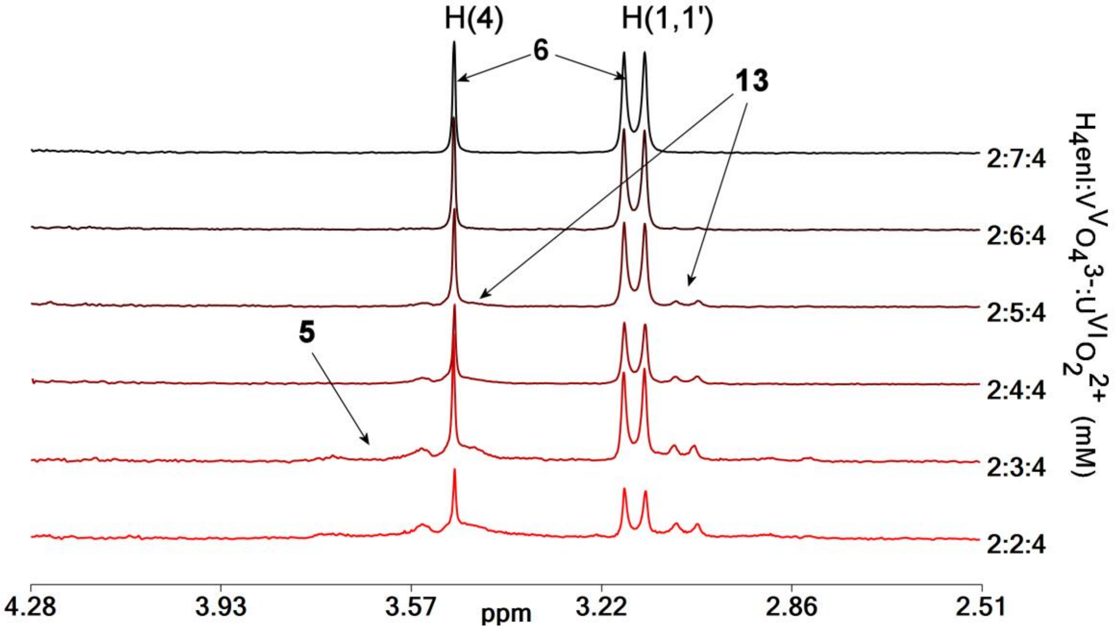

Figure S35. ^{1}H NMR spectra of *trans*-$[U^{VI}O_2]^{2+}$ in solution (D_2O, 2.00 mM), H_4enl (4.00 mM) and $V^VO_4^{3-}$ (2 – 7 mM) at pD = 10.0. The numbering for the proton peaks as shown in Scheme 3.

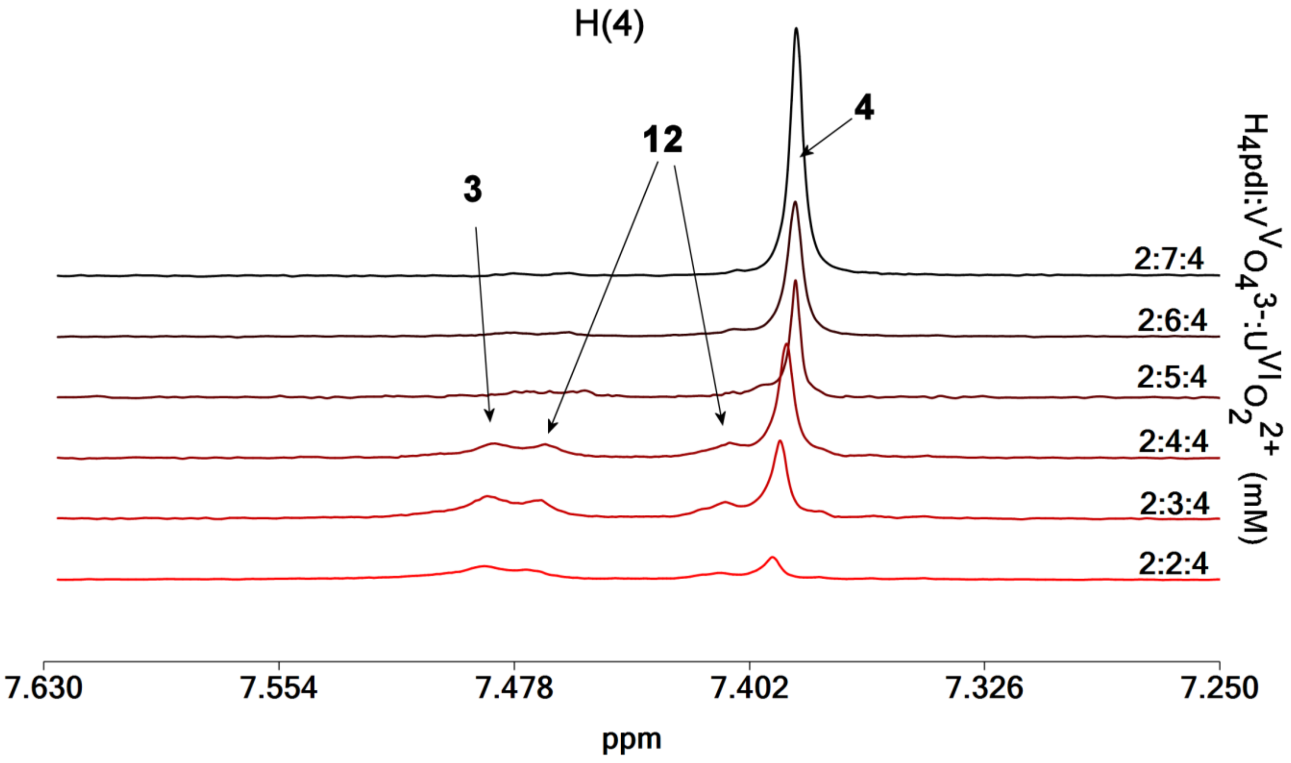

Figure S36. ^{1}H NMR spectra (aromatic region) of *trans*-$[U^{VI}O_2]^{2+}$ in solution (D_2O, 2.00 mM), H_4pdl (4.00 mM) and $V^{V}O_4^{3-}$ (2 – 7 mM) at pD = 10.0. The numbering for the proton peaks as shown in Scheme 3.

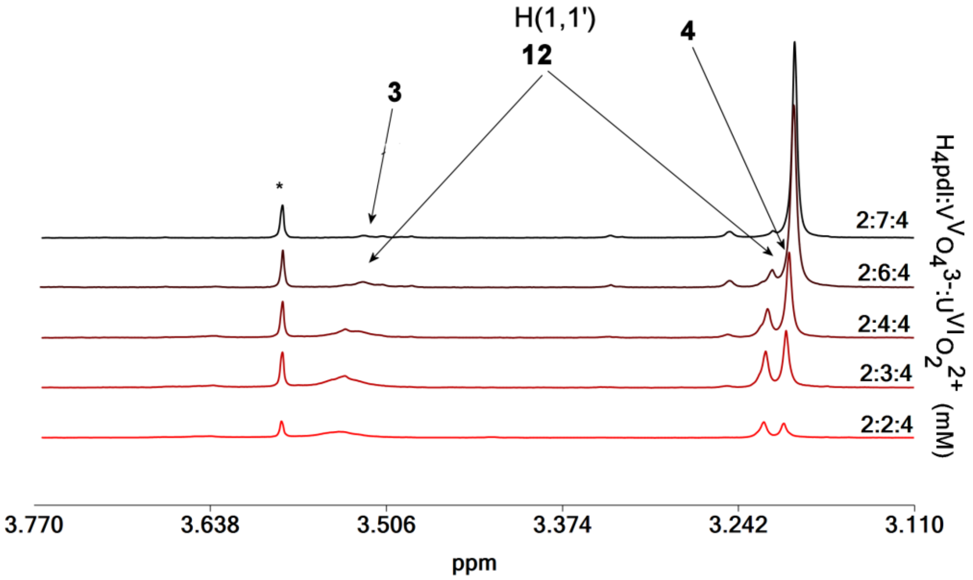

Figure S37. ^{1}H NMR spectra (aliphatic region) of *trans*-$[U^{VI}O_2]^{2+}$ in solution (D_2O, 2.00 mM), H_4pdl (4.00 mM) and $V^{V}O_4^{3-}$ (2 – 7 mM) at pD = 10.0. The numbering for the proton peaks as shown in Scheme 3.

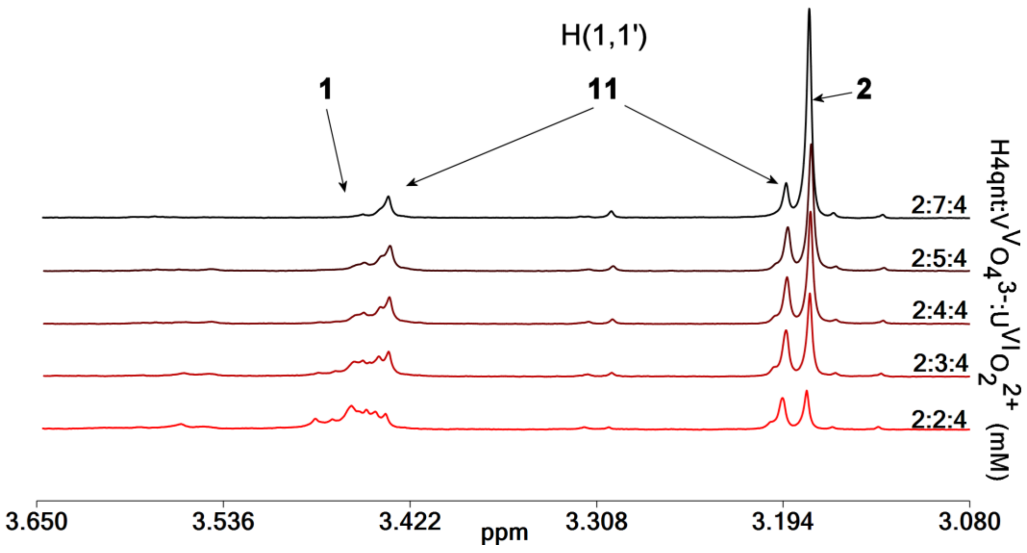

Figure S38. ^{1}H NMR spectra of *trans*-$[U^{VI}O_2]^{2+}$ in solution (D_2O, 2.00 mM), H_4qtn (4.00 mM) and $V^{V}O_4^{3-}$ (2 – 7 mM) at pD = 10.0. The numbering for the proton peaks as shown in Scheme 3.

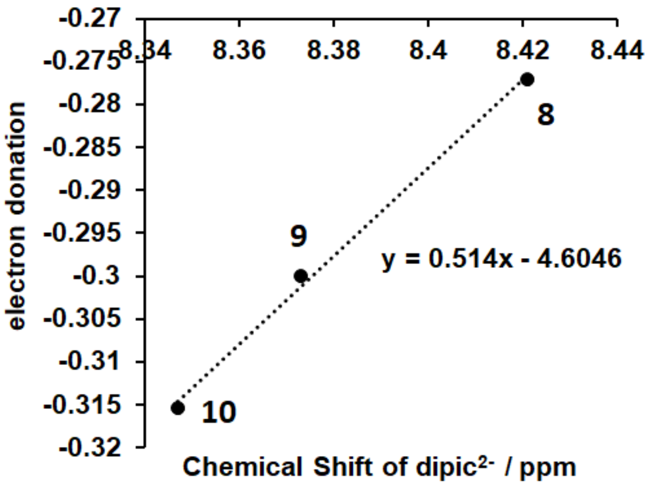

Figure S39. Correlation of the ^{1}H NMR chemical shifts of the protons of the dipic^{2-} in complexes **8-10** *vs* FMO Mulliken populations.

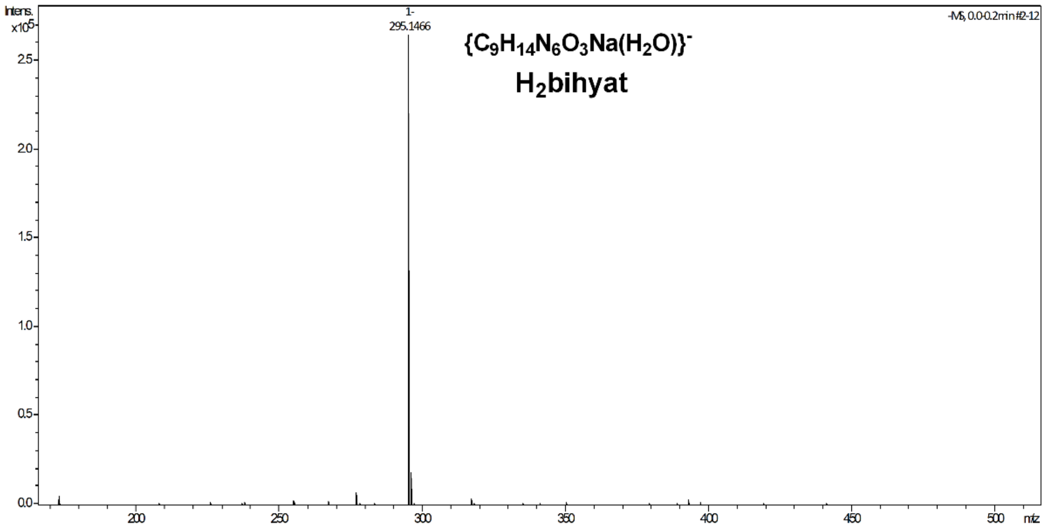

Figure S40. ESI-MS of ligand **H2bihyat** in MeOH in the presence of 4 drops of aqueous solution of base (NaOH 1M).

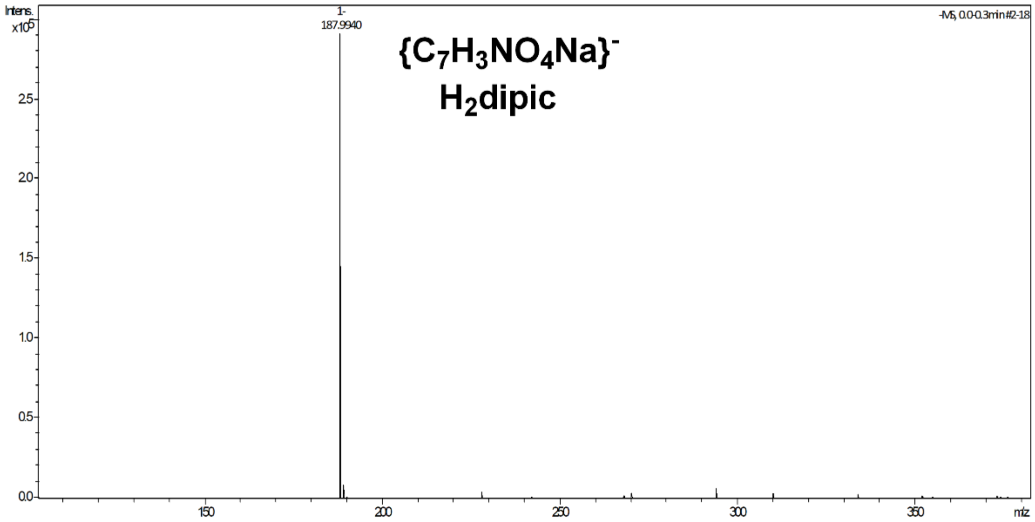

Figure S41. ESI-MS of ligand **H2dipic** in MeOH in the presence of 4 drops of aqueous solution of base (NaOH 1M).

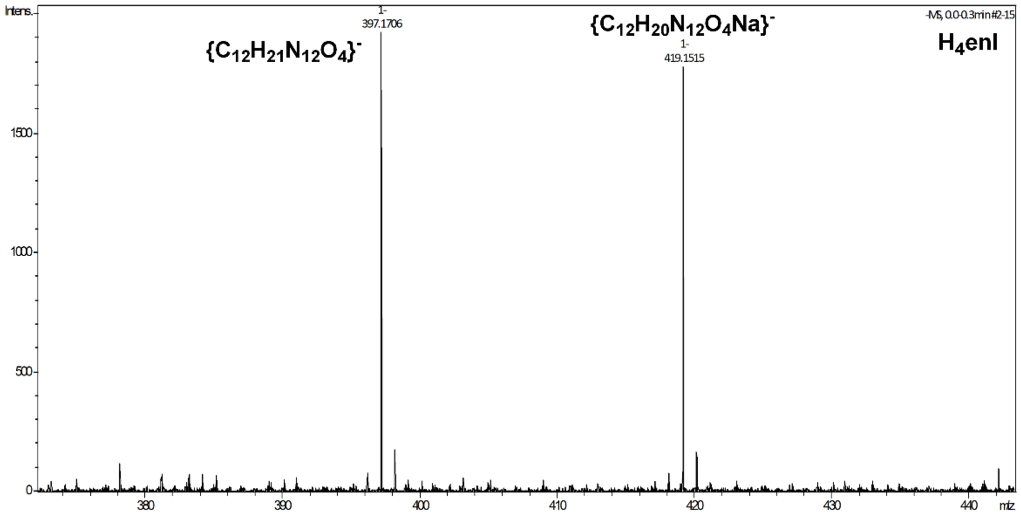

Figure S42. ESI-MS of ligand **H4enl** in MeOH in the presence of 4 drops of aqueous solution of base (NaOH 1M).

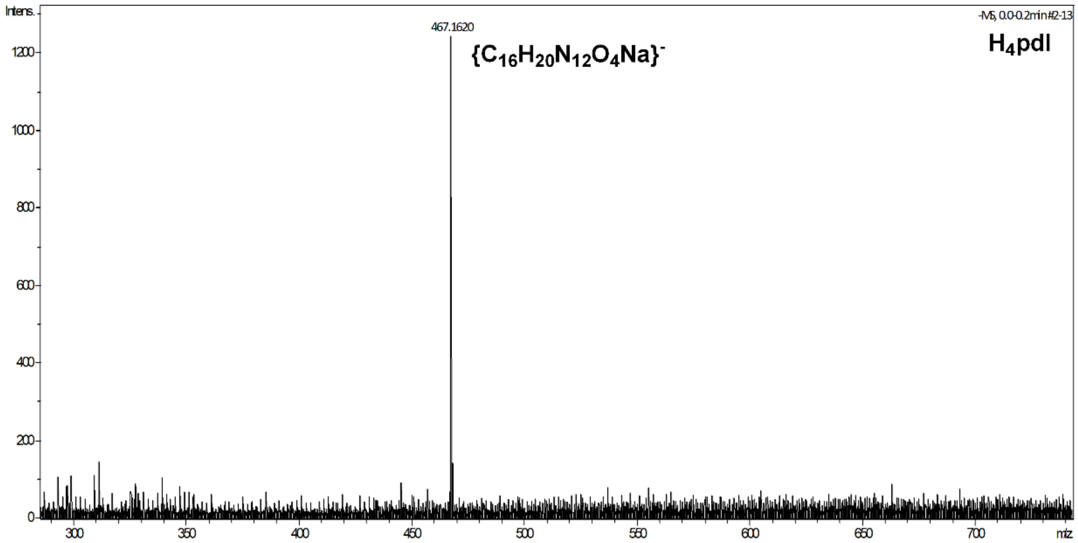

Figure S43. ESI-MS of ligand **H4pdl** in MeOH in the presence of 4 drops of aqueous solution of base (NaOH 1M).

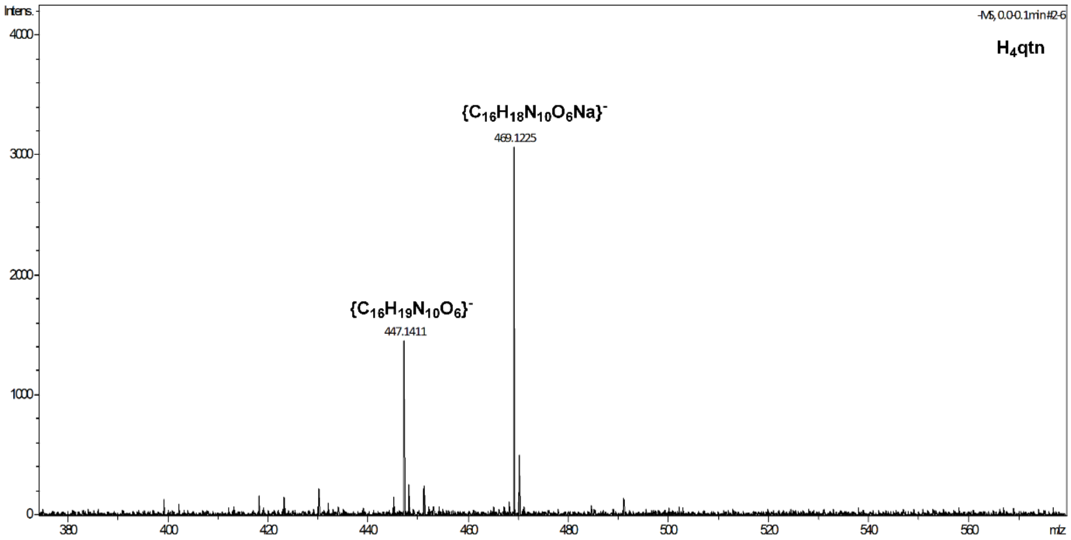

Figure S44. ESI-MS of ligand **H_4qtn** in MeOH in the presence of 4 drops of aqueous solution of base (NaOH 1M).

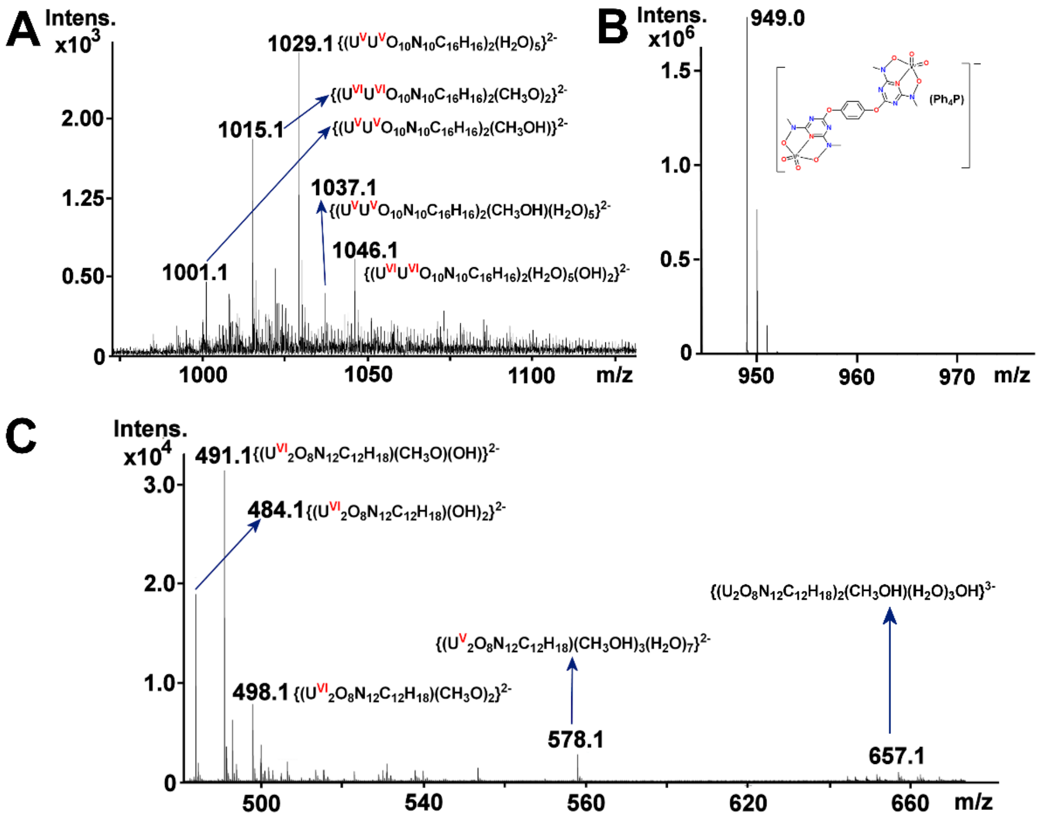

Figure S45. ESI MS of solutions (H_2O:CH_3OH; 1:1 *v/v*) at pH=9.0 of A) H_4qtn (2 mM) : *trans*-$U^{VI}O_2^{2+}$ (4 mM), B) H_4qtn (2 mM) : *cis*-$V^VO_2^+$ (4 mM), C) H_4enl (2 mM) : *trans*-$U^{VI}O_2^{2+}$ (4 mM).

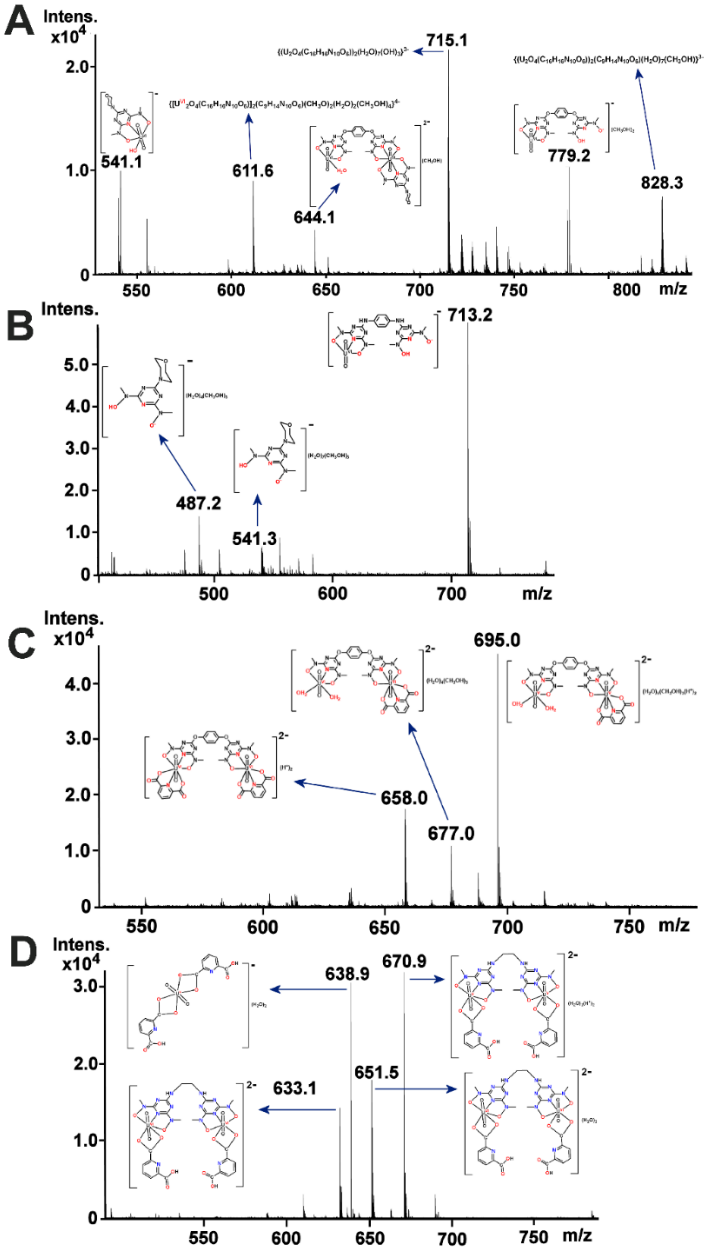

Figure S46. ESI MS of solutions (H_2O:CH_3OH; 1:1 *v/v*) at pH=9.0 of A) H_4qtn (2 mM) : H_2bihyat (4 mM) : *trans*-$U^{VI}O_2^{2+}$ (4 mM), B) H_4pdl (2 mM) : H_2bihyat (4 mM) : *trans*-$U^{VI}O_2^{2+}$ (4 mM), C) H_4qtn (2 mM) : H_2dipic (4 mM) : *trans*-$U^{VI}O_2^{2+}$ (4 mM), D) H_4enl (2 mM) : H_2dipic (4 mM) : *trans*-$U^{VI}O_2^{2+}$ (4 mM).

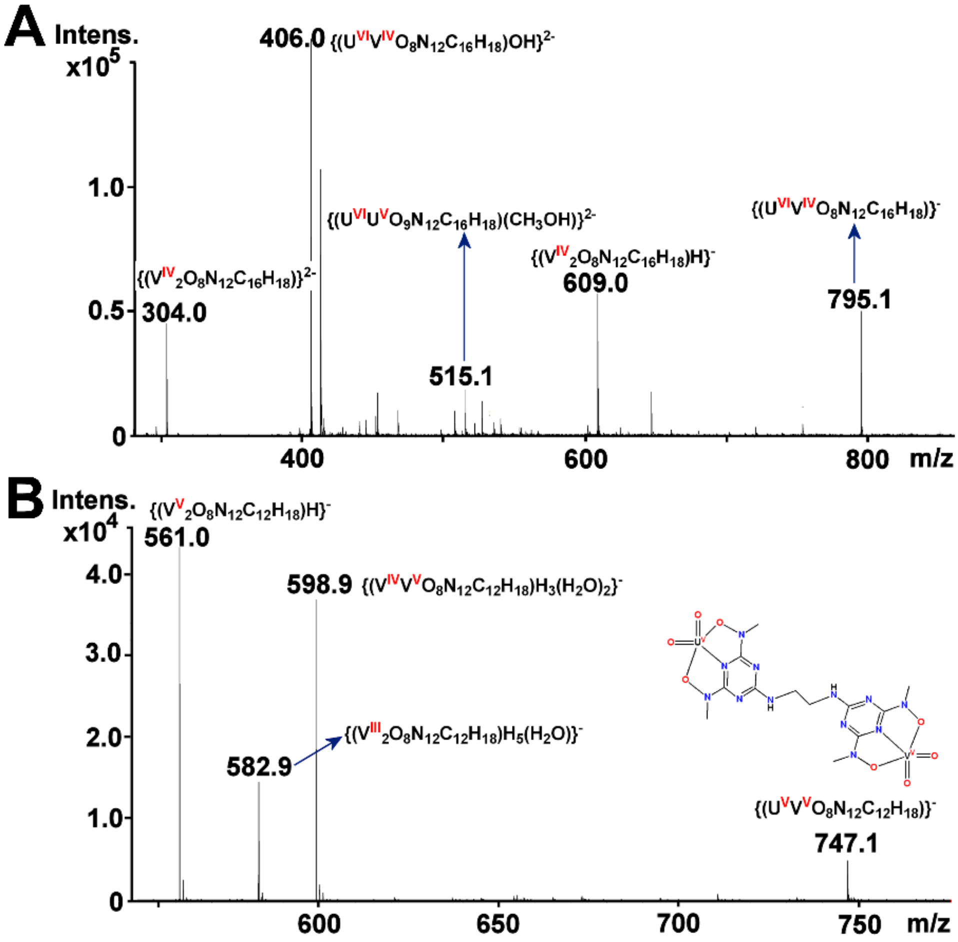

Figure S47. ESI-MS of solutions (H_2O:CH_3OH; 1:1 *v/v*) at pH=9.0 of A) H_4qtn (2 mM) : *cis*-$V^VO_2^+$ (4 mM): *trans*-$U^{VI}O_2^{2+}$ (4 mM), B) H_4enl (2 mM) : *cis*-$V^VO_2^+$ (4 mM): *trans*-$U^{VI}O_2^{2+}$ (4 mM).

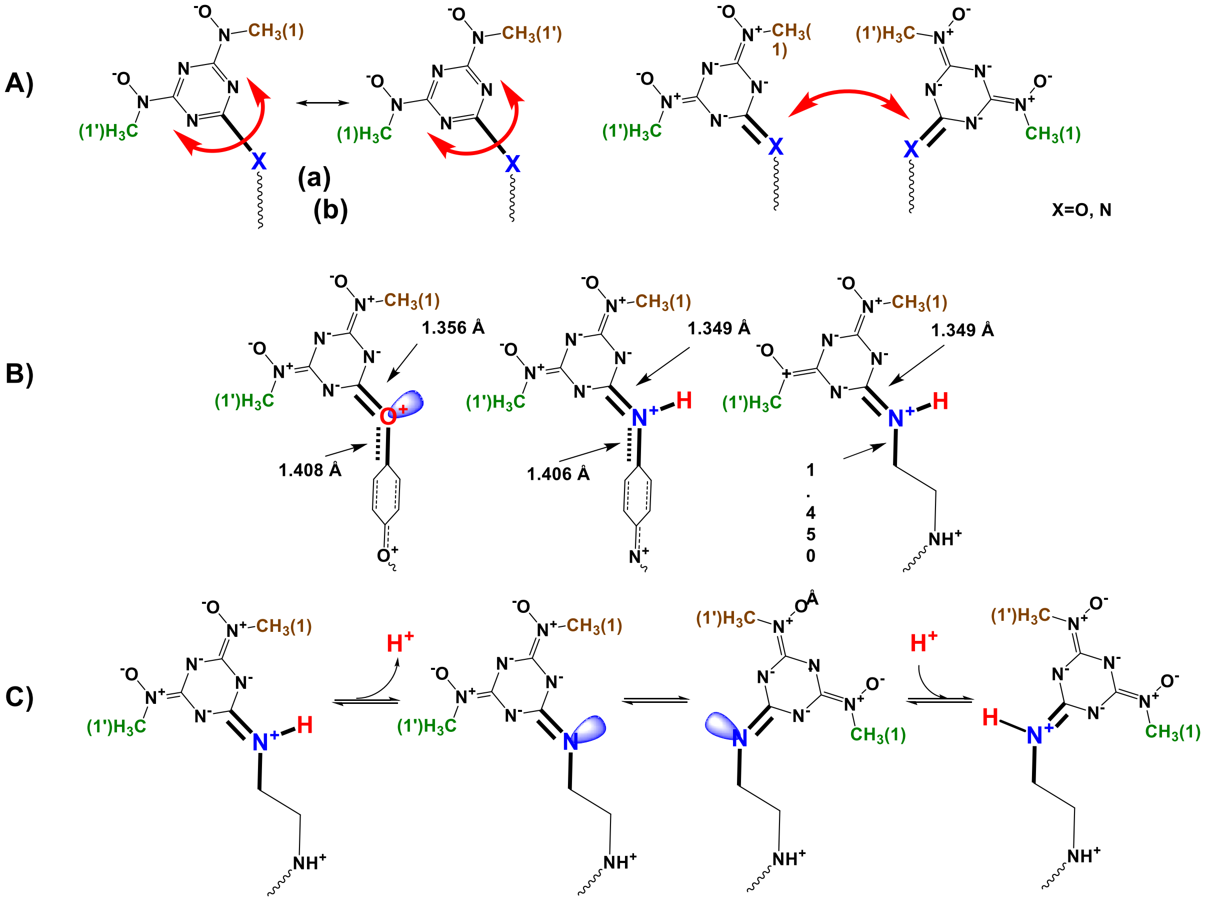

Scheme 6. The chemdraw structures in the scheme show the chemical inequivalence of the two methyl groups of the hydroxylamine of the half part of the BL and depict: A) Exchange mechanisms between the hydroxylamine methyl groups (a) resonance form A (Scheme 5) rotation around C(5)-X bond, B) resonance form B (Scheme 5) flip of C(5)-X bond centred on X. Bond resonance of BLs in metal complexes based on the crystal structure.

Dynamic Investigation of the Fluxional Behaviour of the Complexes by ^{1}H NMR

Careful inspection of the structures of the BLs reveal that the hydroxylamine methyl protons have a different chemical environment and therefore two peaks are expected for these protons in the ^{1}H NMR spectra of free and the ligated BLs to metal ions.

However, free BLs give only one signal for both H(1) and H(1'). This is attributed to the fast exchange between the hydroxylamine methyl groups through either rotation of the triazine around the C(5)-X bond (X=N or O), when BL is in resonance form A (Scheme 6A(a)), or flip of the triazine around atom X when BL is in resonance form B (Scheme 6A(b)). The pathway (a) in Scheme 6A is more facile than pathway (b). Although free BLs can easily acquire resonance form A allowing the fast rotation of the triazine ring, in the complexes, the resonance structure of BL has been locked to form B due to the ligation on the metal ion, as it is evident from the crystal structures of **1**-**6** (Scheme 6B).

The hydroxylamine methyl protons give in ^{1}H NMR spectra of the solutions of hydroquinone and 1,4-phenylenediamine metal complexes **1-4** only one peak, and for ethylenediamine complexes two peaks, that are exchanged as it is evident from the 2D ^{18} EXSY and VT ^{1}H NMR spectroscopies (Figure S13, S14), in all pHs.

Apparently, the triazine moiety changes position towards hydroquinone very fast, in **1** and **2**, resulting in the fast exchange of H(1) with H(1'). The only obstacle for the change of the triazine direction towards the hydroquinone through the flipping mechanism b (Scheme 6A(b)) is the free electron pair on the sp^2 hydroquinone oxygen atom, [O(5)] (Scheme 6A), and the exchange mechanism is through a mechanism similar to the inverse umbrella of amines. In the case of the 1,4-phenylenediamine and ethylenediamine complexes **3** – **6** the exchange mechanism proceeds first through deprotonation of the N(6)-H (Scheme 6C). The N(6)-H is more acidic for 1,4-phenylenediamine than for ethylenediamine complexes resulting in faster exchange reaction rates for the former. In addition, increasing the pH the exchange rates become larger supporting the proposed mechanism (Scheme 6C). The exchange rates for complex **6** were calculated by simulating the spectra at various temperatures. The activation

enthalpy and entropy were calculated from the Eyring plot ($\Delta S^{\#}$=197 J/mol, $\Delta H^{\#}$=127 kJ/mol/K; Figure S14). The large positive activation entropy is in line with the proton dissociation first step mechanism.

structures of **2**, **4** and **6**, C) Exchange mechanism between the hydroxylamine methyl groups for complexes **5** and **6**

Table S1. Crystal Data and Structure Refinement for the compounds **1** and **2**.

parameter	**1 (U)**	**2 (V)**
Empirical formula	$C_{20}H_{44}N_{10}O_{20}U$	$C_{64}H_{64}N_{10}O_{14}P_2V_2$
Formula weight	1220.71	1361.07
Temperature / K	100	100
Wavelength / Å	0.71073	0.71073
Space group	C 2/c	P -1
a / Å	19.1983(12)	9.6601(6)
b / Å	10.8769(5)	13.0810(8)
c / Å	20.1672(13)	14.2532(9)
α / °	90	64.133(6)
β / °	112.320(8)	83.629(5)
γ / °	90	73.951(5)
Volume / $Å^3$	3894.8(5)	1557.38(19)
Z	4	1
Density / Mg/m^3	2.081	1.450
Absorption coefficient mm^{-1}	8.389	0.426
R1[a]	0.0316	0.0421
wR2[b]	0.0697	0.1049
GoF, S[c]	1.037	1.052

[a] $R1 = \Sigma||F_o| - |F_c||/\Sigma|F_o|$. [b] $wR2 = \{\Sigma[w(F_o^2 - F_c^2)^2]/\Sigma[w(F_o^2)^2]\}^{1/2}$, where $w = 1/[\sigma^2(F_o^2) + (aP)^2 + bP]$, $P = (F_o^2 + 2F_c^2)/3$. [c] GoF = $\{\Sigma[w(F_o^2 - F_c^2)^2]/(n - p)\}^{1/2}$, where n = number of reflections and p is the total number of parameters refined.

Table S2. Crystal Data and Structure Refinement for the compounds **3, 4** and **6**.

parameter	**3**	**4**	**6**
Empirical formula	$C_{19}H_{41}N_{12}O_{19.32}U_2$	$C_{64}H_{58}N_{12}O_8P_2V_2$	$C_{60}H_{58}N_{12}O_{15}P_2V_2$
Formula weight	1222.82	1287.04	1350.68
Temperature / K	100	100	100
Wavelength / Å	0.71073	0.71073	0.71073
Space group	P-1	$P2_1/c$	$P2_1/n$
a / Å	7.2459(3)	12.8928(4)	9.3947(2)
b / Å	10.7394(3)	15.1819(4)	10.0908(2)
c / Å	13.0638(4))	16.2879(5)	34.2649(5)
α / °	69.157(3)	90	90
β / °	85.966(3)	109.198(4)	96.475(2)
γ / °	83.758(3)	90	90
Volume / Å^3	943.87(6)	3010.85(17)	3227.59(11)
Z	1	2	2
Density / Mg/m^3	2.151	1.420	1.390
Absorption coefficient mm^{-1}	24.758	3.657	3.521
R1[a]	0.0365	0.0559	0.0636
wR2[b]	0.0953	0.1316	0.1553
GoF, S[c]	1.111	1.039	1.061
R-Factor (%)	3.65	4.65	6.14

[a] $R1 = \Sigma||F_o| - |F_c||/\Sigma|F_o|$. [b] $wR2 = \{\Sigma[w(F_o^2 - F_c^2)^2]/\Sigma[w(F_o^2)^2]\}^{1/2}$, where $w = 1/[\sigma^2(F_o^2) + (aP)^2 + bP]$, $P = (F_o^2 + 2F_c^2)/3$. [c] $GoF = \{\Sigma[w(F_o^2 - F_c^2)^2]/(n - p)\}^{1/2}$, where n = number of reflections and p is the total number of parameters refined.

Table S3. Interatomic Distances (Å) and Angles (deg) Relevant to the U^{VI} and V^{V} Coordination Sphere.

parameter	**1**	**2**
M[a](1) - N(3)	2.435(4)	1.997(2)
M(1) - O(1)	1.772(4)	1.628(2)
M(1) - O(2)	1.771(4)	1.633(2)
M(1) - O(3)	2.421(3)	1.998(2)
M(1) - O(4)	2.392(3)	1.987(1)
M(1) - O(6)	2.379(3)	-
M(1) - O(7)	2.366(3)	-
N(3)- M(1) - O(1)	92.41(2)	133.50(8)
N(3)- M(1) - O(2)	90.59(2)	115.32(8)
N(3)- M(1) - O(3)	62.94(1)	74.13(7)
N(3)- M(1) - O(4)	63.85(1)	74.18(7)
N(3)- M(1) - O(6)	139.72(1)	-
N(3)- M(1) - O(7)	135.17(1)	-
O(1) - M(1) - O(2)	176.58(2)	111.15(9)
O(1) - M(1) - O(3)	91.63(1)	96.04(8)
O(1) - M(1) - O(4)	92.33(1)	97.69(8)
O(1) - M(1) - O(6)	90.01(1)	-
O(1) - M(1) - O(7)	90.24(1)	-
O(2) - M(1) - O(3)	88.21(1)	100.60(8)
O(2) - M(1) - O(4)	90.51(1)	102.71(8)
O(2) - M(1) - O(6)	86.63(1)	-

O(2) - M(1) - O(7)	88.80(1)	-
O(3) - M(1) - O(4)	126.66(1)	146.42(7)
O(3) - M(1) - O(6)	75.88(1)	-
O(3) - M(1) - O(7)	160.88(1)	-
O(4) - M(1) - O(6)	157.21(1)	-
O(4) - M(1) - O(7)	72.24(1)	-
O(6) - M(1) - O(7)	85.10(1)	-

[a]M corresponds to U(1) and V(1) for **1** and **2** respectively.

Table S4. Interatomic Distances (Å) and Angles (deg) Relevant to the U^{VI} and V^{V} Coordination Sphere.

parameter	**3**	**4**	**6**
M[a](1) - N(3)	2.441(6)	2.005(2)	1.992(3)
M(1) - O(1)	1.778(5)	1.634(2)	1.654(2)
M(1) - O(2)	1.774(5)	1.627(3)	1.632(3)
M(1) - O(3)	2.362(5)	1.986(2)	1.970(2)
M(1) - O(4)	2.389(6)	1.986(2)	1.985(2)
M(1) - O(6)	2.379(6)	-	-
M(1) - O(7)	2.371(5)	-	-
N(3)- M(1) - O(1)	93.75(2)	132.01(1)	133.29(1)
N(3)- M(1) - O(2)	90.67(2)	116.87(9)	116.12(1)
N(3)- M(1) - O(3)	62.91(2)	74.45(8)	74.65(1)
N(3)- M(1) - O(4)	63.07(2)	74.13(8)	74.40(9)
N(3)- M(1) - O(6)	143.07(2)	-	-
N(3)- M(1) - O(7)	134.90(2)	-	-
O(1) - M(1) - O(2)	175.57(3)	111.12(1)	110.58(1)
O(1) - M(1) - O(3)	88.69(2)	99.45(9)	96.87(1)
O(1) - M(1) - O(4)	89.80(2)	99.84(9)	95.10(1)
O(1) - M(1) - O(6)	87.76(2)	-	-
O(1) - M(1) - O(7)	89.69(2)	-	-
O(2) - M(1) - O(3)	93.62(2)	98.75(1)	102.11(1)
O(2) - M(1) - O(4)	91.91(2)	97.76(1)	102.66(1)
O(2) - M(1) - O(6)	88.89(2)	-	-

O(2) - M(1) - O(7)	86.95(2)	-	-
O(3) - M(1) - O(4)	125.72(2)	148.09(8)	146.54(1)
O(3) - M(1) - O(6)	80.27(2)	-	-
O(3) - M(1) - O(7)	162.19(2)	-	-
O(4) - M(1) - O(6)	153.84(2)	-	-
O(4) - M(1) - O(7)	72.00(2)	-	-
O(6) - M(1) - O(7)	81.95(2)	-	-

[a]M corresponds to U(1) for **3** and V(1) for **4** and **6**.

Table S5. DFT Optimized Structures.

$[VO_2(bihyat)]^-$

	Atom	X	Y	Z
1	C	-0.0526	3.5706	2.2566
2	H	0.4902	4.2544	1.5912
3	H	0.3273	3.6699	3.2799
4	H	-1.1302	3.8002	2.2334
5	C	1.0999	1.2484	6.6667
6	H	0.8039	2.1111	6.0605
7	H	2.1829	1.3117	6.8846
8	C	1.0678	-1.1986	6.6883
9	H	2.1487	-1.2866	6.9080
10	H	0.7494	-2.0635	6.0967
11	C	0.2826	-1.1280	7.9919
12	H	-0.8021	-1.1199	7.7699
13	H	0.5213	-1.9931	8.6264
14	C	0.3138	1.2215	7.9715
15	H	0.5759	2.0908	8.5907
16	H	-0.7708	1.2385	7.7496
17	V	-0.1277	-0.0181	-0.0202
18	O	-0.2930	1.9019	0.5392
19	N	0.4840	-1.2109	3.9921
20	N	0.5154	1.2284	3.9710
21	N	0.0552	-0.0027	1.9912
22	O	0.6269	0.0491	8.7529
23	O	-0.3413	-1.9212	0.5722
24	N	0.8350	0.0215	5.9191
25	C	0.6074	0.0128	4.5789
26	N	0.1760	2.2074	1.8332
27	O	1.3448	-0.0435	-0.7856
28	N	0.1255	-2.2174	1.8700
29	C	0.2198	-1.1504	2.6816
30	C	0.2477	1.1523	2.6618
31	O	-1.3868	-0.0115	-1.1087
32	C	-0.1399	-3.5666	2.3169
33	H	0.3742	-4.2756	1.6553
34	H	-1.2240	-3.7644	2.3104
35	H	0.2499	-3.6624	3.3368

$[UO_2(H_2O)_2(bihyat)]$

	Atom	X	Y	Z
1	U	-0.0026	0.0557	0.0969
2	O	-1.6510	-1.9441	-0.0957
3	O	2.0671	1.0417	0.1589
4	N	0.1106	6.5107	0.0111
5	O	-2.0139	1.1149	0.3832
6	O	0.0935	-0.3254	1.8863
7	O	0.2582	8.3654	2.1599
8	N	2.2892	2.3959	-0.0886
9	N	-2.2132	2.4751	0.1475
10	C	3.6442	2.8454	0.1606
11	H	4.3285	2.3177	-0.5162
12	H	3.6853	3.9228	-0.0338
13	H	3.9235	2.6422	1.2075
14	C	0.0875	5.1450	0.0536
15	N	1.2858	4.5131	-0.0106
16	O	1.5781	-1.9786	-0.2849
17	N	-1.1333	4.5561	0.1106
18	C	1.1977	3.1796	0.0556
19	N	0.0495	2.4799	0.2382
20	C	-1.0706	7.2850	0.4077
21	H	-1.9762	6.7008	0.2099
22	H	-1.0870	8.2082	-0.1939
23	C	-3.5198	2.9732	0.5288
24	H	-4.2861	2.4699	-0.0749
25	H	-3.7014	2.7808	1.5989
26	H	-3.5409	4.0513	0.3345
27	C	1.4075	7.5804	1.7695
28	H	1.4380	6.6437	2.3583
29	H	2.2932	8.1894	2.0022
30	C	-1.0857	3.2202	0.1740
31	C	-0.9563	7.6294	1.8899
32	H	-1.7875	8.2747	2.2089
33	H	-0.9655	6.6946	2.4824
34	C	1.3554	7.2386	0.2836
35	H	1.3430	8.1635	-0.3154
36	H	2.2127	6.6227	-0.0085
37	O	-0.1030	0.2407	-1.7192
38	H	2.1682	-1.8667	-1.0642
39	H	2.1972	-2.1456	0.4609
40	H	-2.2933	-1.8533	-0.8351
41	H	-2.2159	-2.0819	0.6975

bihyat^{2-}

	Atom	X	Y	Z
1	O	3.1574	-1.1419	2.3958
2	N	-1.6741	1.2927	-0.0000
3	O	3.1574	-1.1419	-2.3958
4	O	-4.0390	-0.3411	-0.0000
5	N	1.9245	-0.5479	2.3162
6	N	1.9245	-0.5479	-2.3162
7	C	1.2968	-0.2770	3.5968
8	H	1.9208	0.4312	4.1705
9	H	0.2953	0.1406	3.4489
10	H	1.2327	-1.2200	4.1669
11	C	-0.4333	0.6196	-0.0000
12	N	0.0951	0.3613	1.2030
13	N	0.0951	0.3613	-1.2030
14	C	1.3311	-0.2477	1.1409
15	N	1.9984	-0.5785	-0.0000
16	C	-2.5033	1.1411	-1.2052
17	H	-1.8669	1.2239	-2.0945
18	H	-3.2477	1.9564	-1.2071
19	C	1.2968	-0.2770	-3.5968
20	H	1.9208	0.4312	-4.1705
21	H	1.2327	-1.2200	-4.1669
22	H	0.2953	0.1406	-3.4489
23	C	-3.2161	-0.2051	1.1847
24	H	-2.4615	-1.0152	1.2090
25	H	-3.8964	-0.3113	2.0437
26	C	1.3311	-0.2477	-1.1409
27	C	-3.2161	-0.2051	-1.1847
28	H	-3.8964	-0.3113	-2.0437
29	H	-2.4615	-1.0152	-1.2090
30	C	-2.5033	1.1411	1.2052
31	H	-3.2477	1.9564	1.2071
32	H	-1.8669	1.2239	2.0945

qtn^{4-}

	Atom	X	Y	Z
1	O	-3.9080	-0.1851	-1.6763
2	O	-2.5898	1.0635	3.9281
3	N	-5.0274	0.5306	0.7061
4	N	-3.1501	0.0118	-0.5592
5	N	-2.7842	0.6036	1.6611
6	O	-7.6831	0.9617	1.0537
7	N	-4.6878	0.9848	3.0822
8	N	-6.8044	1.0685	2.0929
9	C	-3.6771	0.3831	0.6210
10	C	-1.7217	-0.1922	-0.7380
11	H	-1.5677	-1.1921	-1.1754
12	H	-1.3258	0.5606	-1.4407
13	H	-1.2038	-0.1172	0.2222
14	C	-3.3995	0.8545	2.7976
15	C	-5.4825	0.8608	1.9464
16	C	-1.2762	0.5975	3.8485
17	C	-0.9951	-0.7449	3.5828
18	H	-1.8075	-1.4304	3.3431
19	C	-7.4133	1.4135	3.3659
20	H	-7.9334	2.3807	3.2611
21	H	-8.1602	0.6438	3.6238
22	H	-6.6560	1.4795	4.1540
23	C	-0.2528	1.4938	4.1470
24	H	-0.4961	2.5383	4.3495
25	O	3.8296	0.0949	-1.6265
26	O	2.6621	-0.7616	4.0808
27	N	4.9886	-0.6140	0.7396
28	N	3.1132	0.0366	-0.4671
29	N	2.8131	-0.3363	1.8088
30	O	7.6134	-1.2525	0.9947
31	N	4.7003	-1.0305	3.1306
32	N	6.7667	-1.2809	2.0665
33	C	3.6618	-0.3130	0.7104
34	C	1.6978	0.3409	-0.6022
35	H	1.5897	1.3492	-1.0355
36	H	1.2406	-0.3855	-1.2940
37	H	1.2029	0.2934	0.3725
38	C	3.4347	-0.6986	2.9118
39	C	5.4607	-0.9684	1.9674
40	C	1.3465	-0.2959	3.9423
41	C	1.0695	1.0420	4.1989
42	H	1.8856	1.7261	4.4364

43	C	7.3876	-1.6944	3.3123
44	H	7.8056	-2.7072	3.1826
45	H	8.2161	-1.0040	3.5436
46	H	6.6594	-1.6927	4.1303
47	C	0.3248	-1.1906	3.6276
48	H	0.5694	-2.2344	3.4249

$[(VO_2)_2qtn]^{2-}$

	Atom	X	Y	Z
1	V	-6.1797	0.1492	-0.7386
2	O	-4.2965	-0.1543	-1.4184
3	O	-2.6517	0.8249	4.0588
4	N	-4.9561	0.4763	0.8363
5	N	-3.2952	-0.2329	-0.4468
6	N	-2.7926	0.2918	1.8130
7	O	-7.3116	1.0368	0.6770
8	N	-4.6960	0.9973	3.1458
9	N	-6.7557	1.1114	1.9594
10	C	-3.6412	0.1828	0.7662
11	C	-1.9515	-0.4746	-0.9159
12	H	-1.9242	-1.4239	-1.4673
13	H	-1.6265	0.3498	-1.5684
14	H	-1.3045	-0.5351	-0.0339
15	C	-3.4112	0.6833	2.9254
16	C	-5.4481	0.8778	2.0283
17	C	-1.3071	0.4151	3.9751
18	C	-0.9927	-0.9362	3.8723
19	H	-1.7880	-1.6758	3.7834
20	C	-7.5766	1.7301	2.9718
21	H	-7.8218	2.7611	2.6744
22	H	-8.4989	1.1478	3.1003
23	H	-7.0063	1.7371	3.9071
24	C	-0.3237	1.3912	4.0905
25	H	-0.6080	2.4404	4.1750
26	O	-6.8185	-1.3786	-0.7891
27	O	-6.7080	1.0666	-2.0186
28	O	4.2732	0.2000	-1.4010
29	O	2.6764	-0.7544	4.0928
30	N	4.9429	-0.4653	0.8394
31	N	3.2889	0.3102	-0.4146
32	N	2.8034	-0.2057	1.8518
33	O	7.2750	-1.1059	0.6396
34	N	4.6993	-0.9980	3.1474
35	N	6.7368	-1.1699	1.9302
36	C	3.6384	-0.1255	0.7912
37	C	1.9432	0.5690	-0.8697
38	H	1.9218	1.5165	-1.4241
39	H	1.6014	-0.2537	-1.5158
40	H	1.3059	0.6401	0.0187
41	C	3.4239	-0.6342	2.9502
42	C	5.4391	-0.8922	2.0208

43	C	1.3308	-0.3437	3.9993
44	C	1.0179	1.0054	4.1072
45	H	1.8146	1.7436	4.1996
46	C	7.5502	-1.8275	2.9237
47	H	7.7556	-2.8626	2.6114
48	H	8.4937	-1.2785	3.0451
49	H	6.9933	-1.8259	3.8671
50	C	0.3480	-1.3199	3.8777
51	H	0.6325	-2.3694	3.7970
52	V	6.1548	-0.1658	-0.7491
53	O	6.8382	1.3431	-0.7928
54	O	6.6360	-1.0851	-2.0463

{[$UO_2(H_2O)_2$]qtn(VO_2)}$^-$

	Atom	X	Y	Z
1	V	-6.0802	0.2155	-0.7980
2	O	-4.1852	-0.0775	-1.4471
3	O	-2.6417	0.8168	4.0750
4	N	-4.8876	0.5146	0.8056
5	N	-3.2025	-0.1722	-0.4583
6	N	-2.7401	0.3240	1.8163
7	O	-7.2407	1.0803	0.6142
8	N	-4.6669	1.0075	3.1257
9	N	-6.7035	1.1468	1.9039
10	C	-3.5707	0.2273	0.7543
11	C	-1.8487	-0.3901	-0.9090
12	H	-1.8037	-1.3227	-1.4868
13	H	-1.5184	0.4553	-1.5311
14	H	-1.2181	-0.4714	-0.0173
15	C	-3.3788	0.6964	2.9241
16	C	-5.3997	0.9043	1.9937
17	C	-1.2977	0.4018	4.0170
18	C	-0.9834	-0.9447	3.8581
19	H	-1.7769	-1.6760	3.7088
20	C	-7.5465	1.7327	2.9176
21	H	-7.8093	2.7631	2.6339
22	H	-8.4584	1.1305	3.0285
23	H	-6.9848	1.7365	3.8582
24	C	-0.3165	1.3666	4.2127
25	H	-0.6011	2.4113	4.3418
26	O	-6.7211	-1.3095	-0.8895
27	O	-6.5828	1.1585	-2.0698
28	O	4.0241	0.2127	-1.3871
29	O	2.6832	-0.7761	4.1681
30	N	4.8813	-0.5550	0.8286
31	N	3.1293	0.2672	-0.3335
32	N	2.7723	-0.2414	1.9237
33	O	7.3046	-1.1120	0.7435
34	N	4.6899	-1.0075	3.1770
35	N	6.7096	-1.1789	1.9916
36	C	3.5783	-0.1855	0.8384
37	C	1.7498	0.5537	-0.6590
38	H	1.6924	1.5253	-1.1686
39	H	1.3497	-0.2351	-1.3138
40	H	1.1866	0.5883	0.2792
41	C	3.4094	-0.6624	3.0107
42	C	5.4001	-0.9199	2.0281

43	C	1.3359	-0.3670	4.0791
44	C	1.0233	0.9752	4.2487
45	H	1.8191	1.7045	4.4000
46	C	7.5022	-1.7626	3.0490
47	H	7.7668	-2.7986	2.7864
48	H	8.4171	-1.1695	3.1851
49	H	6.9082	-1.7496	3.9686
50	C	0.3554	-1.3331	3.8832
51	H	0.6402	-2.3777	3.7549
52	U	6.2758	-0.2864	-1.1535
53	O	6.6529	1.4305	-0.6666
54	O	6.0065	-1.9766	-1.7897
55	O	8.7310	-0.5169	-1.8920
56	H	9.0766	-1.4380	-1.8922
57	H	9.3750	-0.0149	-1.3431
58	O	6.1563	0.5067	-3.5989
59	H	5.5678	-0.0257	-4.1806
60	H	5.8494	1.4333	-3.7229

$[UO_2(H_2O)_2]_2qtn$

	Atom	X	Y	Z
1	U	-6.3163	0.3347	-1.1675
2	O	-4.0480	-0.0655	-1.4535
3	O	-2.6690	0.7742	4.1195
4	N	-4.9007	0.5979	0.7992
5	N	-3.1364	-0.1120	-0.4154
6	N	-2.7609	0.3467	1.8496
7	O	-7.3405	1.0784	0.7647
8	N	-4.6942	0.9556	3.1622
9	N	-6.7346	1.1050	2.0096
10	C	-3.5827	0.2854	0.7764
11	C	-1.7561	-0.3594	-0.7654
12	H	-1.6881	-1.3101	-1.3116
13	H	-1.3764	0.4599	-1.3944
14	H	-1.1829	-0.4194	0.1650
15	C	-3.4014	0.6796	2.9636
16	C	-5.4155	0.8939	2.0188
17	C	-1.3171	0.3880	4.0380
18	C	-0.9810	-0.9516	3.8665
19	H	-1.7634	-1.6967	3.7274
20	C	-7.5317	1.6179	3.1005
21	H	-7.8259	2.6576	2.8902
22	H	-8.4290	0.9946	3.2165
23	H	-6.9283	1.5766	4.0132
24	C	-0.3516	1.3711	4.2169
25	H	-0.6538	2.4100	4.3524
26	O	-6.6133	-1.4055	-0.7093
27	O	-6.1275	2.0465	-1.7749
28	O	4.0493	0.1619	-1.4224
29	O	2.6830	-0.7188	4.1462
30	N	4.8949	-0.5525	0.8140
31	N	3.1497	0.2443	-0.3744
32	N	2.7784	-0.2224	1.8914
33	O	7.3214	-1.1049	0.7549
34	N	4.6909	-0.9632	3.1684
35	N	6.7166	-1.1558	1.9985
36	C	3.5914	-0.1855	0.8096
37	C	1.7695	0.5024	-0.7193
38	H	1.7051	1.4530	-1.2660
39	H	1.3827	-0.3154	-1.3460
40	H	1.1995	0.5661	0.2129
41	C	3.4109	-0.6233	2.9892
42	C	5.4076	-0.8954	2.0227

43	C	1.3301	-0.3317	4.0553
44	C	0.9960	1.0048	4.2301
45	H	1.7801	1.7482	4.3734
46	C	7.5086	-1.6996	3.0774
47	H	7.7915	-2.7377	2.8444
48	H	8.4128	-1.0887	3.2064
49	H	6.9059	-1.6720	3.9910
50	C	0.3648	-1.3151	3.8690
51	H	0.6669	-2.3542	3.7351
52	U	6.3006	-0.3282	-1.1662
53	O	6.6709	1.4012	-0.7202
54	O	6.0380	-2.0341	-1.7628
55	O	8.7631	-0.5609	-1.8829
56	H	9.1195	-1.4773	-1.8495
57	H	9.3958	-0.0337	-1.3445
58	O	6.1990	0.4035	-3.6339
59	H	5.6178	-0.1447	-4.2081
60	H	5.8937	1.3263	-3.7871
61	O	-6.1361	-0.4488	-3.6177
62	H	-5.7186	-1.3327	-3.7293
63	H	-5.6068	0.1441	-4.1977
64	O	-8.7820	0.4938	-1.8872
65	H	-9.4052	-0.0548	-1.3591
66	H	-9.1643	1.3996	-1.8525

enl^{4-}

	Atom	X	Y	Z
1	O	0.2358	-4.6180	-5.4794
2	O	-2.4074	-5.5620	-1.6218
3	N	1.0598	-2.2447	-2.8728
4	N	0.7941	-3.5926	-4.7602
5	N	1.3372	-1.0496	-0.9114
6	H	1.7781	-0.3944	-1.5622
7	N	-0.5239	-4.1002	-2.9261
8	N	-0.2443	-2.7569	-0.9068
9	N	-1.7824	-4.4997	-1.0229
10	C	0.4341	-3.3116	-3.4895
11	C	0.6634	-2.0646	-1.5996
12	C	-0.8285	-3.7711	-1.6397
13	C	0.6970	-0.4135	0.2385
14	H	1.4251	0.2683	0.7037
15	H	0.4292	-1.1955	0.9652
16	C	1.8011	-2.8339	-5.4769
17	H	2.6152	-3.5137	-5.7836
18	H	2.2012	-2.0333	-4.8454
19	H	1.3497	-2.4041	-6.3884
20	C	-2.1833	-4.2559	0.3506
21	H	-3.2797	-4.1390	0.3812
22	H	-1.6968	-3.3529	0.7350
23	H	-1.9108	-5.1262	0.9741
24	O	-0.1180	4.5857	5.5805
25	O	2.5331	5.5324	1.7285
26	N	-0.9313	2.2096	2.9732
27	N	-0.6713	3.5579	4.8611
28	N	-1.2037	1.0148	1.0109
29	H	-1.6449	0.3586	1.6607
30	N	0.6490	4.0680	3.0293
31	N	0.3756	2.7240	1.0096
32	N	1.9110	4.4690	1.1288
33	C	-0.3085	3.2776	3.5911
34	C	-0.5323	2.0302	1.7007
35	C	0.9568	3.7392	1.7436
36	C	-0.5619	0.3805	-0.1389
37	H	-1.2891	-0.3012	-0.6054
38	H	-0.2939	1.1635	-0.8645
39	C	-1.6795	2.7983	5.5753
40	H	-2.5002	3.4748	5.8715
41	H	-2.0699	1.9909	4.9463
42	H	-1.2319	2.3777	6.4928

43	C	2.3147	4.2258	-0.2441
44	H	3.4113	4.1099	-0.2727
45	H	1.8297	3.3226	-0.6296
46	H	2.0427	5.0962	-0.8678

$[(VO_2)_2enl]^{2-}$

	Atom	X	Y	Z
1	V	-6.1565	-3.3094	0.3671
2	O	-6.3730	-2.3314	2.0975
3	O	-6.5427	-4.8205	0.9489
4	V	6.1565	3.3094	-0.3671
5	O	6.5427	4.8205	-0.9489
6	O	7.3804	2.6854	0.5641
7	O	-4.8333	-3.9333	-1.0117
8	O	-7.3804	-2.6854	-0.5641
9	N	-3.5920	-0.1417	1.5020
10	N	-5.6237	-1.1407	2.2337
11	N	-1.6669	0.6725	0.5455
12	H	-1.6428	1.2504	1.3860
13	N	-4.5834	-2.0502	0.4947
14	N	-2.6035	-1.1687	-0.4854
15	N	-3.8110	-3.0263	-1.3395
16	C	-4.5519	-1.0718	1.4226
17	C	-2.6559	-0.2579	0.5145
18	C	-3.6159	-2.0490	-0.4468
19	C	-0.4587	0.5627	-0.2542
20	H	0.0727	1.5221	-0.1970
21	H	-0.7347	0.3694	-1.3011
22	C	-5.6806	-0.5502	3.5530
23	H	-6.7208	-0.2866	3.7839
24	H	-5.0632	0.3552	3.5418
25	H	-5.2953	-1.2569	4.3062
26	C	-2.8255	-3.5150	-2.2742
27	H	-2.3696	-4.4409	-1.8894
28	H	-2.0589	-2.7393	-2.3832
29	H	-3.3018	-3.7090	-3.2448
30	O	6.3730	2.3314	-2.0975
31	O	4.8333	3.9333	1.0117
32	N	3.5920	0.1417	-1.5020
33	N	5.6237	1.1407	-2.2337
34	N	1.6669	-0.6725	-0.5455
35	H	1.6428	-1.2504	-1.3860
36	N	4.5834	2.0502	-0.4947
37	N	2.6035	1.1687	0.4854
38	N	3.8110	3.0263	1.3395
39	C	4.5519	1.0718	-1.4226
40	C	2.6559	0.2579	-0.5145
41	C	3.6159	2.0490	0.4468
42	C	0.4587	-0.5627	0.2542

43	H	-0.0727	-1.5221	0.1970
44	H	0.7347	-0.3694	1.3011
45	C	5.6806	0.5502	-3.5530
46	H	6.7208	0.2866	-3.7839
47	H	5.0632	-0.3552	-3.5418
48	H	5.2953	1.2569	-4.3062
49	C	2.8255	3.5150	2.2742
50	H	2.3696	4.4409	1.8894
51	H	2.0589	2.7393	2.3832
52	H	3.3018	3.7090	3.2448

$\{[UO_2(H_2O)_2]enl(VO_2)\}^-$

	Atom	X	Y	Z
1	U	-1.1676	-6.1617	-4.0281
2	O	0.2241	-4.8155	-5.2464
3	O	-2.6356	-5.4658	-4.8734
4	V	1.0421	5.8533	3.7257
5	O	2.3059	6.3355	4.6960
6	O	0.0604	7.1297	3.3247
7	O	-2.0604	-5.6757	-1.9685
8	O	0.2492	-7.0413	-3.2791
9	N	1.1854	-2.3221	-2.8898
10	N	1.0320	-3.8206	-4.6908
11	N	1.2237	-0.9904	-1.0167
12	H	1.8128	-0.3922	-1.5966
13	N	-0.3778	-4.1318	-2.9442
14	N	-0.2122	-2.7985	-0.9609
15	N	-1.5467	-4.7189	-1.0999
16	C	0.6164	-3.3813	-3.4791
17	C	0.6996	-2.0742	-1.6433
18	C	-0.6953	-3.8391	-1.6575
19	C	0.6721	-0.4427	0.2107
20	H	1.4095	0.2540	0.6331
21	H	0.5043	-1.2612	0.9252
22	C	1.6919	-2.9693	-5.6622
23	H	2.3530	-3.5858	-6.2850
24	H	2.2840	-2.2262	-5.1169
25	H	0.9448	-2.4633	-6.2955
26	C	-2.3383	-4.4627	0.0848
27	H	-3.3523	-4.1408	-0.2030
28	H	-1.8428	-3.6710	0.6572
29	H	-2.3971	-5.3794	0.6863
30	O	0.0086	4.9212	5.1676
31	O	2.1280	5.5756	2.0617
32	N	-1.0897	2.2642	3.0170
33	N	-0.8551	3.8868	4.7427
34	N	-1.1793	0.8900	1.1751
35	H	-1.7527	0.3082	1.7865
36	N	0.3868	4.1214	2.9171
37	N	0.2814	2.6731	1.0347
38	N	1.6333	4.6222	1.1520
39	C	-0.5400	3.3670	3.5423
40	C	-0.6334	1.9876	1.7608
41	C	0.7544	3.7545	1.6725
42	C	-0.6502	0.3032	-0.0443

43	H	-1.3994	-0.3978	-0.4379
44	H	-0.4864	1.1002	-0.7835
45	C	-1.4557	3.1298	5.8201
46	H	-2.0783	3.7986	6.4283
47	H	-2.0801	2.3482	5.3721
48	H	-0.6734	2.6710	6.4461
49	C	2.4911	4.3501	0.0211
50	H	3.4814	4.0171	0.3707
51	H	2.0182	3.5570	-0.5695
52	H	2.5946	5.2582	-0.5872
53	O	-2.6066	-8.2812	-3.6293
54	H	-2.1226	-9.1376	-3.6296
55	H	-3.3416	-8.4164	-4.2687
56	O	-0.8020	-7.5933	-6.1689
57	H	0.1458	-7.7717	-6.3635
58	H	-1.1408	-7.1736	-6.9915

$[UO_2(H_2O)_2]_2enl$

	Atom	X	Y	Z
1	U	-1.2461	-6.1581	-4.0253
2	O	0.1246	-4.8328	-5.2856
3	O	-2.7291	-5.3996	-4.7854
4	U	1.0108	6.2898	3.9446
5	O	2.4586	5.8045	4.9561
6	O	-0.4050	6.9742	3.0136
7	O	-2.0203	-5.6772	-1.9144
8	O	0.1755	-7.0943	-3.3592
9	N	1.2378	-2.3712	-2.9619
10	N	0.9715	-3.8512	-4.7658
11	N	1.4037	-1.0665	-1.0745
12	H	1.9679	-0.4693	-1.6798
13	N	-0.3505	-4.1603	-2.9534
14	N	-0.0774	-2.8375	-0.9742
15	N	-1.4597	-4.7276	-1.0681
16	C	0.6246	-3.4174	-3.5310
17	C	0.8201	-2.1284	-1.6900
18	C	-0.6112	-3.8649	-1.6547
19	C	0.9098	-0.5137	0.1765
20	H	1.6754	0.1667	0.5746
21	H	0.7549	-1.3329	0.8929
22	C	1.5918	-3.0010	-5.7638
23	H	2.2051	-3.6227	-6.4290
24	H	2.2271	-2.2749	-5.2452
25	H	0.8207	-2.4742	-6.3498
26	C	-2.1908	-4.4679	0.1539
27	H	-3.2126	-4.1308	-0.0837
28	H	-1.6580	-3.6858	0.7058
29	H	-2.2334	-5.3870	0.7530
30	O	-0.3673	4.9573	5.1957
31	O	2.1003	5.6704	2.0168
32	N	-1.0471	2.2382	2.9931
33	N	-1.0865	3.8799	4.6716
34	N	-0.9265	0.7864	1.2179
35	H	-1.5697	0.2246	1.7747
36	N	0.4315	4.1190	3.0055
37	N	0.4448	2.6419	1.1177
38	N	1.6787	4.6330	1.1909
39	C	-0.5643	3.3643	3.5329
40	C	-0.4800	1.9276	1.7947
41	C	0.8390	3.7510	1.7638
42	C	-0.4103	0.2515	-0.0278

43	H	-1.1645	-0.4319	-0.4425
44	H	-0.2515	1.0786	-0.7346
45	C	-1.7798	3.0788	5.6625
46	H	-2.5034	3.7135	6.1896
47	H	-2.3067	2.2730	5.1395
48	H	-1.0611	2.6525	6.3818
49	C	2.5451	4.3354	0.0697
50	H	3.5548	4.0809	0.4301
51	H	2.1157	3.4851	-0.4712
52	H	2.5967	5.2119	-0.5896
53	O	0.3807	7.8934	5.8950
54	H	0.6768	7.5820	6.7800
55	H	-0.5909	8.0123	5.9958
56	O	-2.7309	-8.2434	-3.5832
57	H	-2.2691	-9.1115	-3.6127
58	H	-3.4948	-8.3537	-4.1926
59	O	2.3049	8.4877	3.4433
60	H	2.9688	8.7467	4.1209
61	H	1.7513	9.2922	3.3250
62	O	-1.0438	-7.5756	-6.1937
63	H	-0.1182	-7.8004	-6.4400
64	H	-1.4085	-7.1405	-6.9970

pdl^{4-}

	Atom	X	Y	Z
1	O	-0.6191	2.7950	-5.1169
2	N	0.0003	0.2337	-5.7557
3	N	-0.5926	1.8108	-4.1686
4	N	-0.3243	-0.3758	-3.4115
5	O	0.6074	-0.5512	-8.2799
6	N	0.3390	-2.0624	-5.0065
7	N	0.6118	-1.4372	-7.2370
8	C	-0.2942	0.5291	-4.4611
9	C	-0.9203	2.2584	-2.8264
10	H	-0.2290	3.0711	-2.5468
11	H	-1.9492	2.6581	-2.8175
12	H	-0.8358	1.4359	-2.1111
13	C	0.0071	-1.6121	-3.7838
14	C	0.3072	-1.0761	-5.9735
15	C	0.0311	-2.5390	-1.4250
16	C	0.4946	-3.6601	-0.7042
17	H	0.8304	-4.5474	-1.2461
18	C	0.9418	-2.8023	-7.6044
19	H	0.1946	-3.1745	-8.3271
20	H	1.9303	-2.8099	-8.0944
21	H	0.9569	-3.4467	-6.7192
22	C	-0.4049	-1.4266	-0.6856
23	H	-0.7851	-0.5623	-1.2196
24	O	-0.4957	2.7944	5.1483
25	N	0.1917	0.2459	5.7661
26	N	-0.4515	1.8172	4.1931
27	N	-0.1465	-0.3598	3.4230
28	O	0.8458	-0.5347	8.2788
29	N	0.5614	-2.0415	5.0046
30	N	0.8519	-1.4182	7.2337
31	C	-0.1253	0.5401	4.4767
32	C	-0.8538	2.2521	2.8670
33	H	-1.9107	2.5709	2.8917
34	H	-0.2354	3.1181	2.5789
35	H	-0.7281	1.4464	2.1388
36	C	0.2104	-1.5920	3.7864
37	C	0.5257	-1.0589	5.9749
38	C	0.1413	-2.5244	1.4266
39	C	-0.3486	-1.4182	0.7108
40	H	-0.6852	-0.5481	1.2639
41	C	1.2060	-2.7790	7.5942
42	H	2.2002	-2.7735	8.0726

43	H	0.4729	-3.1634	8.3250
44	H	1.2199	-3.4214	6.7075
45	C	0.5486	-3.6532	0.6835
46	H	0.9245	-4.5356	1.2071
47	N	0.2244	-2.6002	2.8179
48	H	0.5882	-3.4794	3.1888
49	N	0.0038	-2.6278	-2.8199
50	H	0.3579	-3.5061	-3.2033

[(VO$_2$)$_2$pdl]$^{2-}$

	Atom	X	Y	Z
1	V	0.2800	1.4073	-7.2644
2	O	0.0288	2.4776	-5.5756
3	N	0.1858	0.0639	-5.7606
4	N	0.2581	1.7915	-4.3722
5	N	0.3001	-0.3854	-3.4272
6	O	-0.0163	-0.3736	-8.1317
7	N	0.2727	-2.2023	-5.0554
8	N	0.2386	-1.5039	-7.3299
9	C	0.2428	0.4589	-4.4731
10	C	-0.0093	2.5426	-3.1668
11	H	0.6331	3.4324	-3.1445
12	H	-1.0687	2.8418	-3.1351
13	H	0.2225	1.8972	-2.3125
14	C	0.3088	-1.6813	-3.7963
15	C	0.2242	-1.2621	-6.0103
16	C	0.4096	-2.5201	-1.4252
17	C	0.8400	-3.6494	-0.7043
18	H	1.1359	-4.5530	-1.2404
19	C	-0.1003	-2.7690	-7.9426
20	H	-1.1722	-2.7925	-8.1968
21	H	0.5032	-2.9037	-8.8494
22	H	0.1297	-3.5624	-7.2225
23	C	0.0269	-1.3778	-0.7080
24	H	-0.3205	-0.4998	-1.2424
25	O	1.8414	1.7704	-7.6903
26	O	-0.8159	2.2533	-8.1868
27	O	-1.3024	2.1421	5.6429
28	N	-0.1274	0.0231	5.7582
29	N	-1.0377	1.5282	4.4102
30	N	-0.2530	-0.4911	3.4392
31	O	0.2989	-0.3043	8.1191
32	N	0.7771	-2.0434	5.0142
33	N	0.8479	-1.2807	7.2658
34	C	-0.4739	0.3204	4.4904
35	C	-1.7926	2.0350	3.2877
36	H	-2.8710	1.9232	3.4804
37	H	-1.5466	3.0935	3.1311
38	H	-1.5102	1.4536	2.4038
39	C	0.3788	-1.6323	3.7773
40	C	0.5024	-1.1511	5.9765
41	C	0.5361	-2.4753	1.4029
42	C	0.0947	-1.3528	0.6859

43	H	-0.1973	-0.4568	1.2218
44	C	1.1969	-2.5275	7.9086
45	H	1.9552	-2.3368	8.6786
46	H	0.3036	-2.9834	8.3647
47	H	1.6068	-3.1956	7.1424
48	C	0.8984	-3.6290	0.6808
49	H	1.2384	-4.5178	1.2157
50	V	-0.3288	1.3839	7.2352
51	O	-1.4874	1.7732	8.3632
52	O	0.9938	2.3771	7.3564
53	N	0.6721	-2.5396	2.7949
54	H	1.1167	-3.3858	3.1556
55	N	0.3563	-2.6397	-2.8204
56	H	0.4501	-3.5871	-3.1907

{[$UO_2(H_2O)_2$]pdl(VO_2)}$^-$

	Atom	X	Y	Z
1	V	7.0192	-0.0725	-0.5164
2	O	5.2737	-0.3720	-1.4761
3	N	5.6052	-0.1788	0.9207
4	N	4.1119	-0.1660	-0.7176
5	N	3.3035	-0.0753	1.5103
6	O	8.0030	-0.3172	1.2143
7	N	5.0394	-0.0382	3.2239
8	N	7.2668	-0.0541	2.3875
9	C	4.2946	-0.1452	0.6044
10	C	2.8634	-0.4337	-1.3929
11	H	2.7629	0.2373	-2.2565
12	H	2.8339	-1.4827	-1.7265
13	H	2.0536	-0.2455	-0.6795
14	C	3.7518	-0.0342	2.7796
15	C	5.9348	-0.0967	2.2266
16	C	1.4273	0.0370	3.6991
17	C	0.7135	0.6936	4.7149
18	H	1.2553	1.2025	5.5145
19	C	7.9579	-0.3698	3.6186
20	H	8.2350	-1.4361	3.6332
21	H	8.8582	0.2529	3.6993
22	H	7.2782	-0.1499	4.4496
23	C	0.7102	-0.6222	2.6898
24	H	1.2449	-1.1648	1.9156
25	O	7.3890	1.4910	-0.9280
26	O	7.9129	-1.1623	-1.4005
27	O	-5.0817	-0.1220	-1.6139
28	N	-5.5597	0.2168	0.8050
29	N	-4.0072	-0.2910	-0.7536
30	N	-3.2736	-0.0879	1.4600
31	O	-7.9753	0.6021	1.2075
32	N	-4.9896	0.2925	3.1291
33	N	-7.1986	0.4232	2.3489
34	C	-4.2560	-0.0463	0.5396
35	C	-2.6995	-0.3264	-1.3703
36	H	-2.6399	-1.1893	-2.0476
37	H	-2.5293	0.6028	-1.9366
38	H	-1.9521	-0.4226	-0.5762
39	C	-3.7102	0.1100	2.7138
40	C	-5.8748	0.3185	2.1206
41	C	-1.3943	0.0888	3.6712
42	C	-0.6824	-0.5964	2.6750

43	H	-1.2218	-1.1198	1.8917
44	C	-7.7867	0.9106	3.5787
45	H	-7.9850	1.9919	3.5021
46	H	-8.7259	0.3725	3.7631
47	H	-7.0813	0.7167	4.3939
48	C	-0.6755	0.7184	4.7017
49	H	-1.2130	1.2472	5.4912
50	U	-7.2707	0.1251	-0.9220
51	O	-7.4347	-1.6701	-0.6274
52	O	-7.2425	1.9022	-1.3559
53	O	-9.8418	0.2377	-1.2198
54	H	-10.2371	1.1140	-1.0110
55	H	-10.3411	-0.3936	-0.6541
56	O	-7.5688	-0.3511	-3.4503
57	H	-7.1564	0.3164	-4.0438
58	H	-7.1896	-1.2080	-3.7499
59	N	-2.7949	0.1379	3.7334
60	H	-3.1960	0.4175	4.6304
61	N	2.8275	0.0217	3.7879
62	H	3.2297	0.2025	4.7089

$[UO_2(H_2O)_2]_2$pdl

	Atom	X	Y	Z
1	U	7.4768	-0.3548	-0.8144
2	O	5.3266	-0.7484	-1.5736
3	N	5.7060	-0.3091	0.8528
4	N	4.1919	-0.5368	-0.8070
5	N	3.3637	-0.2303	1.3602
6	O	8.1173	-0.1039	1.3680
7	N	5.0116	0.1761	3.0902
8	N	7.2558	0.2447	2.4080
9	C	4.3957	-0.3602	0.5035
10	C	2.9298	-0.8655	-1.4314
11	H	2.8257	-0.2820	-2.3561
12	H	2.8906	-1.9416	-1.6631
13	H	2.1264	-0.6028	-0.7362
14	C	3.7457	0.0129	2.6234
15	C	5.9488	0.0259	2.1436
16	C	1.3948	0.0628	3.5280
17	C	0.6784	0.4872	4.6631
18	H	1.2178	0.8650	5.5337
19	C	7.8375	0.1612	3.7341
20	H	8.1752	-0.8681	3.9367
21	H	8.6890	0.8513	3.7930
22	H	7.0717	0.4534	4.4612
23	C	0.6748	-0.4324	2.4295
24	H	1.2092	-0.7902	1.5570
25	O	7.2678	1.4471	-1.0270
26	O	7.8153	-2.1511	-0.7381
27	O	-5.2651	0.3717	-1.5978
28	N	-5.6875	0.3237	0.8598
29	N	-4.1582	0.1575	-0.7928
30	N	-3.3681	0.0551	1.4068
31	O	-8.1060	0.4714	1.3635
32	N	-5.0491	0.0738	3.1530
33	N	-7.2881	0.1757	2.4525
34	C	-4.3795	0.1810	0.5261
35	C	-2.8681	0.2435	-1.4392
36	H	-2.7904	-0.5398	-2.2059
37	H	-2.7511	1.2339	-1.9062
38	H	-2.0967	0.0981	-0.6766
39	C	-3.7742	0.0304	2.6852
40	C	-5.9653	0.2003	2.1810
41	C	-1.4310	-0.0237	3.5734
42	C	-0.7183	-0.4782	2.4549

43	H	-1.2555	-0.8730	1.5987
44	C	-7.8601	0.4889	3.7470
45	H	-8.1011	1.5629	3.8052
46	H	-8.7722	-0.1056	3.8869
47	H	-7.1253	0.2288	4.5167
48	C	-0.7084	0.4457	4.6847
49	H	-1.2427	0.7935	5.5707
50	U	-7.4444	0.3172	-0.8222
51	O	-7.4273	-1.5076	-0.7466
52	O	-7.5971	2.1283	-1.0328
53	O	-10.0229	0.2139	-1.0558
54	H	-10.4946	0.9954	-0.6891
55	H	-10.4395	-0.5541	-0.6034
56	O	-7.7452	0.1258	-3.3900
57	H	-7.3892	0.8915	-3.8946
58	H	-7.2995	-0.6548	-3.7901
59	O	7.7613	-0.5758	-3.3839
60	H	7.1835	0.0286	-3.9029
61	H	7.5387	-1.4715	-3.7249
62	O	10.0222	-0.0076	-1.1244
63	H	10.3575	0.8583	-0.7989
64	H	10.5885	-0.6725	-0.6718
65	N	-2.8296	-0.0516	3.6745
66	H	-3.2154	0.0582	4.6139
67	N	2.7907	0.1395	3.5961
68	H	3.1786	0.3918	4.5071

$[VO_2(H_2O)_3]^+$

	Atom	X	Y	Z
1	V	-0.0170	-0.1836	0.0000
2	H	-2.5134	-0.2130	0.7806
3	H	0.4852	2.3430	0.7799
4	O	-0.0116	-1.1245	1.3152
5	H	2.4897	-0.2795	-0.7805
6	O	-0.0116	-1.1245	-1.3152
7	O	-2.0848	0.2167	0.0000
8	H	-2.5134	-0.2130	-0.7806
9	O	0.0124	1.9599	0.0000
10	H	0.4852	2.3430	-0.7799
11	O	2.0767	0.1658	0.0000
12	H	2.4897	-0.2795	0.7805

$[UO_2(H_2O)_5]^{2+}$

	Atom	X	Y	Z
1	U	0.0230	0.0241	-0.1144
2	H	0.5306	0.8921	2.7996
3	H	1.3150	-0.7074	-2.7980
4	H	-2.4284	0.8210	1.5862
5	O	0.0727	-1.7517	-0.0360
6	H	0.5396	-0.6755	2.8432
7	H	-1.8325	-0.5240	-2.4974
8	H	2.9810	-0.6549	0.3103
9	O	0.0243	1.8029	-0.1285
10	O	0.6703	-0.0223	-2.5036
11	H	0.9811	0.8162	-2.9181
12	O	2.4834	0.0574	-0.1548
13	H	2.9514	0.8934	0.0755
14	O	0.6976	0.0931	2.2481
15	O	-1.9688	-0.0302	-1.6554
16	H	-2.7907	-0.4041	-1.2608
17	O	-2.1002	0.0101	1.1325
18	H	-2.3349	-0.7378	1.7295

$H_2VO_4^-$

	Atom	X	Y	Z
1	V	-0.0000	-0.0000	-0.1470
2	H	1.9350	-0.5438	1.1452
3	O	-0.2202	1.3497	-1.0777
4	H	-1.9350	0.5438	1.1452
5	O	0.2202	-1.3497	-1.0777
6	O	1.4951	0.3071	0.9207
7	O	-1.4951	-0.3071	0.9207

$[UO_2(OH)_3]^-$

	Atom	X	Y	Z
1	U	0.2130	-0.0186	-0.1101
2	H	0.4401	0.7996	2.5622
3	H	-2.6481	0.7323	1.2864
4	O	0.1070	-1.8564	-0.1329
5	H	-1.3533	-0.0396	-2.2814
6	O	0.1929	1.8202	-0.0943
7	O	0.3686	-0.0751	-2.4027
8	H	0.5997	0.8102	-2.7582
9	O	2.4182	-0.0543	-0.0335
10	H	2.8257	0.8363	0.0053
11	O	0.2158	-0.0813	2.1925
12	O	-2.1334	0.0913	-1.6518
13	H	-2.5512	-0.7952	-1.5787
14	O	-2.2420	-0.1620	1.3234
15	H	-1.4742	-0.0672	1.9762

References

(1) Sheldrick, G. M. SHELXL-97: Program for the Refinement of Crystal Structure. University of Göttingen: Göttingen, Germany, 1997. Sheldrick, G. M. SHELXS-97: Program for the Solution of Crystal Structure. University of Göttingen: Göttingen, Germany, 1997.

(2) Zhang, Q.; Fu, S.; Li, H.; Liu, Y. A novel method for the determination of hydrogen peroxide in bleaching effluents by spectroscopy. *BioResources* **2013**, *8* (3), 3699-3705, Article. DOI: 10.15376/biores.8.3.3699-3705 Scopus.

(3) *AMS-2020.101*; 2020 http://www.scm.com, *Software for Chemistry and Materials*. (accessed.

(4) Zhang, Y.; Yang, W. Comment on ``Generalized Gradient Approximation Made Simple''. *Phys. Rev. Lett.* **1998**, *80* (4), 890-890. DOI: 10.1103/PhysRevLett.80.890.

(5) Perdew, J. P.; Burke, K.; Ernzerhof, M. Generalized gradient approximation made simple. *Phys. Rev. Lett.* **1996**, *77*, 3865-3868. DOI: 10.1103/PhysRevLett.77.3865.

(6) Caldeweyher, E.; Bannwarth, C.; Grimme, S. Extension of the D3 dispersion coefficient model. *J. Chem. Phys.* **2017**, *147* (3), 034112. DOI: 10.1063/1.4993215.

(7) Klamt, A.; Schüürmann, G. COSMO: a new approach to dielectric screening in solvents with explicit expressions for the screening energy and its gradient. *J. Chem. Soc., Perkin Trans. 2* **1993**, (5), 799-805.
